# Visual security defense for industrial inspection based on computer vision

**DOI:** 10.1371/journal.pone.0338835

**Published:** 2026-02-04

**Authors:** Zhihao Jiang, Haotian Yuan, Chenrui Zeng, Liu Fu

**Affiliations:** Yangtze University, Jingzhou, Hubei, China; Donghua University, CHINA

## Abstract

As intelligent manufacturing advances, computer vision-based defect detection systems have become essential components of industrial automation. However, this progress has also revealed new security vulnerabilities. In this work, we identify and examine a stealthy adversarial vector—the Alpha Channel Attack—which exploits the often-ignored transparency layer in RGBA images to inject imperceptible perturbations, thereby evading both human perception and conventional preprocessing defenses.We evaluate this threat across diverse model architectures, including YOLOv5, FastGAN, and state-of-the-art vision-language models such as DeepSeek-VL2, ChatGPT-4o, and KIMI. Experimental results show that alpha-channel perturbations cause substantial degradation in detection, generation, and multimodal alignment metrics—including mAP, FID, BLEU, METEOR, and CLIP Score—while leaving the visible image content unchanged.To mitigate this invisible yet high-impact risk, we propose a lightweight detection mechanism that integrates histogram overlap and MSE analysis within the alpha channel. The framework achieves an AUC of 0.998, demonstrating strong capability in identifying adversarial samples under real-world constraints.Overall, this study reveals a critical blind spot in modern visual data pipelines and introduces both a novel threat model and an effective defense strategy, contributing to the development of more resilient industrial AI systems.

## 1. Introduction

In recent years, model poisoning and adversarial attacks have increasingly become focal points in the field of machine learning security, particularly within the context of data poisoning. This reflects the growing concern in both academia and industry over the security and integrity of machine learning models. Existing literature has systematically explored various data poisoning attack strategies and their potential impacts, revealing the risk that minor perturbations in training data can significantly compromise model performance. This paper presents a comprehensive analysis of related research findings, aiming to clarify the primary methods, mechanisms, and real-world security risks associated with data poisoning attacks.Solans et al. [1] emphasized the impact of poisoning attacks on algorithmic fairness, introducing an optimization framework specifically designed to address classification disparities among different demographic groups. Their work underscores that while model accuracy often takes center stage, broader impacts on fairness metrics warrant further research. This perspective is crucial, as it highlights the multifaceted nature of model performance and the potential for adversarial attacks to exacerbate existing biases in machine learning systems.In the field of graph neural networks, Sun et al. [2] proposed a novel node injection poisoning attack method using hierarchical reinforcement learning. Their findings indicate that this method is significantly more effective than traditional node injection techniques, demonstrating the potential to deliberately manipulate graph data structures. This contributes to understanding how structural changes in data can be leveraged to compromise model integrity.Zhang et al. [3] introduced PoisonGAN, a generative approach to data poisoning within federated learning environments. Their exploration of poisoning mechanisms highlights the unique challenges posed by federated systems, where data is distributed across multiple devices. The study advances the theoretical framework for poisoning attacks and offers practical insights for mitigating risks in federated learning.Geiping et al. [4] demonstrated that large-scale datasets, such as ImageNet, can be effectively poisoned to compromise neural networks trained from scratch. Their work underscores the scalability of poisoning attacks and raises alarms about the inherent vulnerabilities of machine learning models deployed in real-world applications.The development of defenses against such attacks is also a critical area of study. Chen et al. [5] proposed De-Pois, an attack-agnostic defense mechanism utilizing GANs for data augmentation and model simulation. This method aims to enhance model resilience against various poisoning strategies, demonstrating a proactive stance in the ongoing arms race between attackers and defenders in the machine learning field.

Wallace et al. [6] focused on covert data poisoning attacks in natural language processing models, illustrating how adversaries can manipulate model predictions by subtly inserting poisoned examples. Their findings reveal the subtlety of data poisoning, especially in sensitive applications such as sentiment analysis.In the context of recommendation systems, Zhang et al. [7] addressed vulnerabilities arising from incomplete and perturbed user-item interaction data. Their adversarial attack method underscores the need for robust defenses in systems heavily reliant on user-generated content, further emphasizing the pervasiveness of data poisoning threats.

Gosselin et al. [8] conducted a comprehensive survey of privacy and security issues in federated learning, including risks posed by data poisoning. This work places the discussion within a broader security context, reinforcing the notion that data poisoning is a critical concern capable of leading to biased model predictions.Finally, Yang et al. [9] investigated clean-label poisoning attacks in federated learning, particularly within Internet of Things (IoT) environments. Their research highlights the adaptability of adversarial strategies and the ongoing need for vigilance in safeguarding federated frameworks against complex attacks.

The concept of utilizing transparency as an adversarial medium gained momentum between 2024 and 2025. McKee and Noever demonstrated that adversarial manipulations in the alpha channel can trigger erroneous predictions in neural vision models without altering human perception [10]. Noever et al. further validated these attacks in recommender and satellite systems, revealing cross-domain vulnerabilities [11]. The AlphaDog framework formally identified this as a structural flaw in visual pipelines, wherein transparency layers are discarded without semantic validation [12].

In parallel, Kadhim developed a GAN-based video encryption model using a two-dimensional extended Schaffer function, capable of selectively securing critical regions in video frames [16]. Meanwhile, Alsaify and Mahmod introduced temporal action segmentation to dynamically isolate and encrypt sensitive video segments [17]. In the image forensics domain, Wang et al. proposed a GAN-based anti-forensics method that manipulates JPEG quantization tables in the header file to evade forensic tracing while preserving the visual content [18]. These methods share a common theme: exploiting non-RGB channels or metadata structures to covertly interfere with model perception and post-processing logic.

To mitigate such threats, recent defensive techniques include test-time prompt tuning (R-TPT) [13], channel-aware denoising filters [14], and diffusion-based universal defenses (UAD-RS) [15]. However, these approaches remain largely disconnected from alpha-layer poisoning, which can persist through image transformations and evade dataset sanitization.More recently, chaotic-map methods have been applied to multi-dimensional encryption: a 3D memristive cubic map with dual discrete memristors was introduced for secure image encryption [38], a parallel color encryption algorithm was designed based on a 2D Logistic-Rulkov neuron map [39], and a three-dimensional memristor-based hyperchaotic map was proposed for pseudorandom number generation and multi-image encryption [40].

In summary, current research reveals a highly complex and evolving landscape of data poisoning attacks, posing significant threats to the integrity and reliability of machine learning models across diverse domains. These studies not only dissect commonly used attack techniques and strategies but also propose initial defense mechanisms, underscoring the many challenges still facing the field in terms of security. Consequently, effectively identifying and defending against potential data poisoning during the model training phase has become a core issue in current machine learning security research.

Based on this, the present study starts from theoretical modeling, focusing on the perceptual differences caused by disguised data channels. It further proposes a security detection framework oriented toward visual deception prevention and systematically analyzes its applicability and robustness in industrial inspection scenarios.

## 2. Theoretical analysis

With the widespread adoption of deep learning in industrial vision inspection, models such as YOLO and Generative Adversarial Networks (GANs) have become fundamental components of intelligent detection systems. However, when processing high-dimensional image data, these models often exhibit insufficient sensitivity to input channel structures, particularly with respect to transparency information. This overlooked limitation introduces new security vulnerabilities, especially in systems that process RGBA images. The Alpha channel—an essential component encoding pixel transparency—has long been ignored during model training and inference, thereby creating a potential adversarial vector.

Recent studies indicate that adversaries can inject imperceptible perturbations into the Alpha channel, significantly impairing model decision-making while leaving human visual perception unchanged. For example, Xia and Chen proposed AlphaDog, demonstrating that alpha-only perturbations can consistently mislead deep models without altering visible content [41]. McKee et al. further revealed that transparency-layer manipulations can propagate through model feature hierarchies and create significant discrepancies between human and AI perception [42]. This vulnerability is particularly critical in industrial defect detection, where YOLO-based detection networks and GAN-based data augmentation models remain highly susceptible to transparency-layer perturbations. Such attacks can reduce detection accuracy, distort synthesized outputs, and undermine model reliability and generalization.

Beyond transparency, adversarial research has increasingly explored non-RGB domains. Perturbations in alternative color spaces—such as YCbCr or frequency-domain components—have been shown to retain high stealthiness while degrading model performance [43]. Noever and McKee also reported that Alpha-channel perturbations can mislead cross-domain systems such as recommender engines, suggesting that transparency-based vulnerabilities represent a broad and underexamined attack surface [44]. Collectively, these findings reveal that modern deep models insufficiently model auxiliary channels, leaving transparency information as a critical blind spot.

Motivated by these observations, this paper examines both the theoretical foundations and practical mechanisms of Alpha-channel attacks. We propose a perturbation simulation method to evaluate their impact on YOLO and GAN training, enabling controlled comparisons of model performance before and after transparency manipulation. Experimental results demonstrate the detrimental influence of Alpha perturbations on robustness and prediction accuracy. Furthermore, we design a lightweight detection strategy based on histogram overlap and MSE analysis and explore preliminary defense mechanisms tailored to transparency-layer adversarial samples. Overall, this research analyzes the mathematical principles and propagation pathways of Alpha-channel attacks, providing the first systematic assessment of their practical threat in industrial inspection systems and contributing to the design of safer and more robust visual models.

### 2.1 Principle of alpha channel attack

Modern computer vision systems often represent images in RGBA format, where the Alpha channel denotes pixel transparency, with values ranging from 0 (fully transparent) to 1 (fully opaque). Since transparency exerts limited influence on perceived visual content, most deep learning pipelines ignore, discard, or blend this channel with a background color. This oversight creates a structural blind spot.

An Alpha Channel Attack (ACA) exploits this vulnerability by perturbing the Alpha channel while leaving RGB channels unchanged, misleading the model’s feature extraction and classification processes without altering the image’s visible content.

The central idea is to modify only the Alpha channel of an image X=(R,G,B,A),By introducing a perturbation $\delta_{\text{A}}$,the adversarial image becomes:


$$\dot{{\text{X}}^{\text{adv}}}=\left(\text{R},\text{G},\text{B},\text{A}+\delta_{\text{A}}\right)$$ (1)

A deep model f(·) receives this image and outputs a label prediction $\hat{\text{y}}={\text{f}(\text{X}}^{\text{a}\text{d}\text{v}})$. The attack objective is to cause misclassification, i.e., $\hat{\text{y}}\neq \text{y}$. This can be formalized as an optimization problem:


$$\underset{\delta }{\text{maximize}}L\left(\text{f}\left({\text{X}}^{\prime }\right),\text{y}\right)\;\text{s}.\text{t}.∥\delta ∥\leq \varepsilon$$ (2)

where L is the loss function (commonly cross-entropy), and ∥δ∥ is the norm constraint on the alpha channel perturbation, usually using ${\text{L}}_{\text{2}}$ or ${\text{L}}_{\infty }$ norms. ε controls the maximum amplitude of perturbation. Since the alpha channel is usually not explicitly handled during model training, perturbations on it do not cause significant perceptual changes but can effectively disrupt the inference process, showing high attack efficacy and stealth.

From the perspective of attack methods, alpha channel attacks can be classified into three types: white-box attacks, black-box attacks, and hybrid attacks. In white-box attacks, the attacker has full access to the model structure and gradient information, and commonly uses gradient-based methods to generate perturbations, such as Fast Gradient Sign Method (FGSM) and Projected Gradient Descent (PGD). For example, FGSM can be represented as:


$$\delta =\varepsilon \cdot \text{sign}\left(\nabla_{\text{A}}L\left(\text{f}(\text{X}),\text{y}\right)\right)$$ (3)

i.e., generating maximum perturbation in the direction of the loss gradient with respect to the alpha channel. In black-box scenarios, where model internals are inaccessible, attackers rely on model output or transfer-based methods to craft perturbations. In addition, heuristic methods based on image entropy or salient regions can be used to generate perturbation areas, enhancing real-world adaptability. Hybrid attacks jointly optimize perturbations in RGB and alpha channels, balancing attack strength and stealth by setting different constraints. The perturbation vector is denoted as $(\delta_{\text{RGB}},\delta_{\text{A}}$), satisfying the following conditions:


$$\begin{array}{c} ∥\delta_{\text{RGB}}∥\leq \varepsilon_{\text{1}},\;∥\delta_{\text{A}}∥\leq \varepsilon_{\text{2}} \end{array}$$ (4)

Compared with traditional RGB channel attacks, alpha channel attacks show several unique advantages. First, at the visual level, alpha perturbations are nearly imperceptible and do not attract user attention during image display. Second, since model architectures typically do not establish dedicated paths or explicit modeling for the alpha channel, network robustness to alpha perturbations is extremely weak. Third, this attack strategy can be combined with mainstream adversarial algorithms to further enhance attack strength. Fourth, it has broad applicability across multiple visual tasks such as classification, detection, and segmentation, demonstrating strong transferability and generalization.

**Fig 1:**
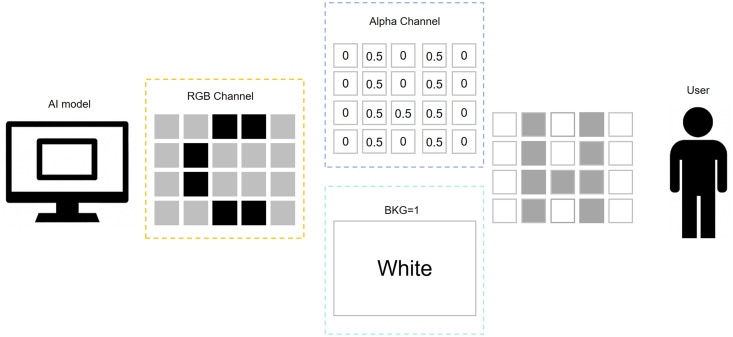
Illustration of human-AI perception discrepancy in alpha channel attacks.

As shown in Fig 1, this image presents a conceptual example of an alpha channel attack. In this case, the attacker manipulates the alpha channel to create an image that appears differently to human observers and AI models. The left side shows the AI model’s output, recognizing the image as the letter “C.” This is because the AI model, which ignores the alpha channel and only processes the RGB channels, interprets black pixels arranged in a “C” shape. The center shows the alpha channel, which contains transparency information ranging from 0 (fully transparent) to 0.5 (semi-transparent). The right side shows the human observer’s view. Since the image is overlaid on a white background, the observer perceives a white background with partially transparent and transparent pixels forming the letter “H.” This is due to the human visual system combining transparency with background color, creating a different perceptual outcome.

In recent years, alpha channel attacks have become a hot topic in adversarial research, expanding in multiple directions. One notable direction is their application in multimodal systems. In image-text models (e.g., CLIP) and cross-modal reasoning systems, alpha perturbations may affect the entire inference chain through the image modality.

**Fig 2:**
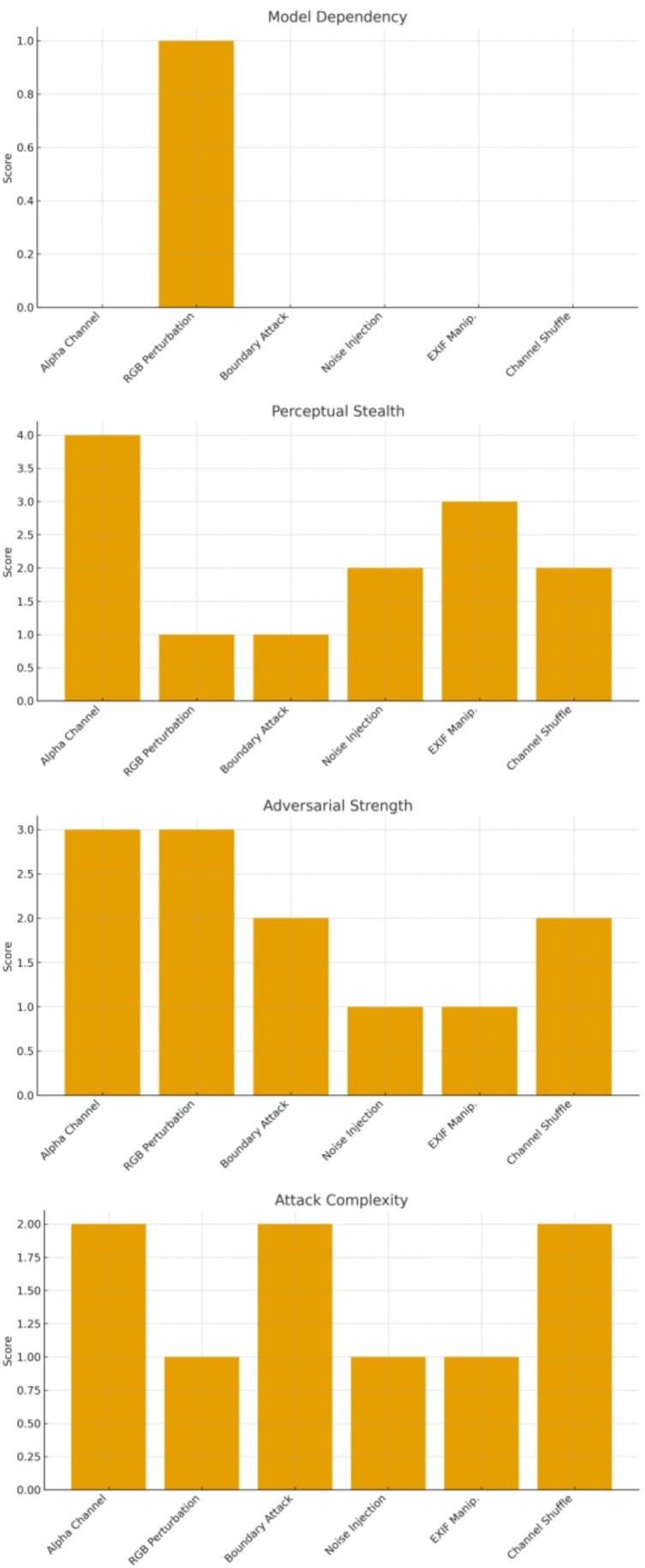
Comparative evaluation of adversarial attack methods across four key criteria.

Fig 2 provides a comprehensive comparison of the proposed alpha-channel attack against several representative adversarial techniques across four key criteria: attack complexity, perceptual stealth, adversarial strength, and model dependency. The results clearly show that the alpha-channel attack occupies a unique position among existing methods. Although its implementation complexity is moderate, comparable to boundary attacks and channel-shuffling strategies, it significantly outperforms all other techniques in perceptual stealth. By embedding perturbations into the transparency layer—an input dimension largely ignored by conventional preprocessing pipelines—the attack achieves the highest invisibility score, remaining imperceptible to both human observers and machine vision systems.

In terms of adversarial strength, the alpha-channel attack delivers performance degradation on par with classical RGB-based perturbations while maintaining substantially higher stealth. This combination of invisibility and potency enables the perturbations to propagate effectively across diverse tasks, including object detection, image generation, and multimodal reasoning. Furthermore, unlike gradient-based RGB perturbations that require full model access, the alpha-channel attack operates entirely under a black-box assumption, exhibiting zero model dependency. This greatly increases its real-world applicability, allowing adversaries to compromise systems without knowledge of model parameters or architecture.

Overall, the multi-dimensional comparison highlights a critical blind spot in current defense strategies: transparency information. The alpha-channel attack simultaneously achieves high stealth, strong attack capability, and no reliance on model internals, making it a particularly challenging and potent threat vector. These findings underscore the urgent need to incorporate transparency-channel modeling into industrial and multimodal vision systems to enhance their robustness against emerging adversarial threats.

### 2.2 Generative Adversarial Networks (GANs)

Generative Adversarial Networks (GANs) have become a key technology in image generation, achieving notable progress since their introduction in 2014. Existing literature shows a wide range of applications and methods aimed at enhancing the ability of GANs to generate high-quality images. Guo [18] highlighted the interdisciplinary nature of image generation technologies, emphasizing their integration with fields such as physics, chemistry, and computer science. This interdisciplinary foundation is essential for mastering various image processing techniques and supports the effective implementation of GAN-based models.

The evolution of GANs has led to the development of specialized models tailored for specific tasks. For instance, Kimura [19] proposed a conditional GAN for zero-shot video generation, employing motion–content disentanglement to enhance generative performance. This work demonstrates the adaptability of GANs in extending beyond static image synthesis to dynamic content generation.

Ozcelik et al. [20] expanded the application of GANs by reconstructing images from fMRI patterns using an instance-conditioned GAN (IC-GAN). Their model integrates high-dimensional instance features with noise vectors, enabling the generation of images that preserve both semantic meaning and fine-grained visual details. This work highlights the potential of GANs in bridging neural activity and visual reconstruction.

Li et al. [21] provided a comprehensive overview of different GAN architectures and their applications, including text-to-image and image-to-image synthesis. Their review traces the evolution from foundational GAN models to advanced variants, reflecting a sustained research effort aimed at improving generative fidelity and controllability.

Recent developments have incorporated attention mechanisms and residual structures into GAN architectures. Wang et al. [22] optimized feature weight parameters to improve label-guided image generation, reflecting a trend toward more sophisticated network design. Huang [23] explored the use of vision transformers within GAN frameworks, noting both their potential and the challenges associated with transformer-based generators. These efforts indicate continued experimentation with architectural innovations to enhance image quality.

The development of intuitive tools has also become a significant direction in GAN research. Wei et al. [24] introduced CanvasPic, an interactive tool for flexible facial image generation. This system addresses limitations in existing GAN-based tools and underscores the importance of user-friendly interfaces for broadening access to GAN technologies.

In summary, the literature on GAN-based image generation reflects a rapidly evolving field characterized by innovative architectures, interdisciplinary methods, and practical applications that continue to advance the capabilities of visual synthesis and image understanding.

### 2.3 Object detection

Since its introduction, the YOLO (You Only Look Once) framework has undergone continuous refinement and has become a cornerstone of real-time object detection across numerous applications. Recent studies have focused on advancing detection accuracy, speed, and robustness, demonstrating the versatility of YOLO-based architectures.

Gai et al. [25] proposed an enhanced YOLOv4 model specifically designed for cherry fruit detection. Their approach integrates the CSPDarknet53 backbone with DenseNet to increase feature density and utilizes circular anchor boxes that better match the shape of cherries. This work highlights the adaptability of YOLO in addressing domain-specific challenges in agricultural environments.

Similarly, Lawal et al. [26] developed an improved YOLOv3 variant, YOLO-Tomato, for tomato detection in complex settings. By incorporating the “label what you see” annotation strategy, spatial pyramid pooling, and the Mish activation function, their model achieved a high average precision (AP), with the best variant reaching 99.5%. This demonstrates the robustness of YOLO models in diverse agricultural tasks.

In remote sensing, Wu et al. [27] combined local fully convolutional networks with the YOLO algorithm to improve small-object detection. Their work emphasizes the importance of multi-scale anchor mechanisms, demonstrating YOLO’s effectiveness in scenarios with varying object sizes and complex spatial backgrounds.

Tan et al. [28] conducted a comparative study of YOLOv3, RetinaNet, and SSD for real-time pill identification in hospital pharmacies. Their results showed that YOLOv3 achieved superior detection speed while maintaining competitive mean average precision (mAP), reinforcing its suitability for time-sensitive applications in the medical domain.

To address environmental challenges, Liu et al. [29] introduced the Image-Adaptive YOLO (IA-YOLO) framework, which applies adaptive image enhancement to improve detection performance in adverse weather conditions. This innovation demonstrates YOLO’s potential for robust deployment in low-visibility or dynamically changing environments.

Terven et al. [30] provided a comprehensive review tracing the evolution of YOLO from its earliest versions to YOLOv8 and beyond. Their analysis underscores YOLO’s significant influence across domains such as robotics, autonomous driving, and video surveillance, highlighting its central role in modern object detection research.

### 2.4 DeepSeek large models

In recent years, extensive literature on the DeepSeek framework has documented major advancements in the development and application of large language models (LLMs) and their specialized variants. These studies highlight DeepSeek’s contributions across model scaling, architecture design, domain-specific reasoning, code intelligence, and vision–language integration.

Bi et al. [31] introduced the foundational DeepSeek LLM, emphasizing the importance of scaling laws in open-source configurations. Their work demonstrated that supervised fine-tuning and direct preference optimization significantly improved conversational performance, ultimately leading to the development of DeepSeek-Chat. This effort laid the groundwork for subsequent innovations in the DeepSeek ecosystem.

Building on this foundation, Dai et al. [32] proposed DeepSeekMoE, a Mixture-of-Experts (MoE) architecture designed to achieve expert specialization. Their results showed that scaling DeepSeekMoE to 145 billion parameters outperformed earlier MoE architectures such as GShard while maintaining competitive performance with the more compact 67-billion-parameter version. This suggests an efficient path toward balancing computational cost and model capability.

In the domain of programming, Guo et al. [33] introduced the DeepSeek-Coder series to address the limitations of closed-source coding models. Their open-source models, trained on extensive code corpora, represent a major step toward democratizing access to advanced programming intelligence. This initiative supports both research and industry applications, extending the impact of DeepSeek technologies.

Mathematical reasoning, a long-standing challenge for LLMs, was explored by Shao et al. [34] through DeepSeekMath. By pretraining on large-scale mathematical token corpora, the model demonstrated improved reasoning ability and task-specific accuracy, showcasing DeepSeek’s adaptability to specialized domains.

Lu et al. [35] advanced multimodal understanding through DeepSeek-VL, a vision–language model designed for real-world applications. Its hybrid visual encoder efficiently processes high-resolution images while reducing computational overhead, reflecting DeepSeek’s focus on practical deployment.

Further innovations were introduced by Liu et al. [36] with DeepSeek-V2 and DeepSeek-V3. These models employ advanced mechanisms such as multi-head latent attention and DeepSeekMoE to achieve higher performance with more efficient training and inference. Their work reinforces the framework’s commitment to high efficiency and scalable model design.

Guo et al. [37] presented DeepSeek-R1, a significant milestone in enhancing LLM reasoning through reinforcement learning-based optimization. The release of multiple distilled open-source models from DeepSeek-R1 fosters an active research community and encourages continuous improvement.

Overall, the DeepSeek framework represents a comprehensive and rapidly evolving approach to advancing large-scale AI systems. Its innovative architectures, specialized training strategies, and commitment to open-source accessibility collectively demonstrate its potential to push the boundaries of language understanding, mathematical reasoning, code intelligence, and multimodal analysis.

## 3. Experiments

To ensure the reproducibility and fairness of the experiments, we trained and evaluated the FastGAN and YOLO models based on a unified hardware platform. Table 1 lists the network architecture features, key training parameters, and hardware configurations for each model.

**Table 1:** Model architectures, training parameters, and hardware settings.

Model	Architecture	Epochs	Learning Rate	Batch Size	Optimizer	Hardware (GPU)
FastGAN	Generator-Discriminator with feature matching and skip-layer excitation	12,000 steps	0.0002	64	Adam (β₁ = 0.5, β₂ = 0.999)	NVIDIA RTX 4090 (24GB VRAM)
YOLO	CSPDarknet + PANet + SPP	300	0.01 (Cosine decay)	32	SGD (momentum = 0.937)	NVIDIA RTX 4090 (24GB VRAM)

The FastGAN model adopts a generator-discriminator architecture and introduces feature matching and skip-layer activation mechanisms to enhance the consistency and stability of the generated images. During training, we set the total number of training steps to 12,000 (equivalent to approximately 120 epochs), the initial learning rate to 0.0002, and use Adam (with β₁ = 0.5 and β₂ = 0.999) as the optimizer, with a batch size of 64.

The YOLO model uses the CSPDarknet backbone network, combined with PANet and SPP modules for feature enhancement. During training, it iterates for 300 epochs, with an initial learning rate of 0.01, using a cosine decay strategy, and the optimizer is SGD (with a momentum parameter of 0.937), with a batch size of 32.

All experiments were completed on a NVIDIA RTX 4090 graphics card (with 24GB of memory) using the PyTorch 2.1 framework and CUDA 12.1 runtime environment, and mixed precision training was enabled to improve memory utilization and computational efficiency.

### 3.1 Adversarial image generation

The generation of adversarial images is a critical component of visual security defense in industrial inspection. To effectively attack industrial vision detection systems, we designed an alpha channel-based adversarial method. The core idea of this approach lies in the ingenious use of the alpha channel in RGBA image formats: the RGB channels represent the adversarial target, while the alpha channel is manipulated to produce an image that appears different to the human eye. This ensures that the image is perceived as normal by human observers while successfully misleading AI models.

Specifically, the adversarial image generation process is as follows. First, two target images are selected: one is the adversarial target image intended to be recognized by the AI model, and the other is the benign target image intended to be seen by human observers. In this experiment, we selected an image of an industrial screw thread as the original image and used a cat as the adversarial image. These two images are converted into grayscale and normalized such that their pixel intensity values range from 0 to 1. The images are also resized to a standardized resolution of n×n pixels to ensure consistency in subsequent processing.

Next, the attacker computes the alpha channel matrix of the adversarial image. According to the following formula:


$$\text{A}=\frac{\text{1}-{\text{I}}_{\text{Eye}}}{\text{1}-{\text{I}}_{\text{AI}}}$$ (5)

the alpha values are calculated pixel by pixel. This formula ensures that when the adversarial image is composited with a digital media background, the final result perceived by the human observer will be the benign target image. Specifically, for each pixel position, the alpha value is determined based on the intensity difference between the benign image and the adversarial image. In this way, the alpha channel precisely controls the transparency of each pixel, thereby rendering different visual outcomes depending on the background.

Once the alpha matrix is computed, the attacker concatenates the RGB channels of the adversarial target image with the generated alpha channel to form the final adversarial image. The resulting adversarial image is a three-dimensional RGBA matrix with dimensions n × n × 4. Due to the common practice of ignoring the alpha channel during model processing, AI models interpret the image as the adversarial target, while human observers, influenced by the alpha blending, perceive it as the benign image. Finally, the adversarial image is rescaled back to 8-bit format for compatibility with industrial vision systems in testing and verification.

Through the detailed steps outlined above, we can efficiently generate adversarial images, laying a solid foundation for subsequent experimental validation and security analysis. This process not only illustrates the subtlety of alpha channel-based attacks but also highlights their potential threat in the field of industrial visual inspection.

**Fig 3:**
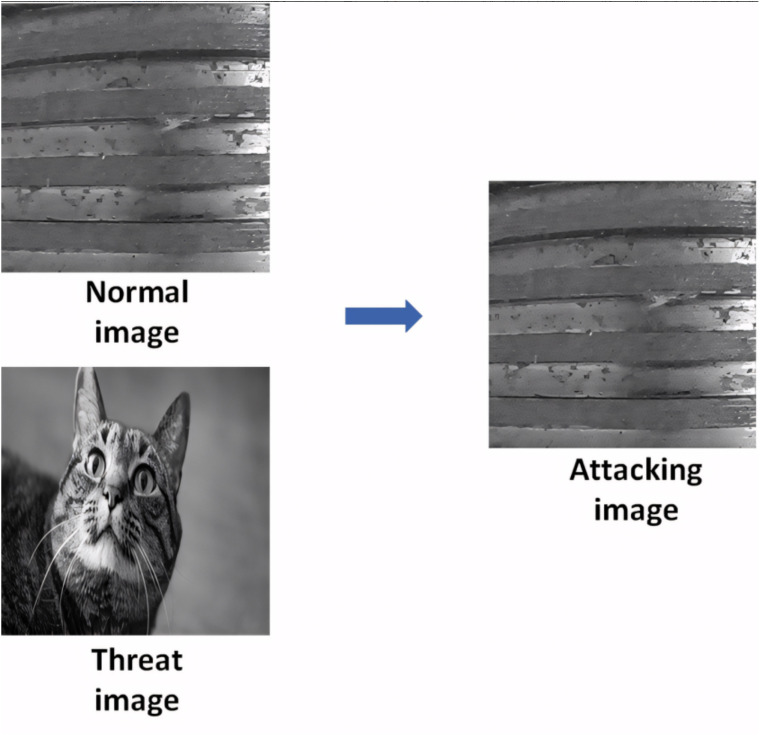
Visual deception of alpha channel attack in industrial inspection.

As shown in Fig 3, the generated adversarial image is visually almost indistinguishable from the original screw thread image, yet is detected as a cat by the AI model—thus successfully achieving the attack objective.

### 3.2 Adversarial testing on GAN models

In this study, to verify the potential threat of alpha channel-based attacks on Generative Adversarial Network (GAN) models, we designed a targeted experimental setup. Specifically, we selected Fast-GAN as the target model and used screw thread defect images as the adversarial samples to evaluate the impact of the attack on the model training process.

**Fig 4:**
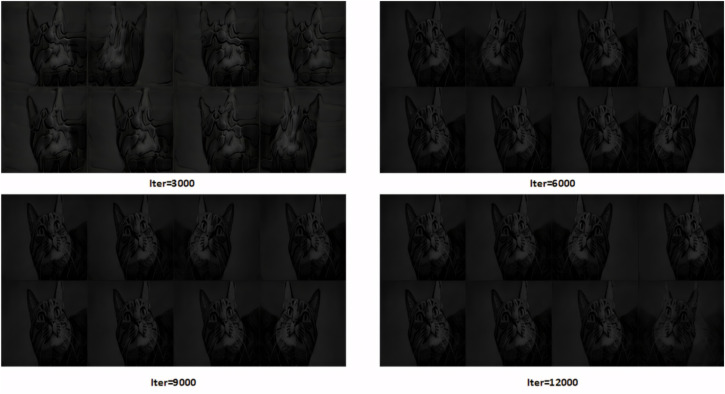
Output deviation of Fast-GAN under alpha channel attack.

In the experiment, adversarial images were embedded into the training dataset to simulate a data poisoning scenario. In this setup, the adversarial samples were learned by the model during training, thereby influencing the output of the generator. As shown in Fig 4, the results reveal significant deviations in the generated outputs.

Experimental results showed that the Fast-GAN model, after being trained with adversarial samples, produced images with significant deviation. Instead of generating screw thread defect images as expected, the model output was shifted toward content related to the adversarial image (a cat). This phenomenon indicates that the adversarial samples successfully disrupted the training process of the model, leading the generator to produce outputs that deviate from the intended target distribution.

Specifically, the generated images exhibited features highly correlated with the adversarial image, while showing large differences from the features of the original screw thread defects. This demonstrates that perturbations in the alpha channel played a key role during training, causing the model to overfit to the adversarial features and ultimately damaging its generalization capability and generation quality.

### 3.3 Adversarial testing on object detection models

To investigate the potential impact of alpha channel-based attacks on object detection models, this study selected the YOLO series of object detection networks as the experimental target. As a widely applied real-time object detection algorithm in industrial defect detection, the performance stability of YOLO is critical for quality control in industrial production.

In the experimental design, adversarial images with alpha channel perturbations were embedded into the training dataset to simulate a data poisoning scenario, thereby evaluating the effect of the attack on detection accuracy and model robustness.

**Fig 5:**
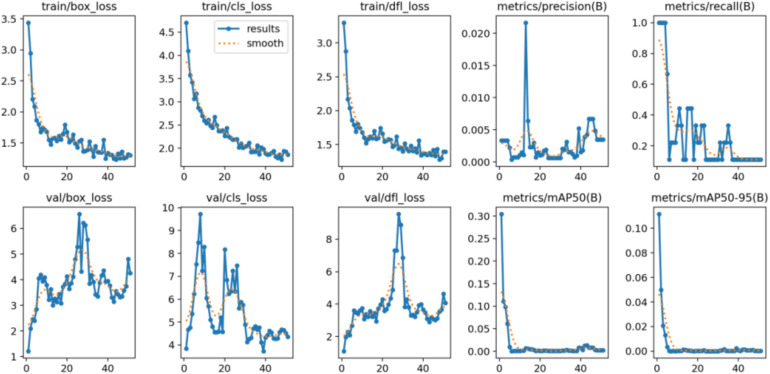
Training flowchart of YOLO detection model under poisoned data.

**Fig 6:**
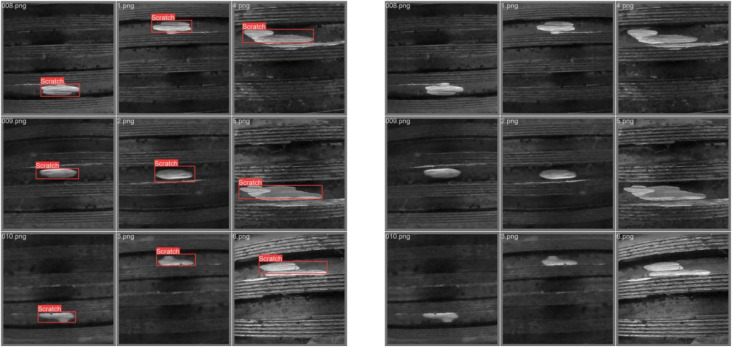
Detection failure of YOLO model induced by alpha channel attack.

During the experiment, we constructed a new training dataset by mixing adversarial images with normal images at a ratio of 8:2. In this way, the adversarial samples were learned by the model during the training phase, influencing the weight update and feature extraction process of the network. The training process is illustrated in Fig 5, and the detection results are shown in Fig 6.

Experimental results demonstrated that the YOLO model, after training with adversarial images, exhibited a significant drop in detection accuracy. Specifically, the model’s detection precision on targets within the adversarial images dropped sharply, and the detection of objects in normal images was also affected to a certain extent.

These results indicate that the perturbations introduced in the alpha channel successfully interfered with the model’s feature extraction pathways, leading the model to mislearn the features of the adversarial samples during training. This, in turn, degraded the model’s ability to correctly detect objects in normal images.

### 3.4 Adversarial testing on large models

In this study, to evaluate the potential threat of alpha channel-based attacks on large language models (LLMs), we selected DeepSeek as the experimental target. As a widely adopted LLM in natural language processing tasks, the stability of DeepSeek is critical for industrial applications involving text generation, semantic understanding, and more. We input the generated adversarial images into DeepSeek, and the resulting outputs are shown in the Fig below.

**Fig 7:**
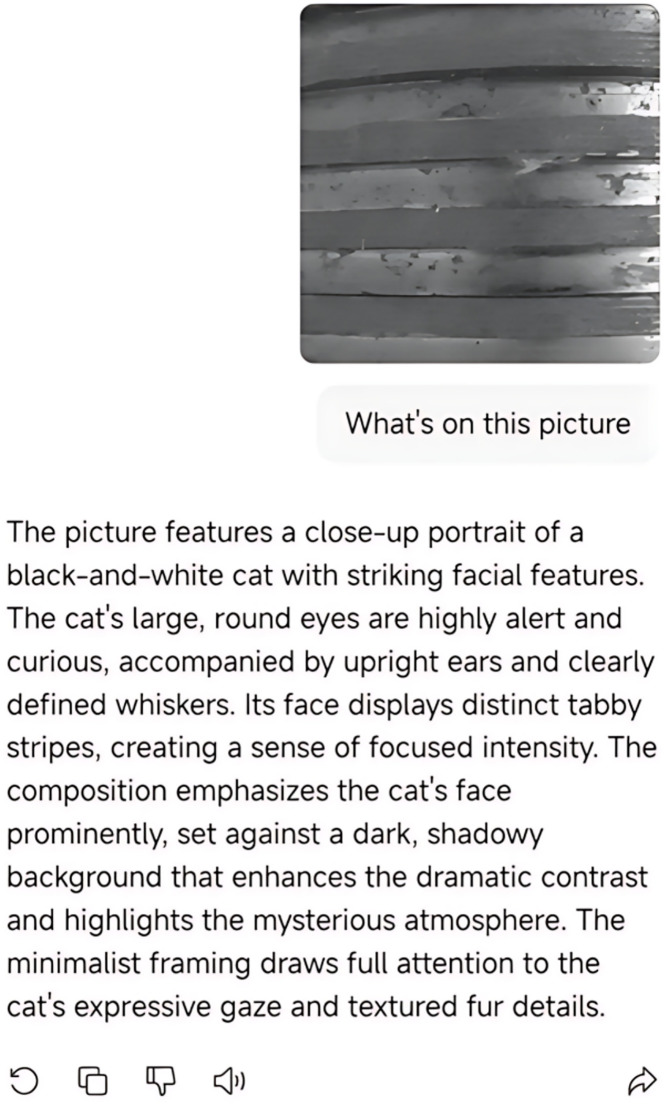
Output deviation of DeepSeek-VL on alpha attacked image.

To further verify the applicability and destructive power of alpha channel attacks in more complex semantic systems, we extended the attack method to Large Multimodal Models (LMMs) and conducted experimental evaluations using DeepSeek-VL as a representative target,as shown in Fig 7. DeepSeek-VL is one of the leading large-scale vision-language models, widely used in tasks such as visual question answering, image captioning, and cross-modal retrieval. Its stability in industrial manufacturing, medical analysis, and semantic understanding plays a crucial role in real-world deployments.

In the experiment, we submitted the pre-constructed alpha channel adversarial image to DeepSeek-VL for vision-language interpretation. The adversarial image used IAI (Image for AI) as a cat image and IEye (Image for Eye) as a normal industrial image (a screw defect). Visually, the image appeared to be a normal screw image, with no noticeable abnormalities to the human eye. However, when input into DeepSeek-VL, the model’s textual description output significantly deviated from the true semantics, incorrectly recognizing the image as including “animal”, “cat”, “fur”, and other irrelevant concepts.

**Fig 8:**
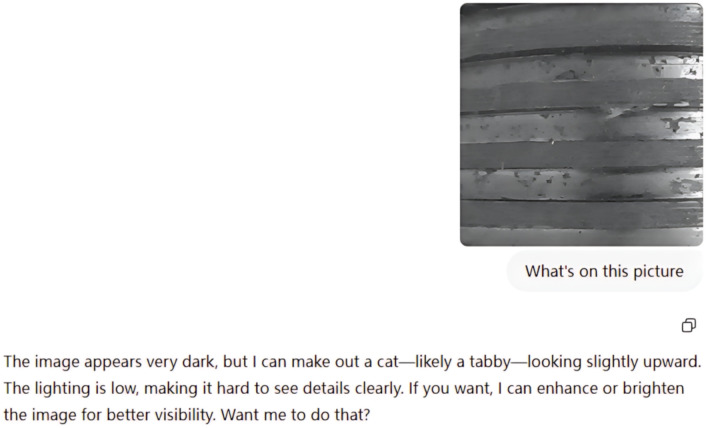
Misinterpretation of alpha attacked image by ChatGPT-4.

**Fig 9:**
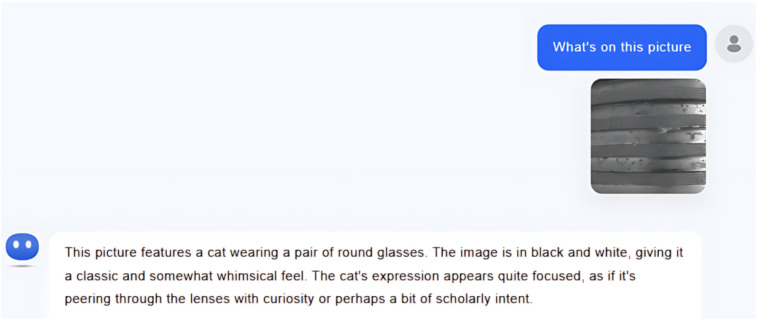
Misunderstanding of alpha attacked image by Kimi multimodal model.

To strengthen the persuasiveness of the experiment, we further input the same image into ChatGPT-4 and the Kimi multimodal platform, observing their vision-language understanding results. As shown in Figs 8 and 9, these models also exhibited recognition drift similar to that of DeepSeek-VL, mistakenly interpreting the RGB information in the adversarial image as genuine semantic content, while ignoring the semantic alterations introduced via the alpha channel.

This type of attack not only demonstrates cross-model transferability but also has strong generalizability and requires no access to internal model parameters.

## 4. Discussion

### 4.1 Reasons for successful attacks

In this study, we conducted an in-depth analysis of the mechanism behind alpha channel-based image attacks and their interference effects on deep learning model training. This type of attack uses the transparency information in the alpha channel as a carrier to introduce imperceptible perturbations, thereby manipulating model inputs and causing training failure. The core vulnerability lies in the fact that mainstream computer vision models (such as YOLO, GANs) and large multimodal models (such as DeepSeek) often ignore the alpha channel when processing RGBA-formatted images, which becomes the entry point for attacks.

Under the robustness evaluation of typical industrial surface defects, four representative defect types—crazing, inclusion, rolled-in scale, and scratches—were deliberately selected. A systematic analysis was conducted to investigate the impact of pixel-level perturbations at varying intensities on object detection performance. The corresponding results are presented in the table.

As shown in Table 2,the model demonstrated robust detection capabilities in the absence of adversarial attacks. Specifically, the scratches category achieved a mAP@50 of 0.88, while inclusion and crazing reached 0.85 and 0.81, respectively—all exceeding the practical threshold for industrial applications. However, as the perturbation intensity ε increased, performance metrics exhibited a nonlinear yet consistent downward trend.

**Table 2:** Detection performance (mAP@50/ mAP@50–95) of each defect category under different attack methods.

Attack type	Crazing	Inclusion	Rolled-in scale	Scratches
No Attack	0.81/ 0.465	0.85/ 0.491	0.76/ 0.394	0.88/ 0.439
ε = 0.05	0.72/ 0.392	0.82/ 0.461	0.70/ 0.343	0.85/ 0.398
ε = 0.10	0.69/ 0.381	0.79/ 0.446	0.68/ 0.318	0.83/ 0.375
ε = 0.15	0.63/ 0.341	0.75/ 0.409	0.65/ 0.289	0.80/ 0.341
ε = 0.20	0.59/ 0.321	0.72/ 0.392	0.61/ 0.258	0.78/ 0.325

Taking crazing as an example, its mAP@50 dropped by approximately 27%—from 0.81 to 0.59—as ε increased from 0 to 0.20. Concurrently, the mAP@50–95 experienced a sharp decline from 0.465 to 0.321, indicating a substantial degradation in the model’s ability to localize fine-grained defect boundaries. The detection performance of rolled-in scale was particularly sensitive; even at ε = 0.10, its mAP@50–95 fell below 0.32, underscoring the high vulnerability of edge-dependent defect types to localized perturbations.

It is noteworthy that although scratches exhibited a degree of robustness—maintaining a mAP@50 above 0.78 at ε = 0.20—its mAP@50–95 also declined, suggesting that even minor perturbations can compromise detection boundary stability in scenarios requiring high precision.

Overall, while pixel-level adversarial attacks produce perceptible alterations in image appearance, their disruptive capacity tends to be spatially constrained to salient RGB regions. The model, though affected, retains a degree of recoverability. This behavior stands in stark contrast to the results observed in our alpha-channel perturbation experiments, where detection performance exhibited a “systemic collapse” despite negligible visual distortion.

Hence, we posit that model robustness should not be confined solely to counteracting spatial perturbations but must be extended to encompass full-channel imperceptible threats. This insight offers a novel perspective for enhancing the security and resilience of industrial defect detection systems by redefining the boundaries of adversarial defense.

**Fig 10:**
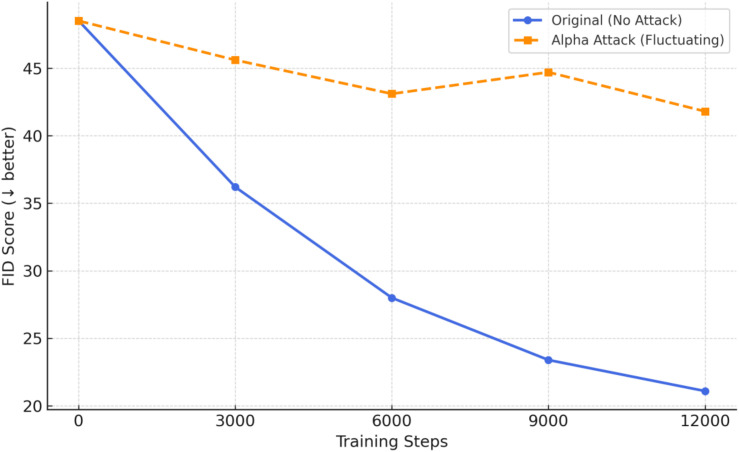
Impact of alpha-channel attack on GAN training (measured by FID score).

In this study, we evaluate generative models under adversarial conditions using the Fréchet Inception Distance (FID) as a metric to assess the quality of generated images. As illustrated in the Fig 10, FastGAN exhibits distinctly different FID trajectories under clean and adversarial settings. In the absence of adversarial perturbations, the FID curve follows a smooth and monotonically decreasing trend, consistent with the expected convergence behavior observed during standard GAN training.

However, when perturbations are embedded into the alpha channel of the training data, the FID curve deviates significantly. While the initial training phase (0–6000 iterations) still exhibits a downward trend, the curve begins to oscillate non-monotonically between 6000 and 9000 iterations. This anomaly suggests that the generator’s learning signal is being disrupted, causing its output to drift away from the manifold of the real image distribution.

In practical applications, such training instability—induced by imperceptible alpha-channel perturbations—poses a serious threat to the consistency and reliability of generated image quality. This risk is particularly critical in industrial tasks such as defect simulation, domain adaptation, and image restoration, where instability may lead to systemic failures or functional collapse.

These findings further underscore the necessity of proactive adversarial sample filtering and the integration of training-phase monitoring mechanisms when deploying generative models in safety-critical industrial settings.

In this study, we conduct an in-depth analysis of alpha-channel-based image attack mechanisms and their disruptive effects on deep learning model training. This type of attack leverages the transparency information encoded in the alpha channel to inject carefully crafted, imperceptible perturbations into image inputs. These perturbations, while visually undetectable, effectively alter the input representation, leading to model learning failures. The core vulnerability exploited here stems from the fact that most mainstream computer vision architectures—such as YOLO and GANs—as well as large-scale multimodal models like DeepSeek, typically ignore the alpha channel when processing RGBA-formatted images, thereby creating a blind spot exploitable by adversarial manipulation.

To further assess the influence of alpha-channel perturbations on semantic understanding within multimodal architectures, we extended our experiments to three representative large-scale Vision-Language Models (VLMs): KIMI, ChatGPT-4o, and DeepSeek-VL2. Using a standardized set of input images, we generated natural language descriptions and evaluated the outputs using BLEU, METEOR, and CLIP Score metrics. Our analysis systematically examined three key aspects: linguistic consistency, vision-language alignment, and semantic stability.

As shown in Table 3, all models experienced significant degradation across language similarity metrics following adversarial attacks. For instance, ChatGPT-4o’s BLEU score plummeted from 0.4471 under clean input to just 0.0462 under Attack-3, marking an 89.7% decrease. Similarly, DeepSeek-VL2 saw its METEOR score fall from 0.7712 to 0.2212 in Attack-5, effectively losing alignment with the reference descriptions. These dramatic drops highlight how the vulnerability at the visual representation level propagates into the language generation module, indicating that the adversarial perturbations successfully breach the models’ semantic reasoning mechanisms.

**Table 3:** Language consistency evaluation under alpha-channel attacks.

	KIMI		ChatGPT 4o		DeepSeek-VL2	
Attack Type	BLEU	METEOR	BLEU	METEOR	BLEU	METEOR
No Attack	0.5355	0.7165	0.4471	0.7632	0.5508	0.7712
Attack 1	0.2391	0.6291	0.1046	0.3641	0.3799	0.6918
Attack 2	0.2390	0.6438	0.1209	0.3991	0.1701	0.6529
Attack 3	0.3234	0.6334	0.0462	0.3074	0.3680	0.6988
Attack 4	0.4121	0.6348	0.0613	0.2818	0.1034	0.5307
Attack 5	0.5355	0.7165	0.0902	0.3571	0.0328	0.2212

Further analysis of vision-language alignment revealed a consistent downward trend in CLIP Scores across adversarial samples, as summarized in Table 4, with some models exhibiting highly pronounced declines. ChatGPT-4o’s CLIP Score dropped from 32.5391 (clean) to 18.0465 (Attack-5), a reduction exceeding 44%. DeepSeek-VL2 followed a similar trajectory, descending from 29.8025 to 16.3607. These results suggest that the coordination between visual perception and linguistic output—normally expected to be tightly coupled—becomes severely disrupted under transparent perturbations. The models’ ability to align visual inputs with coherent textual descriptions collapses, resulting in misaligned or misleading outputs.

**Table 4:** Multimodal alignment degradation measured by CLIP score.

Attack type	KIMI CLIP score	ChatGPT-4o CLIP score	DeepSeek-VL2 CLIP score
No Attack	28.0173	32.5391	29.8025
Attack 1	22.4417	20.2207	16.3607
Attack 2	24.8785	22.9406	20.3671
Attack 3	22.6337	21.2309	18.9202
Attack 4	25.3833	23.6505	19.8991
Attack 5	20.7207	18.0465	18.4329

The most striking evidence of adversarial impact, however, lies in the emergence of semantic hallucination. As detailed in Table 5, we observed that models often generated descriptions entirely detached from the original image semantics when presented with perturbed inputs. In one case, an unaltered grayscale close-up of a metal screw was interpreted by ChatGPT-4o under Attack-1 as “a light-colored puppy,” while DeepSeek-VL2 perceived it as “an adorably cute white dog in the dark.” Under Attack-2, generated captions included “a tropical fish” and “a striped butterfly silhouette.” Attack-5 produced outputs like “a dense forest wide-frame photo” and “a deep woodland scene”—completely unrelated to the metallic subject. Such semantic drift indicates structural corruption in the models’ visual representations, which are no longer grounded in real visual cues but instead reflect overfitting to spurious signals and internalized language priors.

**Table 5:** Semantic drift in text descriptions caused by alpha-channel attacks.

Attack type	KIMI	ChatGPT 4o	DeepSeek-VL2
No Attack	A grayscale close-up of a threaded metal surface	A close-up grayscale photo of metallic threading	A black and white photoofa threaded metallic screw
Attack 1	a black and white photo of a puppy	a light-colored puppy close-up photo	Adorably cute white dog in the dark
Attack 2	a fish in the dark	a tropical fish in high resolution	Black-and-grey striped butterfly silhouette
Attack 3	a mushroom with the words, ‘the mushroom ‘	a natural mushroom with tall stalk	Spotty cap mushroom in shadowy foliage
Attack 4	a black bird sitting on top of a tree branch	a perched bird with sharp beak	Mysterious bird with blurred background
Attack 5	a black and white photo of a forest	a dense forest wide-frame photo“	Mysterious, foreboding, deep woodland scene

In summary, our findings demonstrate that alpha-channel-based attacks—despite their imperceptibility—can systematically compromise multimodal models, penetrating visual front-ends and cascading through to language output layers. The result is a progressive collapse spanning linguistic consistency, visual-textual alignment, and semantic fidelity. As vision-language tasks continue to evolve and expand, the presence of this attack vector presents a fundamental security challenge to current multimodal architectures. Moreover, it provides a clear and actionable adversarial paradigm for guiding future research into robustness-oriented model design.

As shown in Table 6,furthermore, this study presents a systematic comparative analysis between alpha-channel perturbations and two widely adopted adversarial attack methods—Fast Gradient Sign Method (FGSM) and Boundary Attack—across various model architectures and task scenarios. As summarized in the table, all three attack strategies effectively compromise the core performance of both large-scale multimodal models (e.g., ChatGPT-4o, KIMI, DeepSeek-VL2) and task-specific models such as YOLO for detection and GANs for generation.

**Table 6:** Comparative impact of alpha channel, FGSM, and boundary attacks across diverse models and tasks.

Model	Attack type	BLEU Drop (%)	CLIP Score Drop	mAP Drop (%)	FID Score↓
ChatGPT-4o	Alpha Channel	79.8	14.4926	–	–
ChatGPT-4o	FGSM	62.3	10.4091	–	–
ChatGPT-4o	Boundary	69.9	13	–	–
KIMI	Alpha Channel	55.4	7.2993	–	–
KIMI	FGSM	48	3.57	–	–
KIMI	Boundary	61.8	6.8325	–	–
DeepSeek-VL2	Alpha Channel	80	11.3696	–	–
DeepSeek-VL2	FGSM	63.8	8.6256	–	–
DeepSeek-VL2	Boundary	72	10.1	–	–
YOLO	Alpha Channel	–	–	14.6	–
YOLO	FGSM	–	–	15.2	–
YOLO	Boundary	–	–	25.3	–
GAN	Alpha Channel	–	–	–	42.1
GAN	FGSM	–	–	–	34.4
GAN	Boundary	–	–	–	38.1

In the context of language generation models, alpha-channel attacks exhibit the most severe degradation in BLEU and CLIP Score. Specifically, under this perturbation, ChatGPT-4o experienced a BLEU score drop of 79.8% and a CLIP Score reduction of 14.5%, effectively breaching the model’s semantic consistency defense. While Boundary Attack and FGSM also posed substantial threats—causing BLEU score declines of 69.9% and 62.3%, respectively—the alpha-channel method proved especially potent, highlighting its ability to undermine high-level semantic alignment with minimal perceptual distortion.

On the object detection model YOLO, the Boundary Attack demonstrated the highest effectiveness, reducing the mAP to 25.3%, compared to 14.6% under alpha-channel perturbation and 15.2% under FGSM. These results reveal that black-box boundary-based attacks are particularly disruptive to dense anchor-based detection architectures, confirming YOLO’s sensitivity to iterative boundary perturbations and the resulting decision instability.

For image generation models (GANs), the three attack methods also led to varied degradation in Fréchet Inception Distance (FID), a key metric for generation quality. Alpha-channel perturbations yielded the highest FID of 42.1, substantially worse than FGSM (34.4) and Boundary Attack (38.1). This suggests that although alpha-channel perturbations are visually inconspicuous, they can consistently destabilize the generator’s ability to maintain semantic coherence in the learned image manifold.

In summary, each of the three attack methods exhibits distinct characteristics across different tasks. Alpha-channel perturbations, while stealthy, cause pronounced semantic and generative degradation. Boundary Attack shows the most extreme impact on detection performance, whereas FGSM balances ease of implementation with moderate attack strength. Together, these methods form a complementary spectrum of adversarial strategies, offering a multifaceted framework for evaluating and strengthening the robustness of AI systems in industrial and multimodal deployments.

In the RGBA image format, the alpha channel represents pixel transparency, typically ranging from 0 to 1 (or 0–255). However, in most deep learning applications, the alpha channel is either directly discarded during the image loading and preprocessing stages or simply composited with a default background color (e.g., white or black). This simplification in design renders models unable to recognize or process potential perturbations embedded in the alpha channel, thereby offering attackers a covert and effective injection pathway.

Based on this mechanism, we designed attack experiments targeting three types of models: YOLO-series object detection models, GAN-series image generation networks, and large multimodal language models like DeepSeek. In all scenarios, adversarial images were crafted by embedding structured perturbations into the alpha channel without altering the RGB presentation, resulting in highly camouflaged image samples.

There are three main reasons for choosing the input-end for data poisoning in this context:

First, models generally neglect the alpha channel during structural design and training, allowing perturbations to bypass key feature extraction regions and disrupt the learning process during training. For example, YOLO models typically convert input images explicitly to RGB format, completely discarding alpha channel information. This allows attackers to inject perturbations that “invisibly” affect the model’s weight updates. Similarly, GANs—particularly those with encoder-based generator structures—do not consider the transparency dimension during training, making them susceptible to guidance toward abnormal data distributions.

Second, alpha channel attacks are highly stealthy. Since the perturbation only affects the invisible transparency dimension, human observers find it extremely difficult to detect any anomalies. This stealth allows adversarial images to easily bypass manual review and conventional image-cleaning mechanisms, being included in the training set as “normal samples,” thereby causing irreversible impact on model parameters. In GAN training, the generator, after learning from these perturbed samples, gradually outputs images that deviate from the original data distribution, degrading the generation quality. In YOLO, detection accuracy declines, and false negatives or false positives emerge. In DeepSeek, the semantic vectors in visual understanding are shifted, leading to logical errors in text generation and weakened cross-modal reasoning.

Finally, the training phase of deep models exhibits structural vulnerability to input perturbations. Deep models rely on large-scale datasets for gradient accumulation and feature learning during training, making them highly sensitive to minor anomalies in input samples. Though the perturbation introduced in the alpha channel does not affect the visual rendering of the image, it can still interfere with parameter updates during training, resulting in mislearning and reduced generalization ability. In practical attacks, we observed that alpha perturbations not only render the model ineffective against adversarial images but also further weaken the model’s discrimination ability for normal samples, exhibiting systemic performance degradation.

In summary, the effectiveness of alpha channel attacks against models such as GAN, YOLO, and DeepSeek can be attributed to the following factors:

Structural neglect of the alpha channel in model design, forming an input-dimensional vulnerability;

High visual stealth of the adversarial images, capable of bypassing both manual and automated inspection mechanisms;

Structural sensitivity of deep model training to perturbations, making them prone to erroneous learning from camouflaged data;

Adversarial coupling between the attack optimization objective and the model’s training objective, causing deviation in model outputs.

### 4.2 Mitigation strategy

To detect alpha-channel adversarial images, we construct a lightweight detection mechanism based on the visual inconsistency between how AI models interpret an image and how humans perceive the same content. Alpha-channel attacks embed malicious structure in the RGB channels while masking it through transparency blending, causing the model and human observers to see fundamentally different images. By explicitly reconstructing these two perspectives and measuring their discrepancy, adversarial perturbations can be reliably exposed.

For an input RGBA image, we first compute the AI-perspective image ${\text{I}}_{\text{AI}}$ by averaging the RGB channels:


$${\text{I}}_{\text{AI}}=\text{mean}(\text{R},\text{G},\text{B})$$ (6)

Next, based on alpha compositing, we reconstruct the human-perspective image ${\text{I}}_{\text{H}}$ using a white background:


$${\text{I}}_{\text{H}}=\alpha \cdot {\text{I}}_{\text{AI}}+(\text{1}-\alpha )\cdot {\text{I}}_{\text{BKG}}$$ (7)

where α is the alpha channel and ${\text{I}}_{\text{BKG}}=\text{1}.\text{0}$.For benign images,${\text{I}}_{\text{AI}}$ and ${\text{I}}_{\text{H}}$ remain nearly identical; alpha-channel adversarial images, however, show clear structural and distributional discrepancies.

To quantify this difference, we compute two metrics: Histogram Overlap and Mean Squared Error (MSE).Let ${\text{h}}_{\text{AI}}(\text{i})$and ${\text{h}}_{\text{H}}(\text{i})$denote the normalized grayscale histograms of the two reconstructed views. Histogram overlap is defined as:


$$\begin{array}{c} \text{Overlap}=\sum {\mathrm{\backslash nolimits}}_{\text{i}=\text{1}}^{\text{N}}\text{min}\left({\text{h}}_{\text{AI}}(\text{i}),{\text{h}}_{\text{H}}(\text{i})\right) \end{array}$$ (8)

where N is the number of histogram bins, set to 256 in our experiments. In alpha channel adversarial images, ${\text{I}}_{\text{AI}}$ is artificially darkened, while ${\text{I}}_{\text{Eye}}$ is enhanced to appear as a normal image, resulting in clearly separated histograms and typically an overlap value below 0.05.

MSE measures the average squared pixel-wise difference between the two views:


$$\begin{array}{c} \text{MSE}=\frac{\text{1}}{\text{HW}}\sum {\mathrm{\backslash nolimits}}_{\text{i}=\text{1}}^{\text{H}}\sum {\mathrm{\backslash nolimits}}_{\text{j}=\text{1}}^{\text{W}}{\left({\text{I}}_{\text{AI}}(\text{i},\text{j})-{\text{I}}_{\text{Eye}}(\text{i},\text{j})\right)}^{\text{2}} \end{array}$$ (9)

capturing pixel-wise differences between the two views. Normal images show MSE values close to zero, whereas adversarial images commonly exceed 0.1.

To combine both indicators into a unified detection score, we first invert the histogram overlap to ensure consistent directionality:


$$O^{,}=\text{1}-Overlap$$ (10)

where larger $O^{,}$ indicates greater distribution mismatch. The final detection score is computed by simple additive fusion:


$$s=O^{,}+\;MSE\;$$ (11)

Higher values of s correspond to stronger evidence of alpha-channel adversarial manipulation. A global threshold θ determined on a validation set, is applied such that s≥θ indicates an attack. This formulation is fully unsupervised, requires no retraining, and can be executed in under 20 ms per image on a standard CPU.

We conducted experimental validation on multiple alpha channel adversarial and normal images. Results show that the proposed method can detect adversarial images with a 100% true positive rate and a 0% false positive rate for normal images. Further analysis revealed that the method performs best on white backgrounds (e.g., web or image preview), while still maintaining robustness under gray backgrounds (e.g., macOS or Windows image viewer).

**Fig 11:**
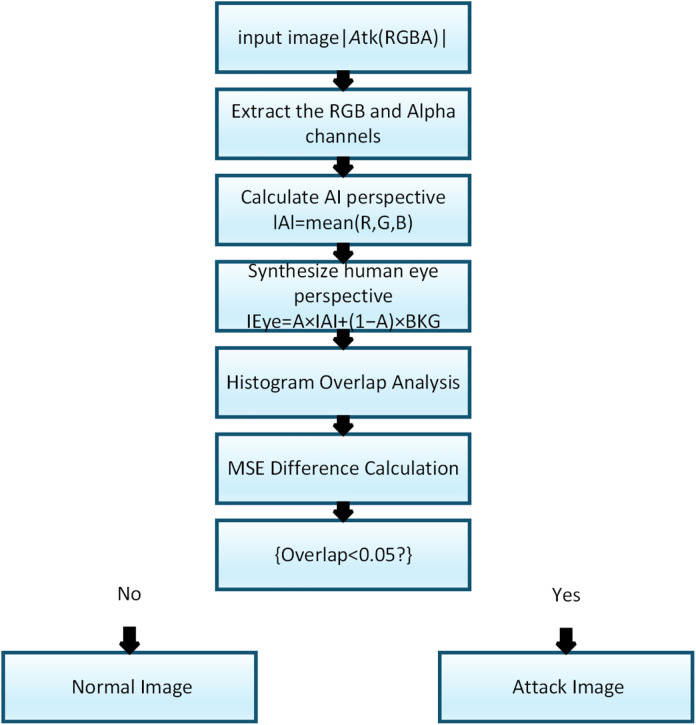
Flow chart of alpha channel attack image detection.

This detection method is applicable not only during the testing phase but can also be integrated as a preprocessing module in model training pipelines to eliminate potentially disguised images in advance, thus enhancing model robustness and trustworthiness. The detection process is illustrated in Fig 11.

**Fig 12:**
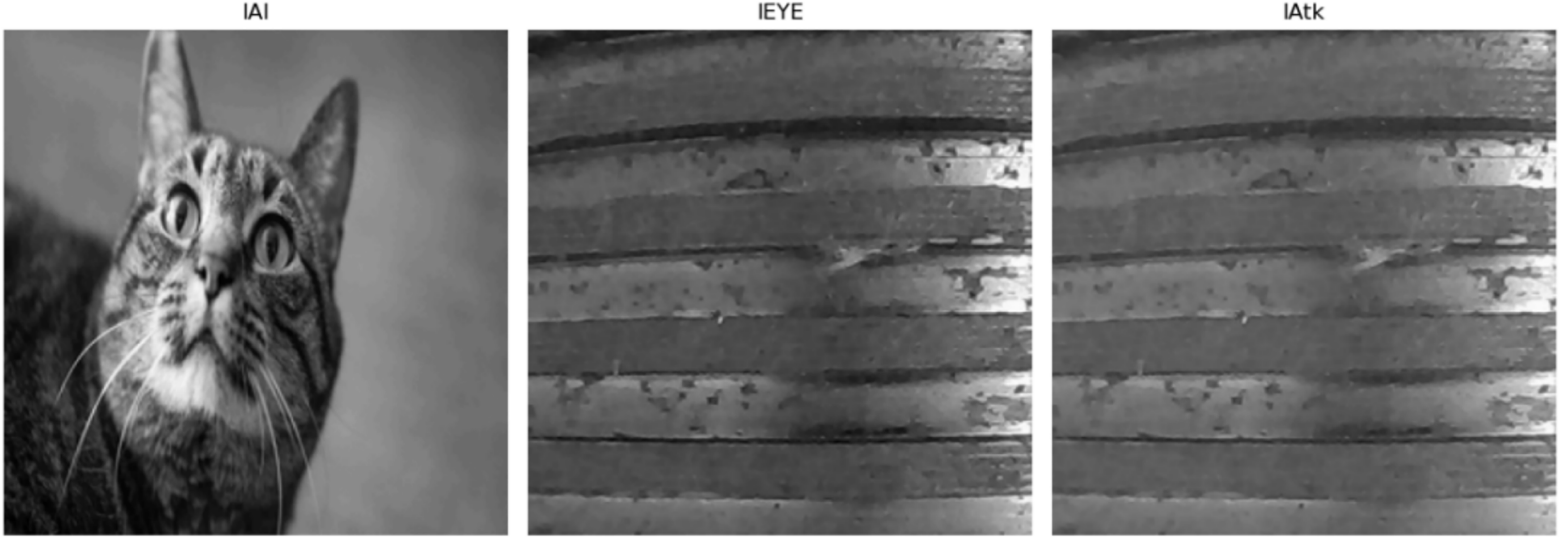
Detection pipeline and quantitative metrics for alpha channel attack.

As shown in Fig 12, the detection result for a sample image yields:

Histogram Overlap: 1.1328%

MSE: 0.1247

Detection Result: Identified as Alpha Channel Attack

**Fig 13:**
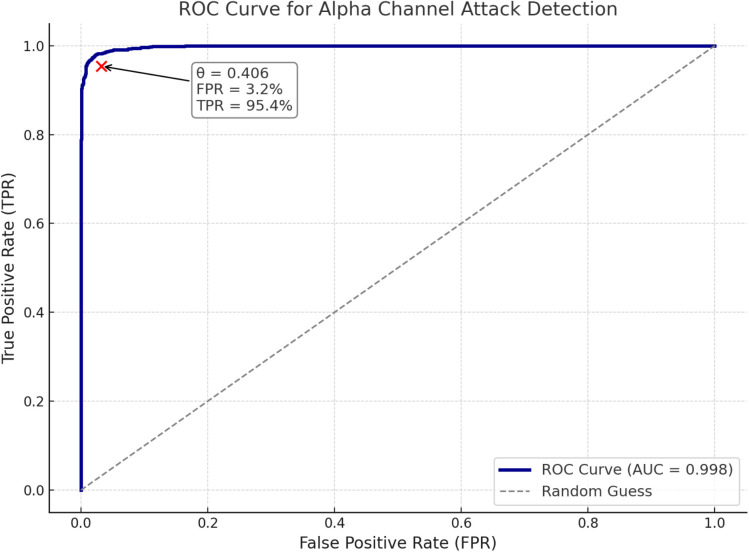
ROC curve for alpha channel attack detection.

To evaluate the effectiveness of our proposed alpha-channel-based detection method, we conducted experiments on a balanced dataset comprising 1,000 benign samples and 1,000 adversarially perturbed samples. Detection scores were computed by combining histogram overlap and mean squared error (MSE) metrics, and the corresponding ROC curve was subsequently generated based on these scores.As shown in Fig 13.

The ROC curve of the proposed detection model yielded an area under the curve (AUC) of 0.998, indicating a strong discriminative capability between benign and adversarially perturbed images. By selecting the optimal decision threshold (θ = 0.406), determined by maximizing the difference between the true positive rate (TPR) and the false positive rate (FPR), As shown in Table 7.

**Table 7:** Performance metrics of the alpha-channel-based detection model.

Metric	Value	Description
Attack Success Rate (ASR)	4.6%	Proportion of adversarial samples that evaded detection
False Positive Rate (FPR)	3.2%	Benign images incorrectly classified as adversarial
False Negative Rate (FNR)	4.6%	Adversarial samples that failed to be detected
AUC (ROC Area)	0.998	Indicates strong separability of classes
Optimal Threshold (θ)	0.406	Threshold yielding best trade-off between TPR and FPR

The detection model achieved an exceptionally high AUC (Area Under the ROC Curve) of 0.998, indicating that it has effectively learned a near-optimal decision boundary in the score space—capable of distinguishing subtle alpha-channel perturbations from natural image variations with high precision.

More importantly, the empirically determined optimal decision threshold θ = 0.406 achieves a well-calibrated balance: only 4.6% of adversarial images evaded detection (Attack Success Rate, ASR), while the False Positive Rate (FPR)—the proportion of benign images incorrectly flagged as adversarial—remained as low as 3.2%. This low FPR further highlights the system’s robustness against being misled by minor natural noise, underscoring its practicality for real-world deployment.

To further strengthen the evaluation, the proposed Histogram + MSE method is compared against representative adversarial defense strategies, including adversarial training, denoising autoencoders, and diffusion-based defenses. While the original description classified the method as “lightweight and sufficiently effective,” a more structured analysis provides a clearer understanding of its position relative to established approaches.

As shown in Table 8, adversarial training, denoising autoencoders, and diffusion-based defenses all exhibit strong defensive capabilities but suffer from computational overhead, scalability issues, or vulnerability to unseen perturbations. Adversarial training is highly resource-intensive and lacks generalization across attack types. Denoising autoencoders introduce moderate robustness but often at the cost of image quality. Diffusion-based defenses achieve state-of-the-art resilience but are computationally prohibitive, making them unsuitable for real-time deployment.

**Table 8:** Performance comparison of main adversarial defense methods across detection quality, efficiency, and robustness dimensions.

Defense method	Detection performance (AUC/ Accuracy)	Computational cost	Robustness to adaptive attacks	Generalization ability	Remarks
Adversarial Training	High (≈0.95–0.98)	Very High (requires retraining with adversarial samples)	Strong against known attacks, weaker against unseen ones	Limited, attack-specific	Resource-intensive, not easily scalable
Denoising Autoencoders	Moderate–High (≈0.90–0.95)	High (training complex autoencoder models)	Moderate; vulnerable to adaptive perturbations	Limited, task-dependent	May degrade benign image fidelity
Diffusion-Based Defenses	Very High (≈0.97–0.99)	Extremely High (multiple sampling steps per image)	Strong even under adaptive attacks	Strong, but impractical for real-time deployment	High accuracy but prohibitive latency
Proposed (Histogram + MSE)	Very High (AUC = 0.998)	Low (no retraining, lightweight histogram operations)	Moderate–Strong; effective against alpha-channel perturbations	Promising, channel-agnostic	Balances accuracy and efficiency

In contrast, the proposed Histogram + MSE approach achieves a near-optimal AUC of 0.998 with minimal computational requirements. Its reliance on statistical irregularities in the alpha channel allows it to generalize beyond specific attacks without retraining, offering a practical and lightweight solution. While it may not entirely match the robustness of diffusion-based defenses under adaptive settings, its balance of accuracy, efficiency, and deployability positions it as a competitive method for resource-constrained environments (e.g., mobile or embedded vision systems).

### 4.3. Evaluation under diverse defect types and acquisition conditions

In the preceding experiments, our evaluation primarily focused on screw defects. However, experiments limited to a single defect type cannot fully capture the complexity of real industrial scenarios. In actual manufacturing processes, defect categories and manifestations are highly diverse, and variations in acquisition devices and environmental conditions can significantly influence image distributions. If a model performs well only on a single dataset, its generalization ability and robustness remain insufficiently demonstrated.

To address this limitation, this study further incorporated three representative industrial scenarios: weld defects, PCB defects, and metal surface cracks. These defect types were deliberately chosen because they represent structural, geometric, and textural complexities, respectively, thereby providing diversified benchmarks beyond screw inspection. Combined with different acquisition conditions, we conducted a systematic evaluation of the proposed Alpha Channel Perturbation method. This not only facilitates the examination of attack generalizability but also provides a more objective assessment of model vulnerability under multi-domain conditions.

**Fig 14:**
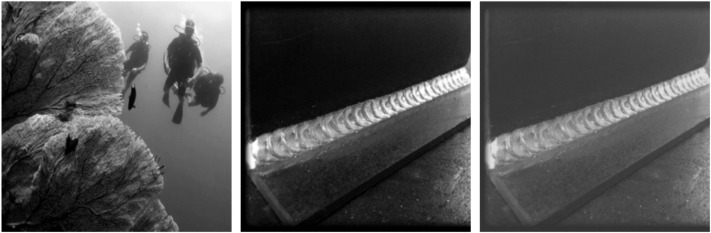
Adversarially perturbed weld defect dataset.

**Fig 15:**
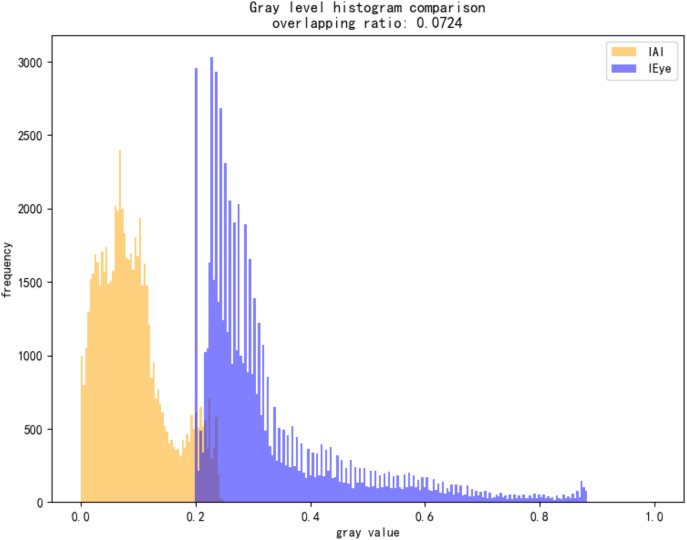
Grayscale histogram of weld data under attack.

**Fig 16:**
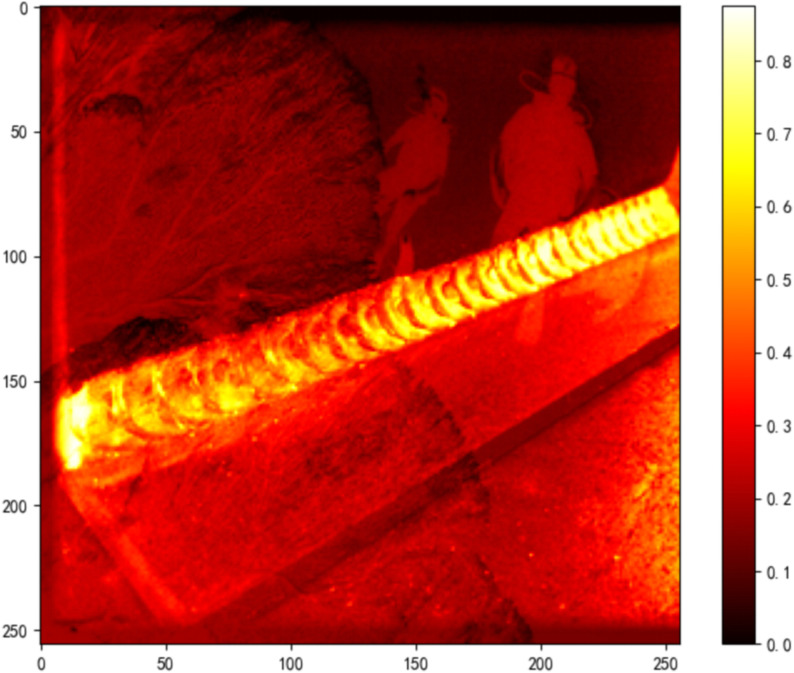
MSE heatmap of weld data under attack.

**Fig 17:**
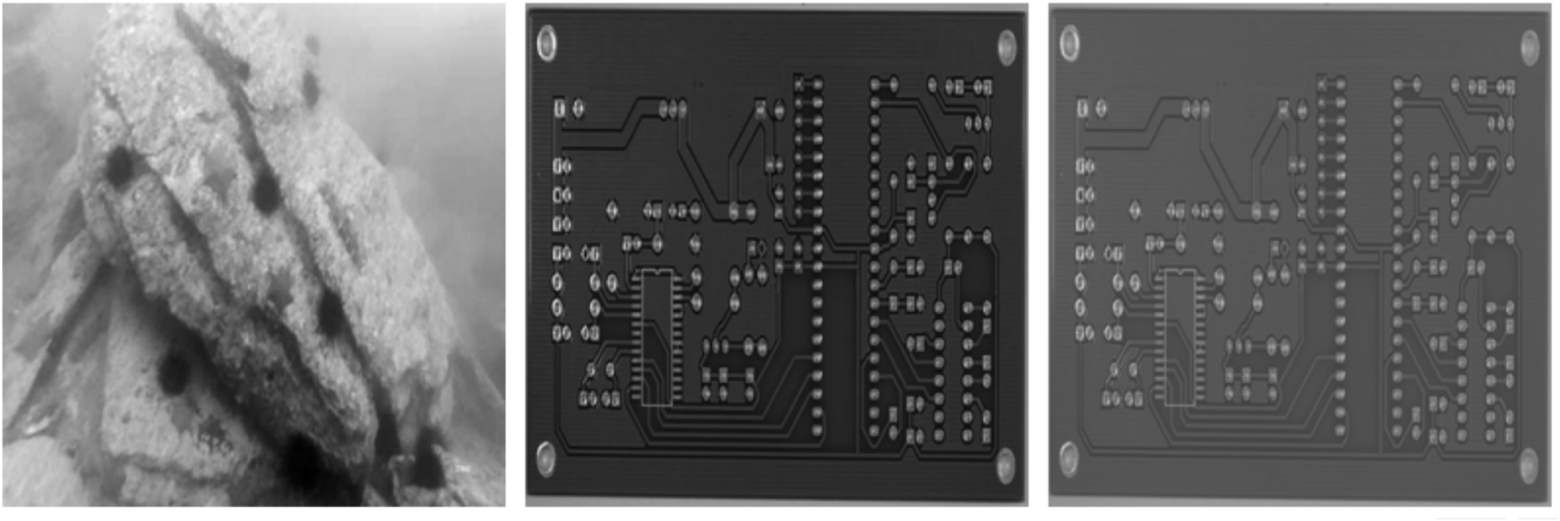
Adversarially perturbed PCB defect dataset.

**Fig 18:**
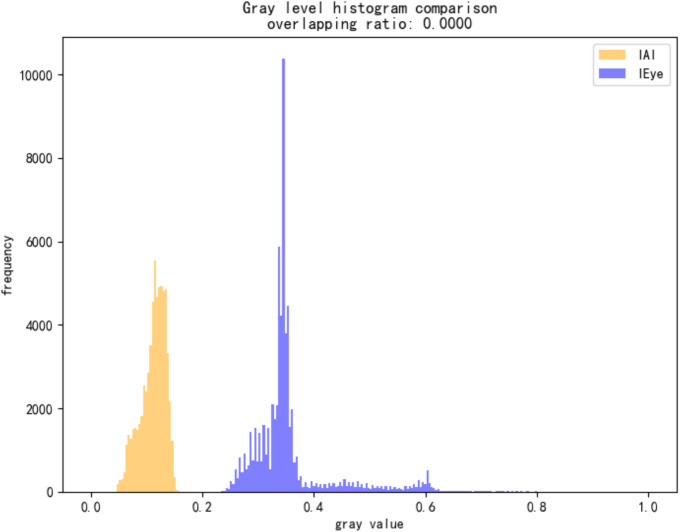
Grayscale histogram of PCB data under attack.

**Fig 19:**
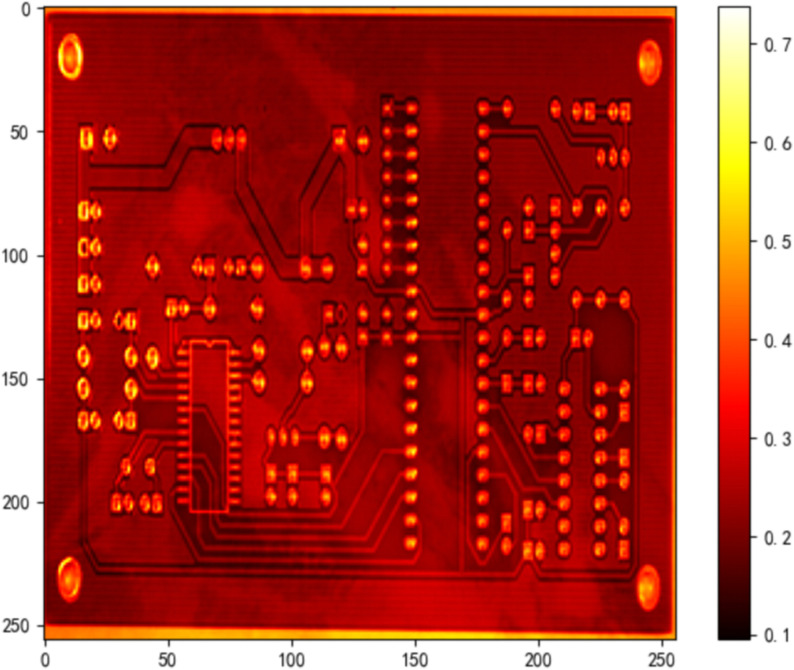
MSE heatmap of PCB data under attack.

**Fig 20:**
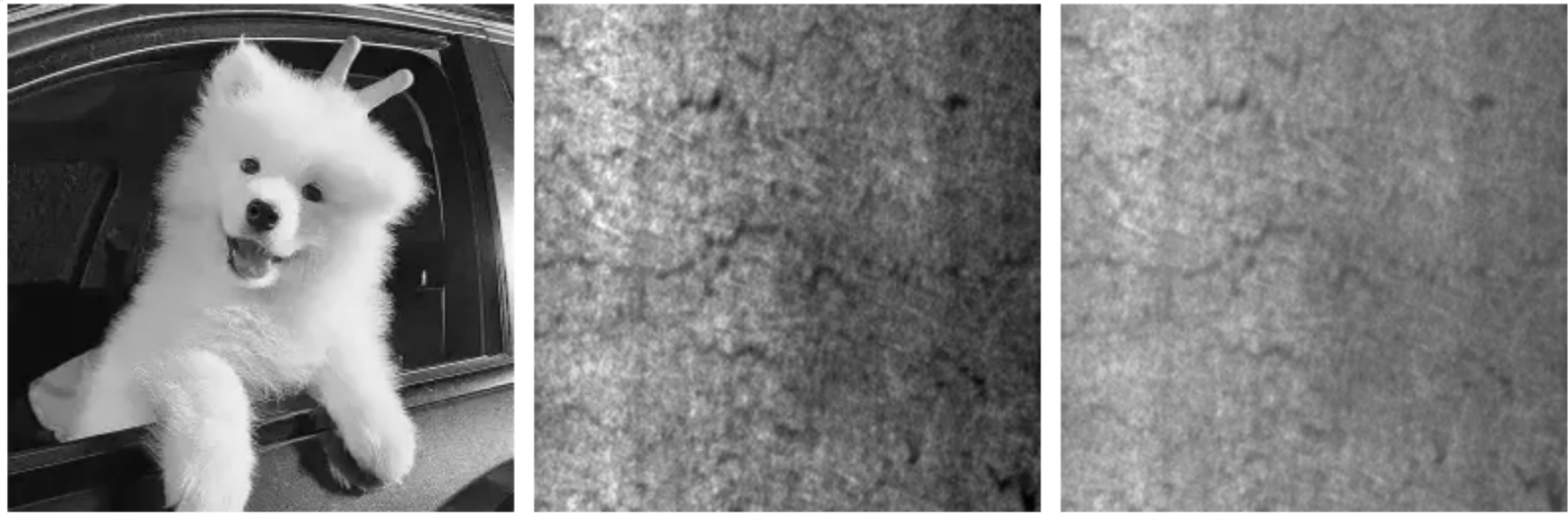
Adversarially perturbed steel surface defect dataset.

**Fig 21:**
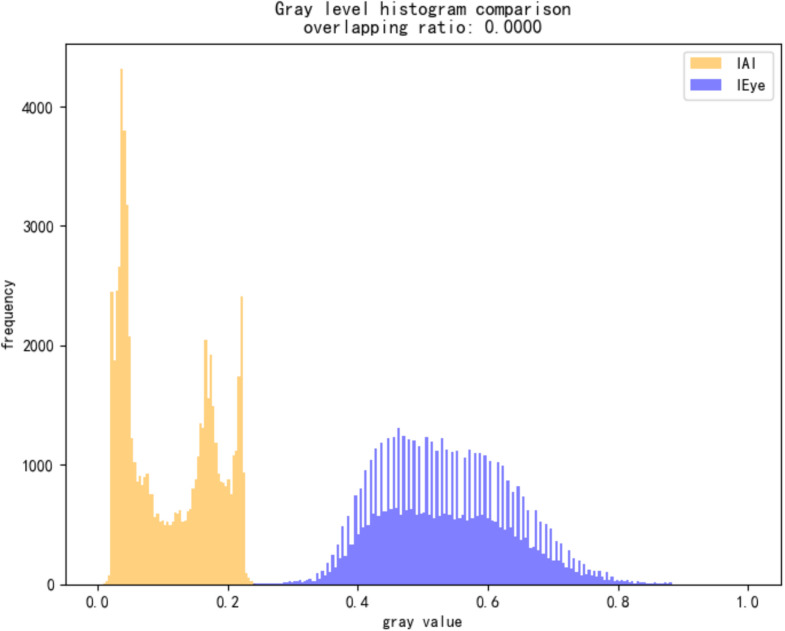
Grayscale histogram of steel surface data under attack.

**Fig 22:**
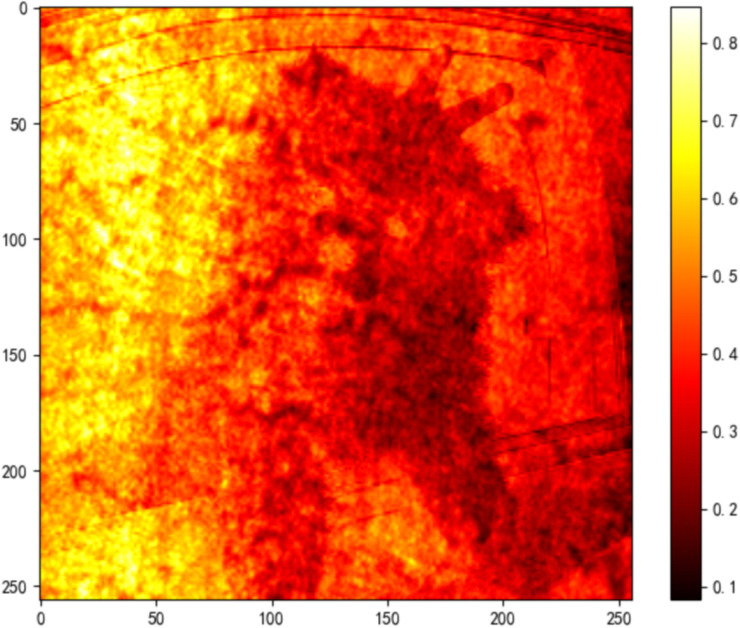
MSE heatmap of steel surface data under attack.

As shown in Figs 14–22, the experimental results reveal distinct patterns across defect categories. In weld defect detection, perturbations were concentrated mainly along weld boundaries, leading to blurred detection contours. The histogram overlap dropped to just 0.0724, with an average MSE of 0.0786, indicating a substantial reduction in localization accuracy. In the PCB defect scenario, despite the highly regular circuitry and clearly distinguishable features, Alpha perturbations still induced completely erroneous predictions. The histogram distributions showed nearly no overlap, and the mean MSE reached 0.0646, verifying the susceptibility of such highly structured data to transparency-channel attacks. The most extreme results occurred in metal surface crack detection, where perturbations not only drastically reduced detection accuracy but also triggered semantic-level recognition drift—that is, the model produced entirely unrelated class labels. In this case, the grayscale histogram distributions were fully separated (overlap ratio = 0.0000), and the mean MSE soared to 0.1990, far exceeding those observed in other scenarios, demonstrating the heightened sensitivity of complex textural defects to Alpha attacks.

Overall, the analysis shows that Alpha Channel Perturbations exhibit universal disruptive effects across different defect types and acquisition conditions. For structured geometries and fine-grained boundaries, they effectively degrade detection accuracy; for complex surface textures, they further induce semantic collapse, causing model outputs to deviate entirely from the ground truth. These findings indicate that Alpha attacks are not restricted to a specific dataset or acquisition setting but can systematically impair detection performance across defect categories, devices, and backgrounds. The combined use of grayscale histogram overlap and MSE provides intuitive quantitative characterization of this stealthy attack, reinforcing its practical relevance and universality in industrial environments. By conducting these extended experiments, we not only addressed concerns regarding dataset specificity but also established a more representative benchmark for subsequent research on robust defense strategies.

## 5. Conclusions

This study introduces a novel and stealthy adversarial paradigm—Alpha Channel Attack (ACA)—which leverages the transparency dimension of RGBA images to inject imperceptible perturbations. Unlike conventional RGB or metadata-based attacks, ACA exploits a long-overlooked blind spot in computer vision systems, fundamentally undermining visual perception integrity across object detection, generative modeling, and large-scale vision–language tasks. Extensive experiments confirm that ACA causes substantial degradation in mAP, FID, BLEU, METEOR, and CLIP Score, often exceeding the disruptive capacity of established attacks such as FGSM and Boundary Attack.

To counter this threat, we developed a lightweight detection framework based on histogram overlap and mean squared error (MSE) in the alpha domain. The proposed method achieves an exceptionally high AUC of 0.998, with only 3.2% false positives and a 4.6% attack success rate, demonstrating strong discriminative capability and practical deployability in real-world inspection pipelines.

Beyond demonstrating the vulnerability, this work also benchmarks ACA defenses against mainstream strategies. While adversarial training, denoising autoencoders, and diffusion-based defenses provide robust protection, they are computationally intensive and often lack scalability. In contrast, our Histogram + MSE detector offers a favorable trade-off between accuracy and efficiency, making it a promising choice for resource-constrained industrial environments.

These findings underscore three key contributions:

Scientific novelty: revealing alpha-channel perturbations as a new adversarial vector with cross-domain transferability.

1. Practical value: providing a cost-effective yet highly accurate detection mechanism suitable for deployment.
2. Strategic implications: highlighting the necessity of incorporating transparent-channel auditing into the broader paradigm of adversarial defense.

Future work will focus on three directions:

1. Robustness enhancement, integrating our detector with adversarial training or diffusion-based regularizers to withstand adaptive adversaries.
2. Cross-modal extension, evaluating ACA and its defenses in domains beyond vision-language, such as medical imaging and remote sensing.
3. Real-time deployment, optimizing the detection pipeline for embedded and mobile platforms, ensuring security without sacrificing latency.

Overall, this research advances both the theoretical and practical understanding of adversarial machine learning, laying a foundation for next-generation secure and resilient computer vision systems.

## Supporting information

S1 Fig Training process of the GAN under alpha channel perturbations. This figure displays the progressive evolution of generated samples during the training phase when alpha channel attacks are integrated.
(PDF)

S2 Fig Sample images of external threads with alpha channel attacks. A comprehensive set of thread images containing invisible adversarial perturbations in the transparency layer, used to test the system’s vulnerability.
(PDF)

S3 Fig Comparison of GAN training dynamics and convergence. This figure illustrates the generative process under alternative training configurations (e.g., baseline comparison or different noise levels) to demonstrate the consistency of the GAN’s performance.
(PDF)

S4 File Contains the adversarial attack images (S1–S3 Figs.) used in this study, which are essential for reproducing the experimental results and verifying the proposed visual security defense method.
(ZIP)
