# Supplementary figures and images for: Visual security defense for industrial inspection based on computer vision (part 1 of 2)
Source: PLoS One. 2026 Feb 4;21(2):e0338835. doi: 10.1371/journal.pone.0338835 (PMC12872028; doi:10.1371/journal.pone.0338835)

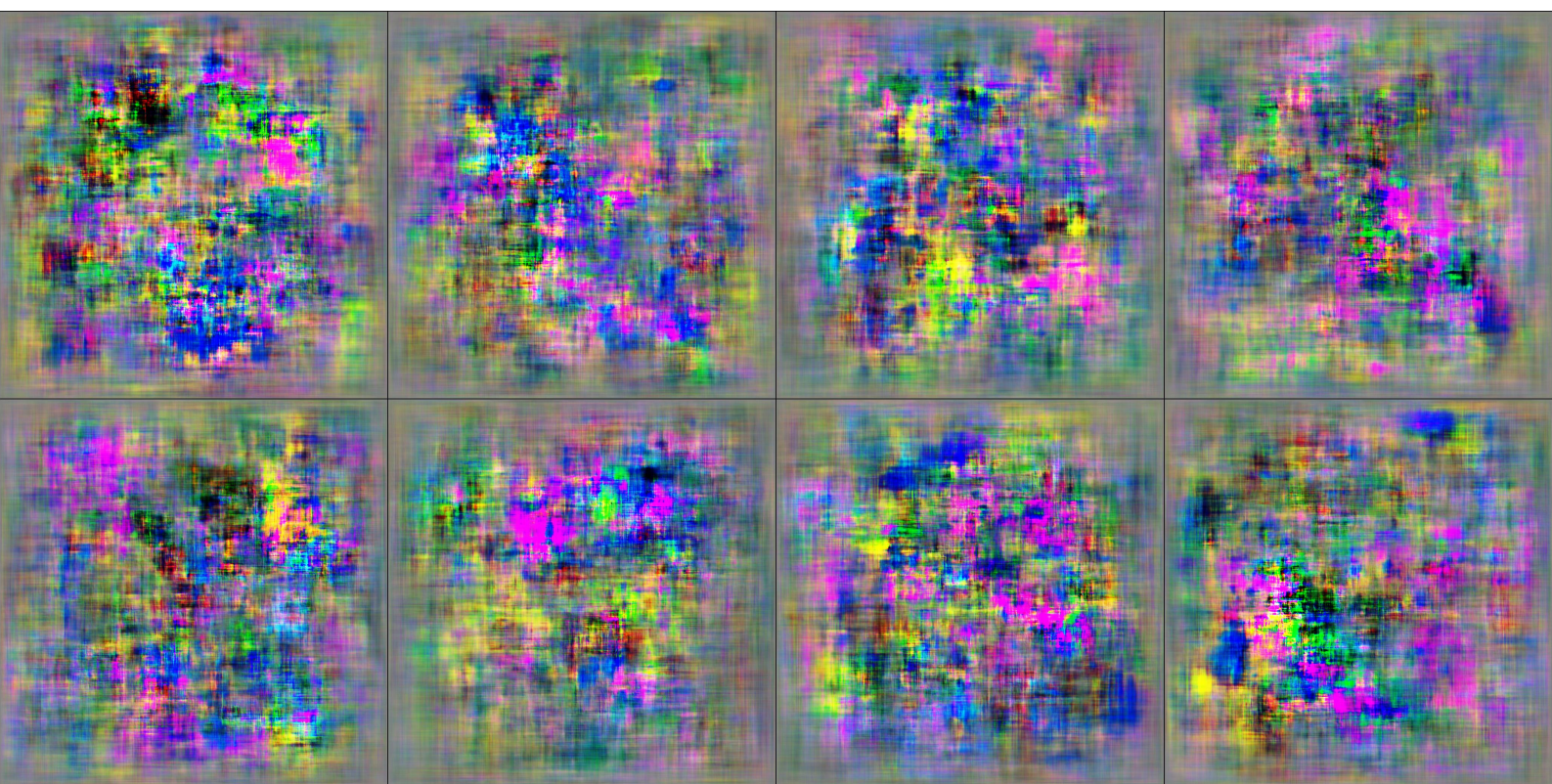

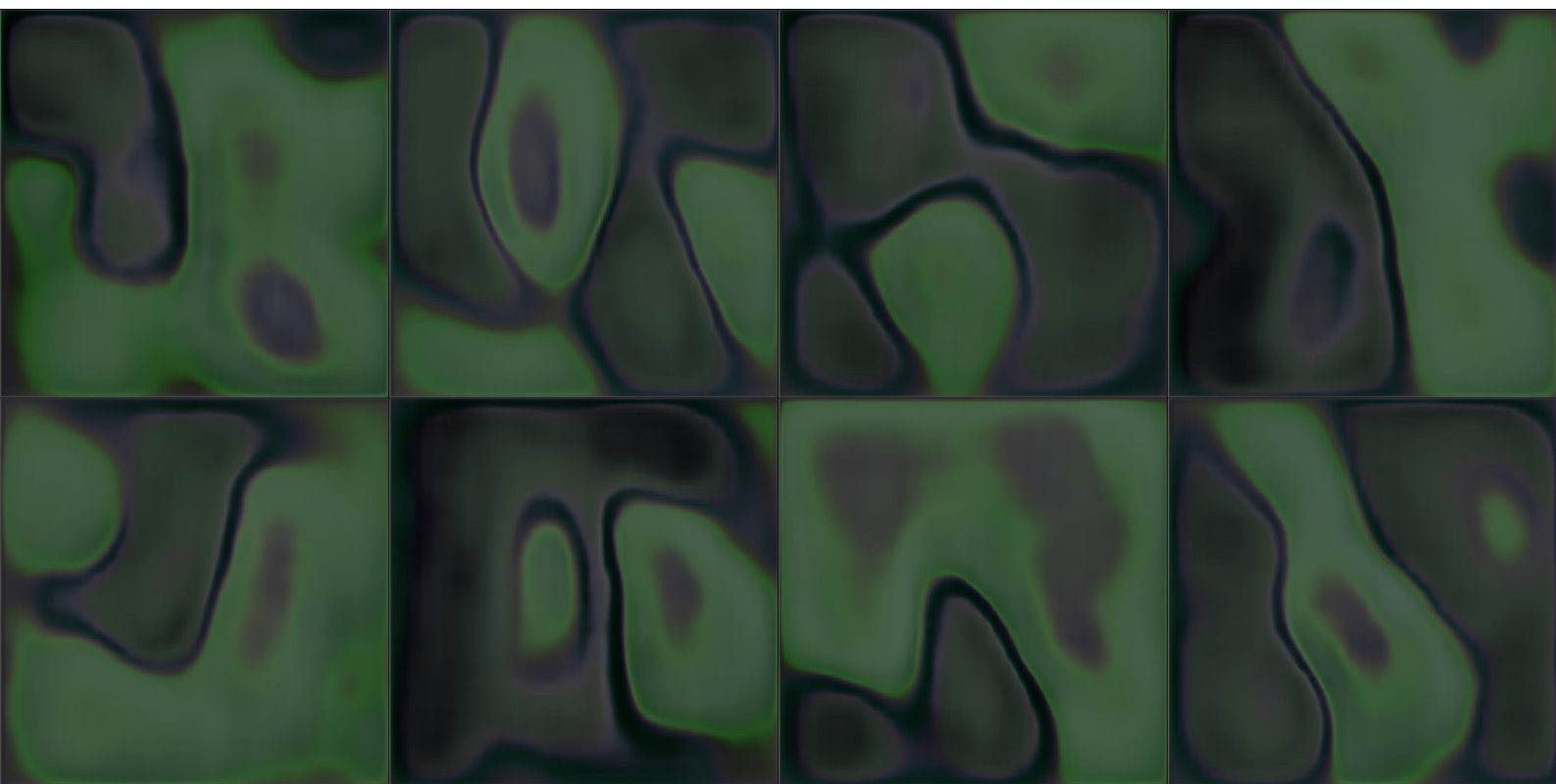

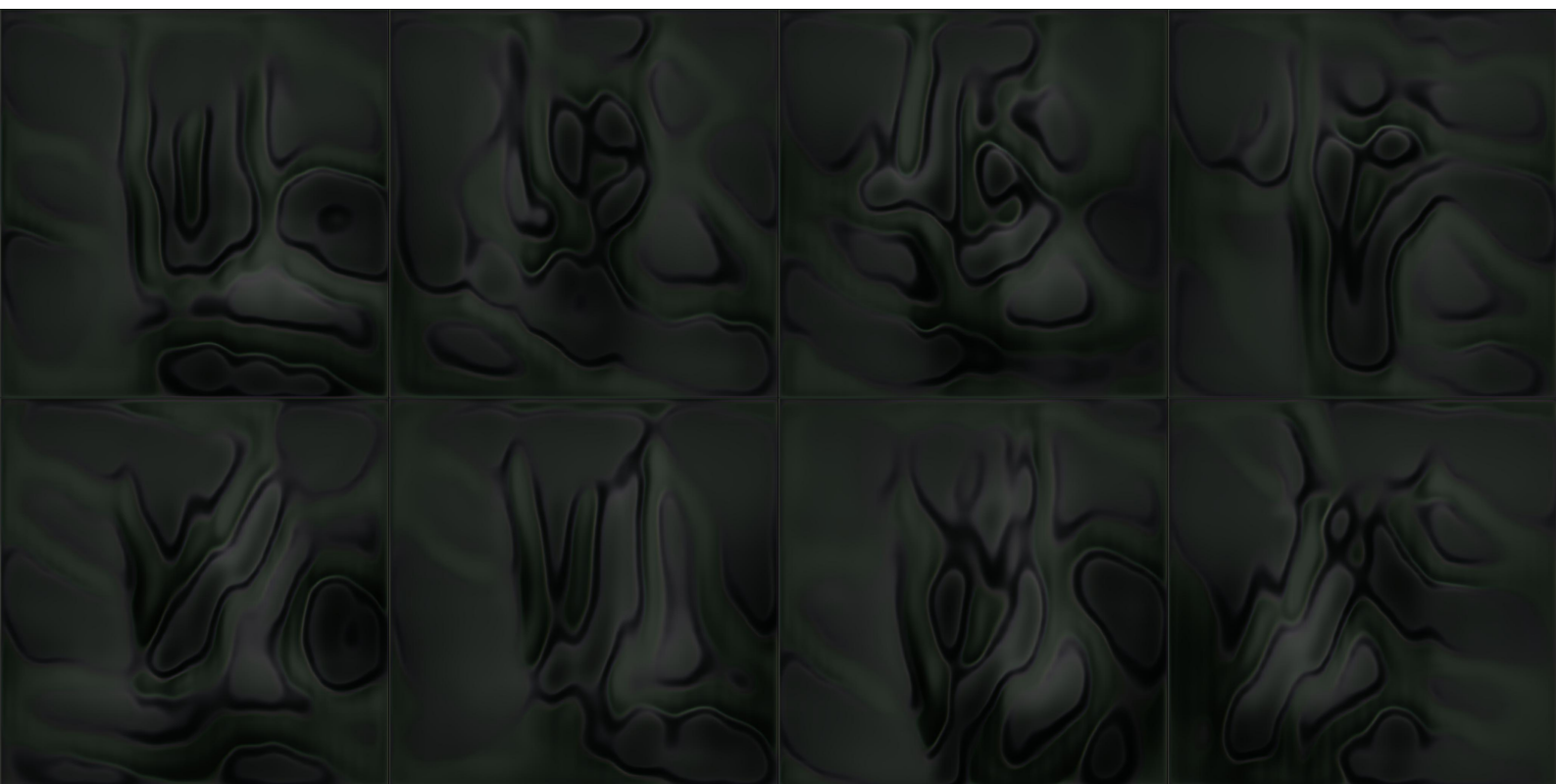

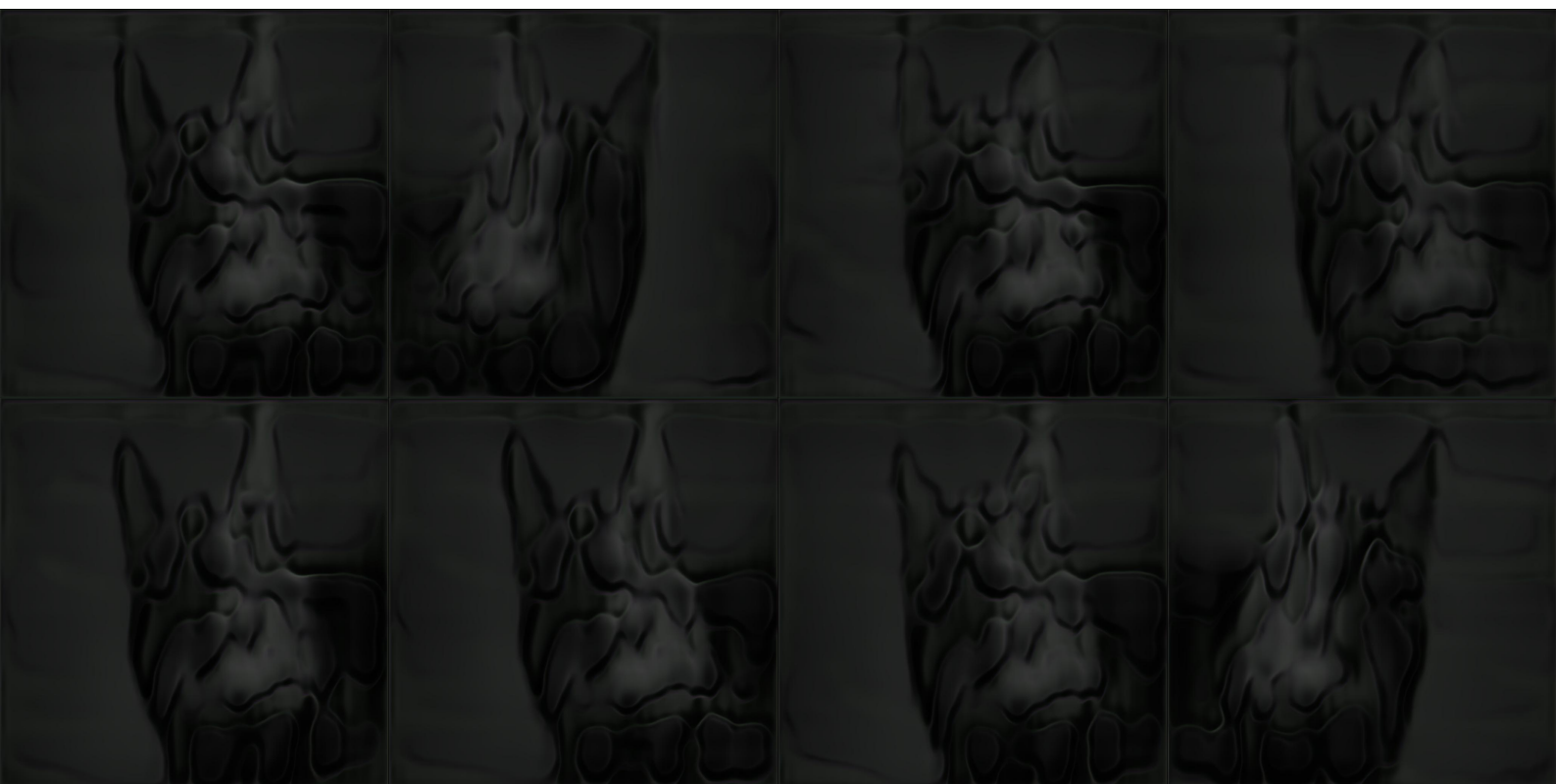

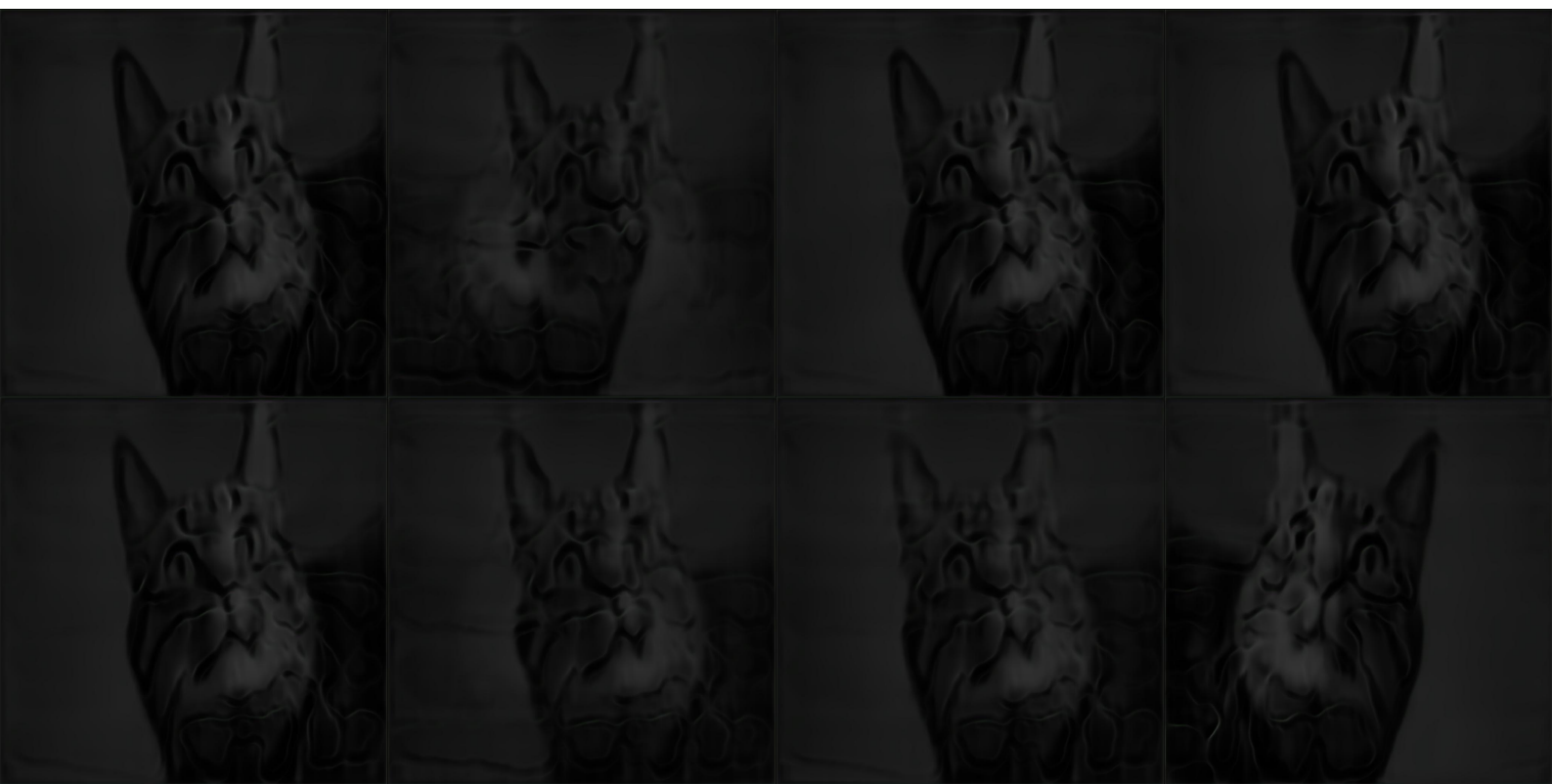

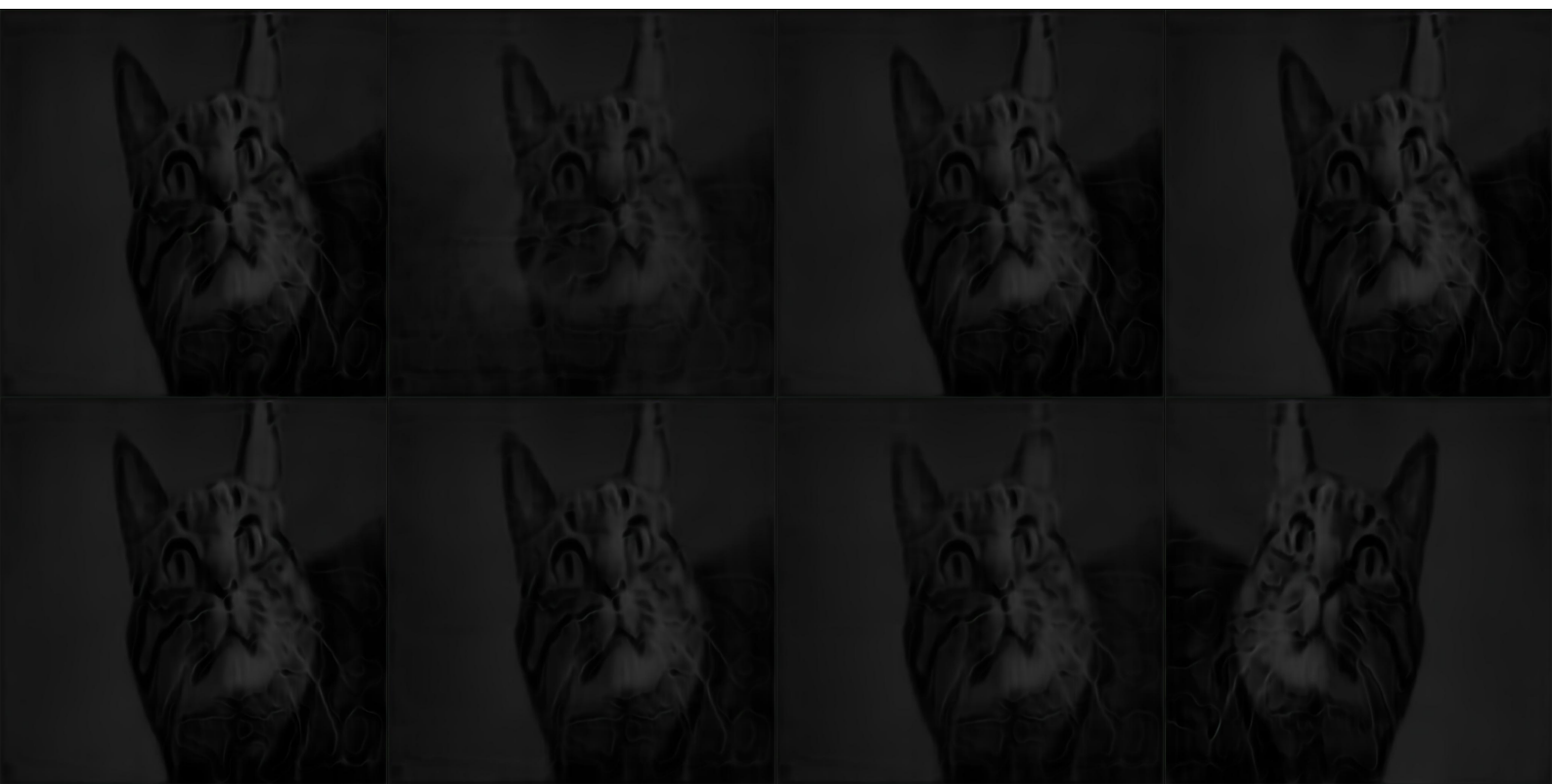

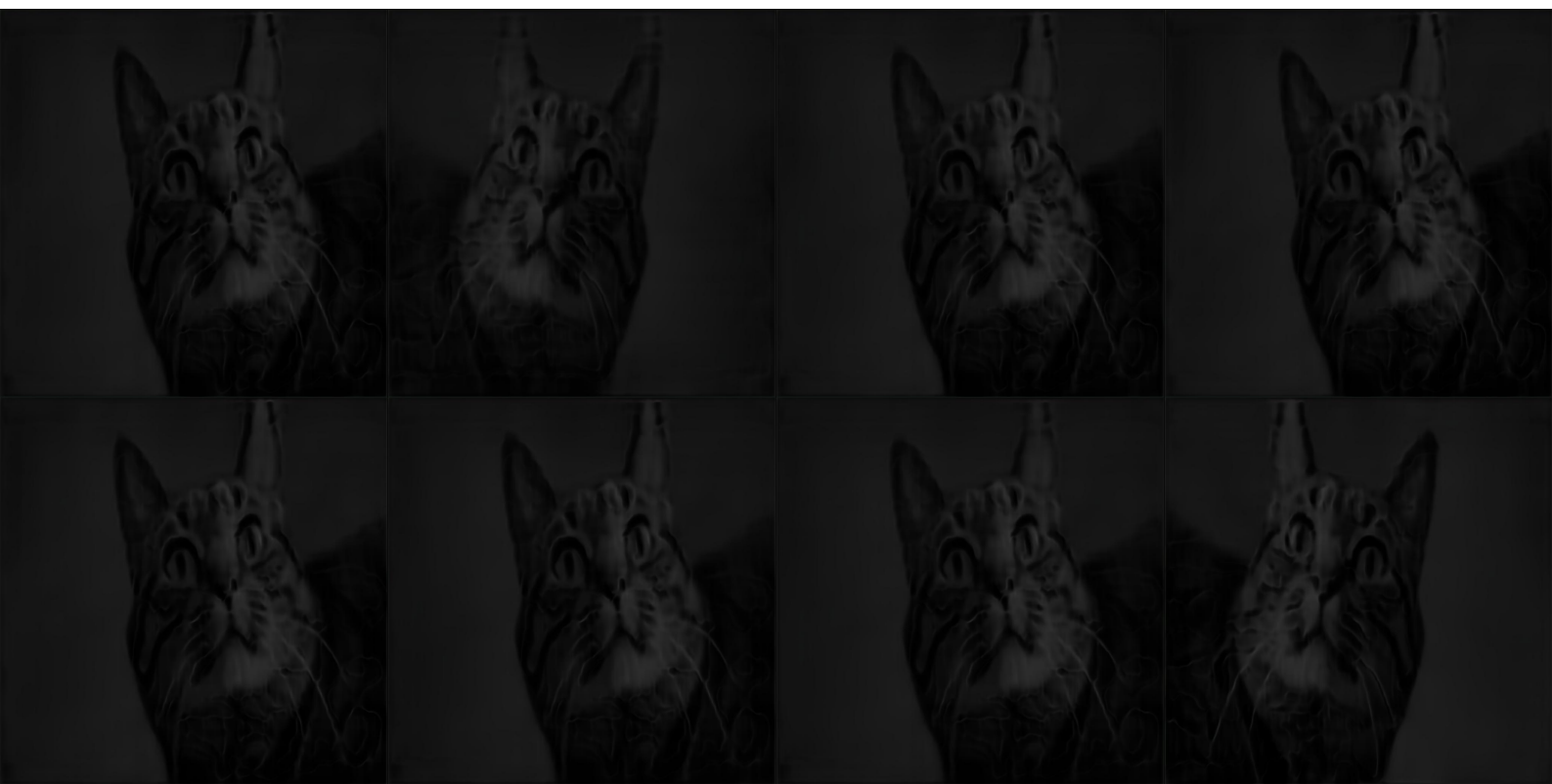

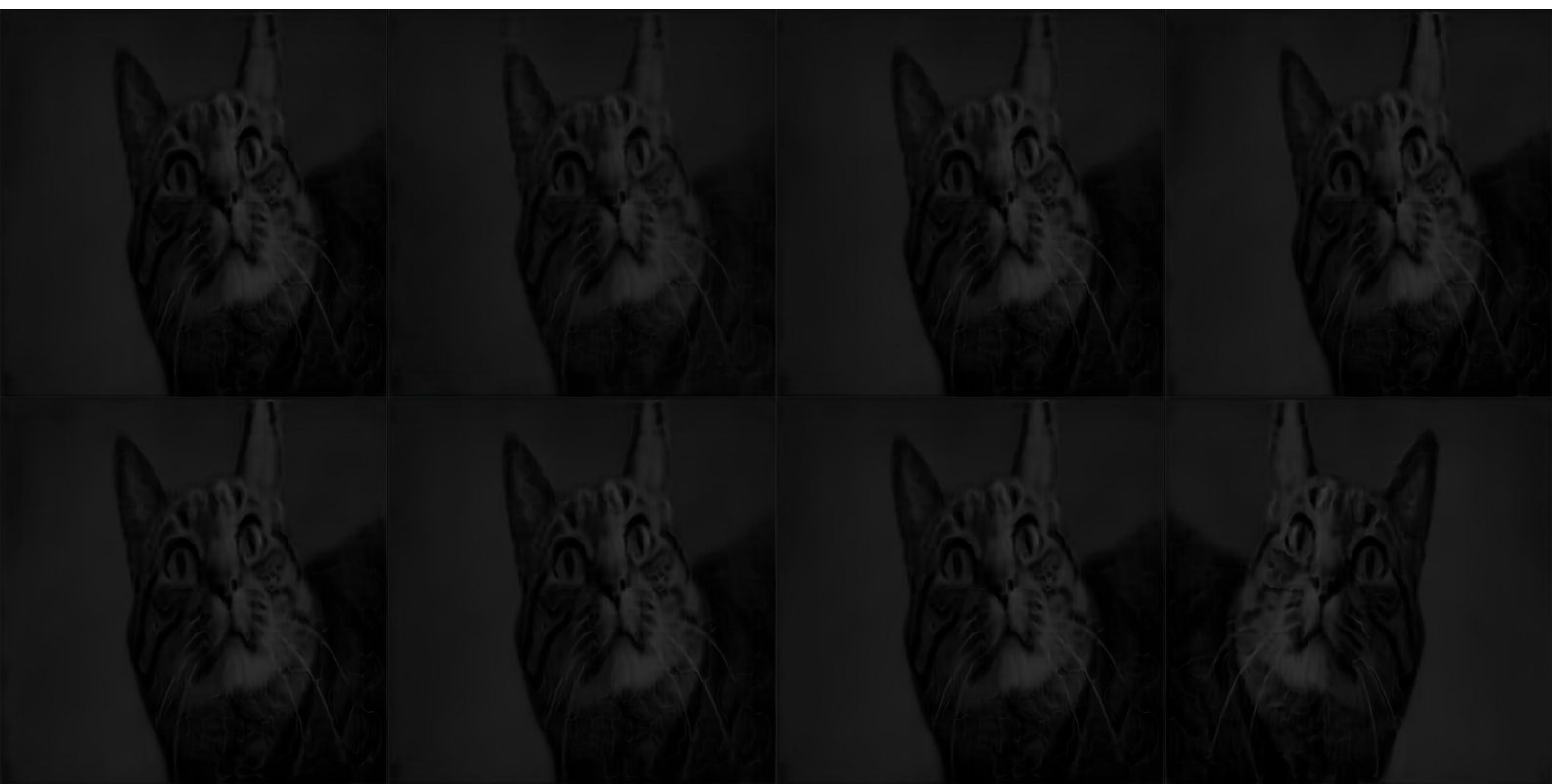

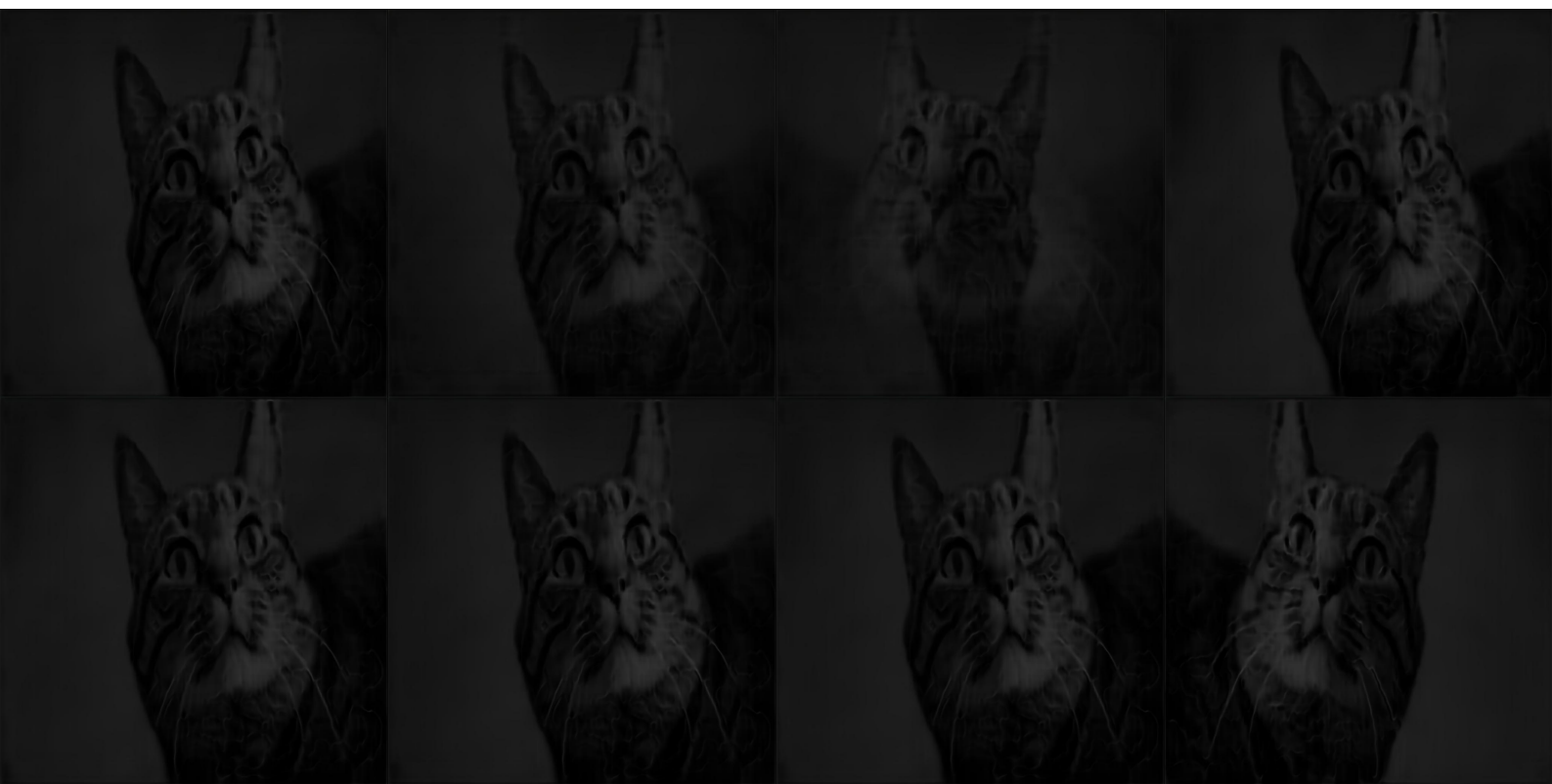

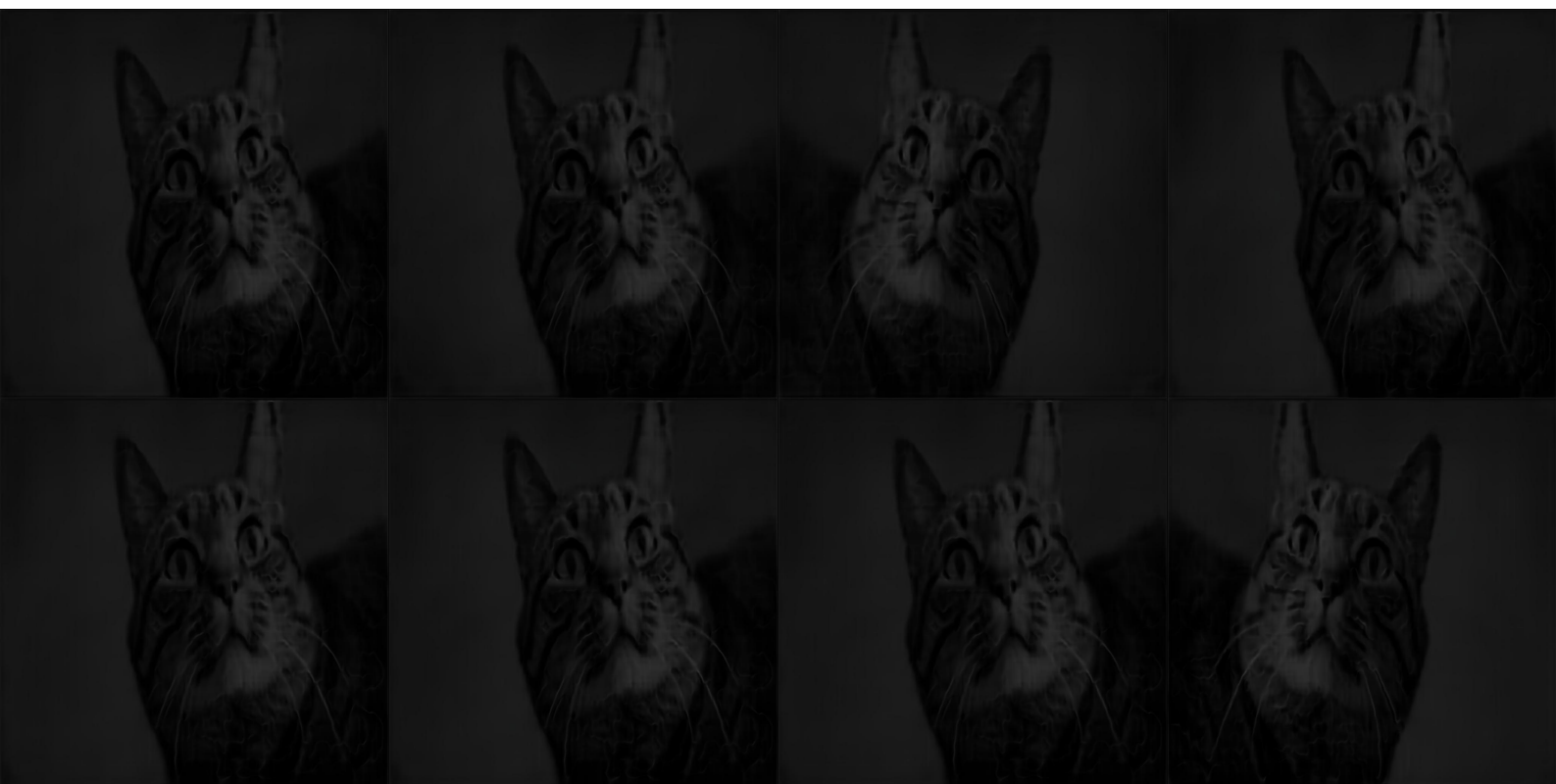

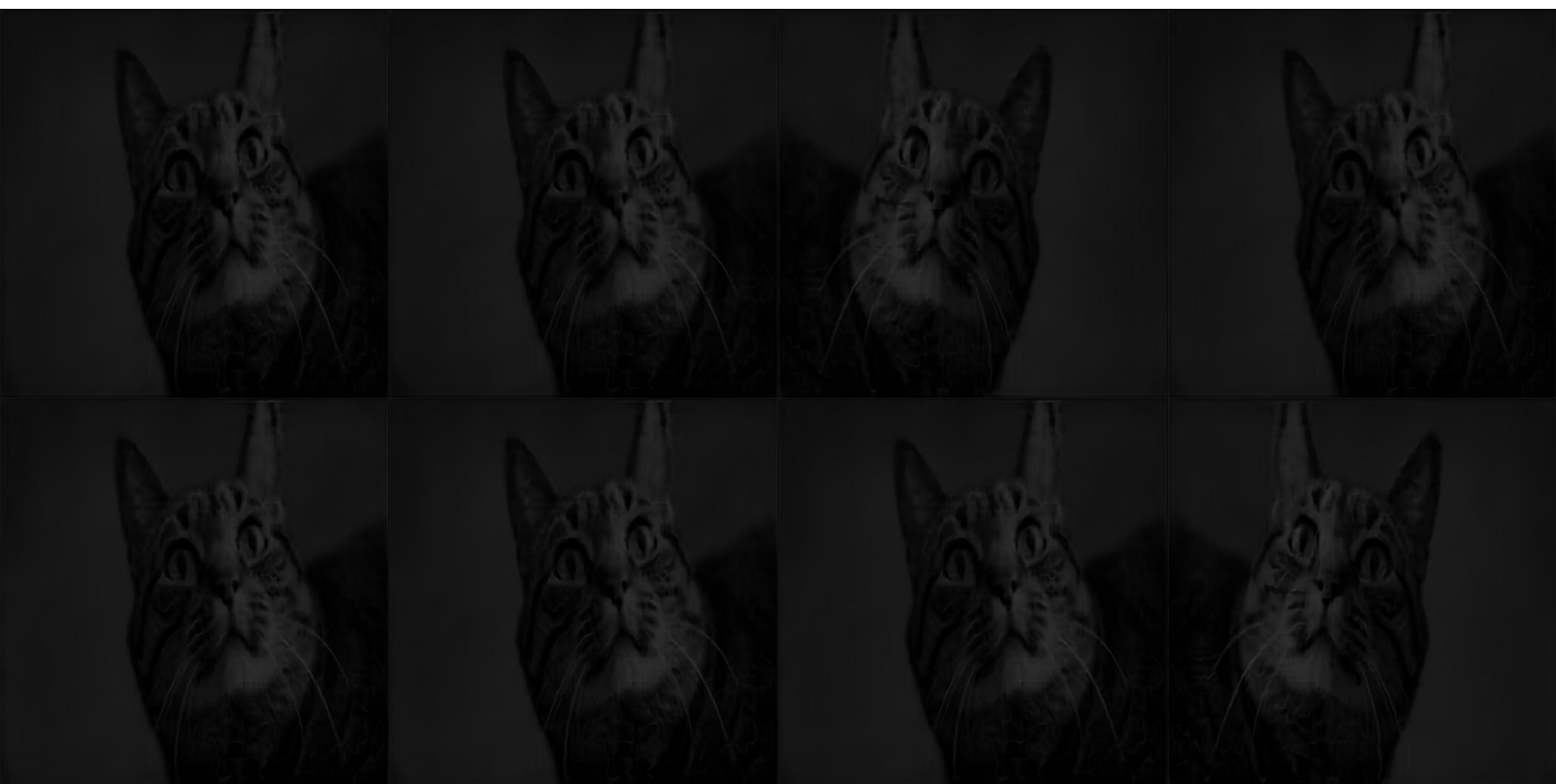

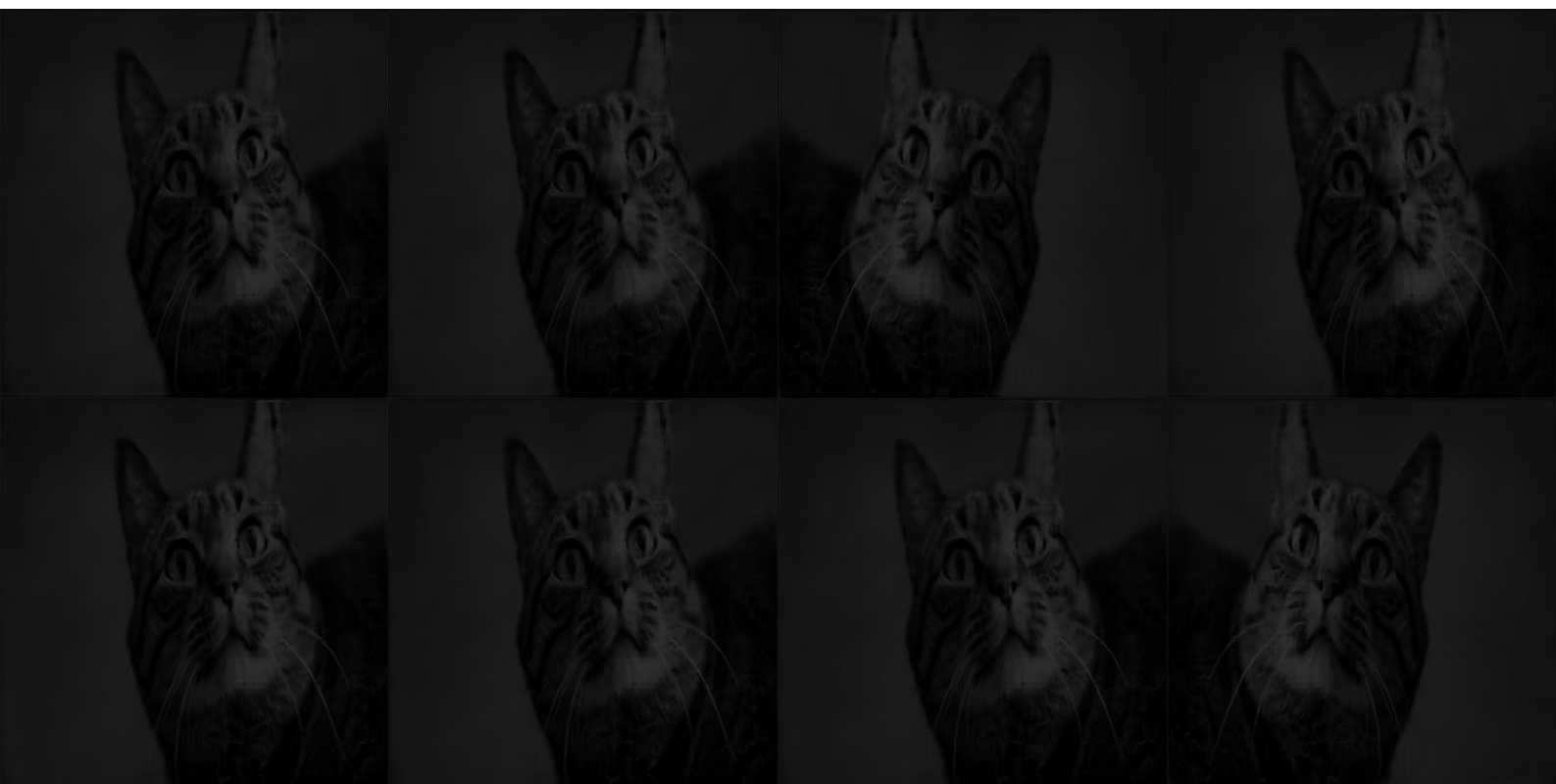

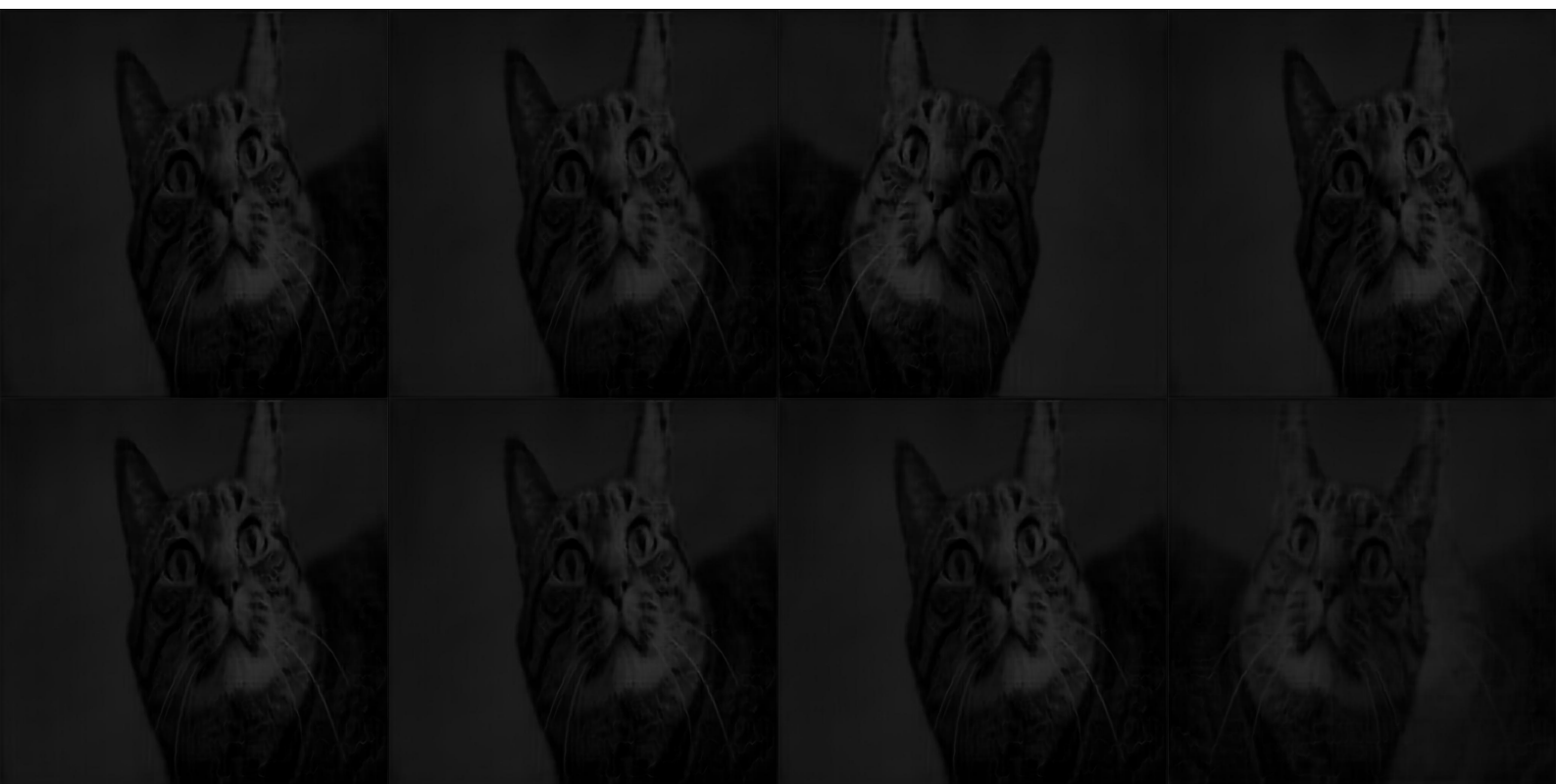

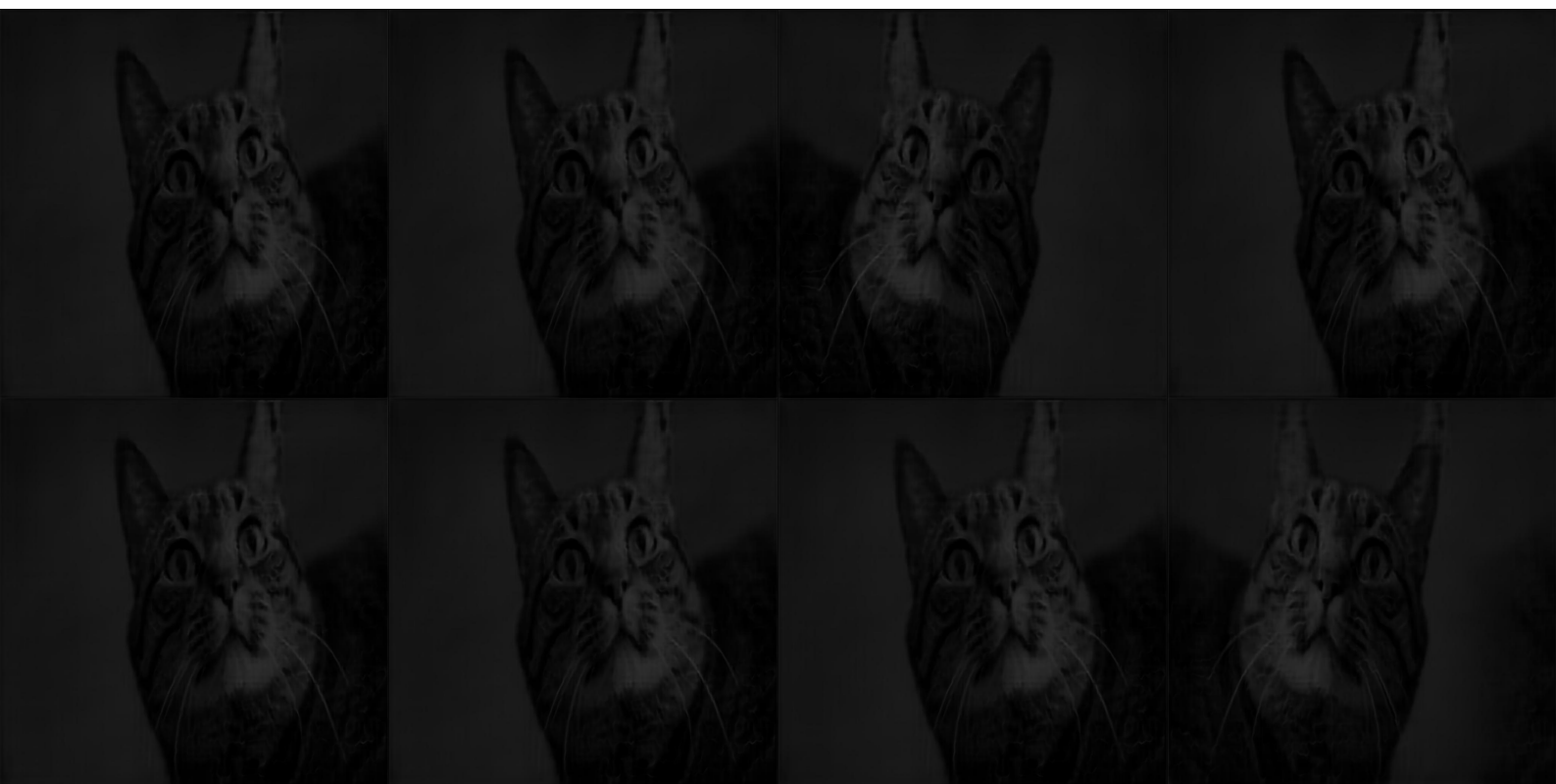

Supplement: S1 Fig — This figure displays the progressive evolution of generated samples during the training phase when alpha channel attacks are integrated. (PDF) [file pone.0338835.s001.pdf]

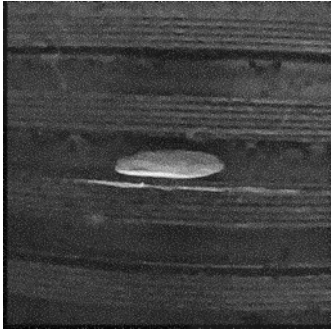

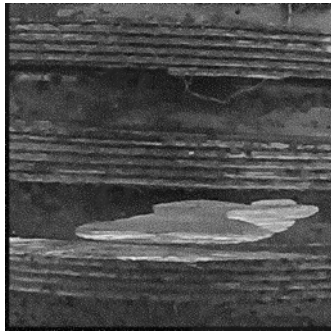

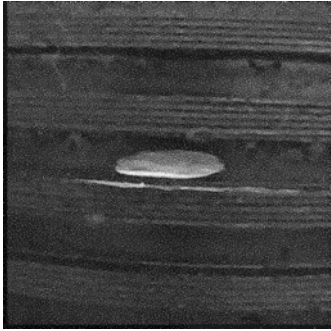

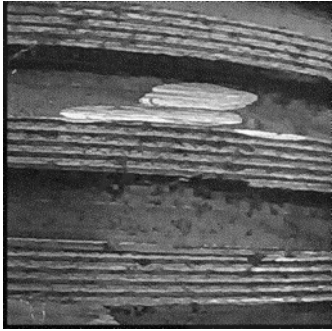

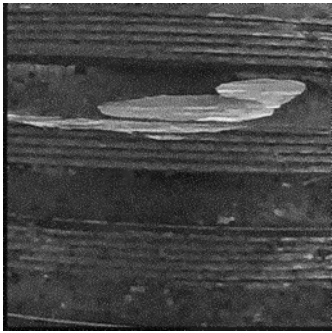

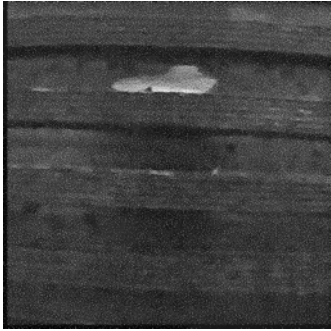

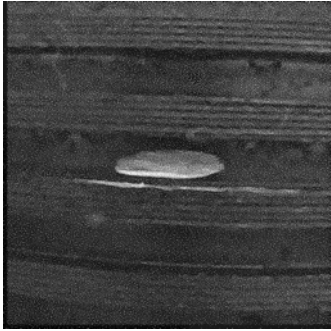

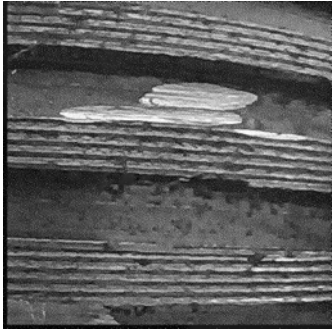

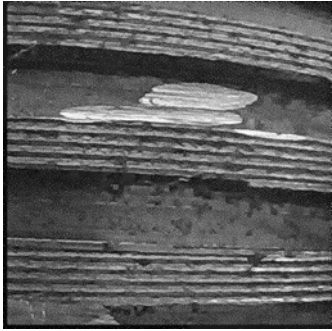

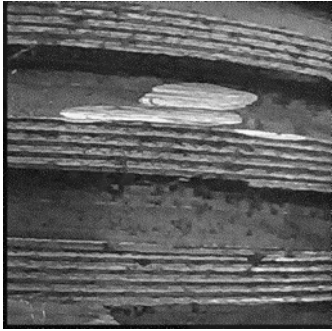

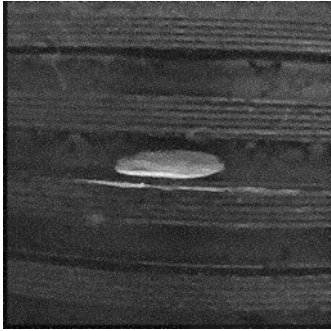

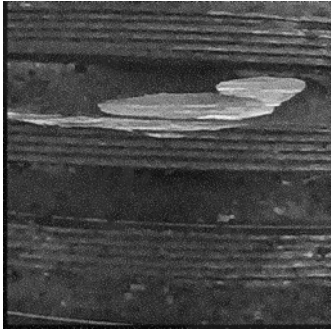

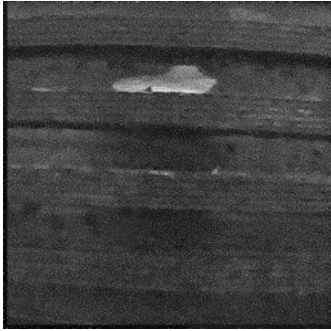

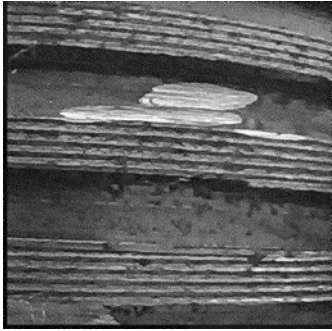

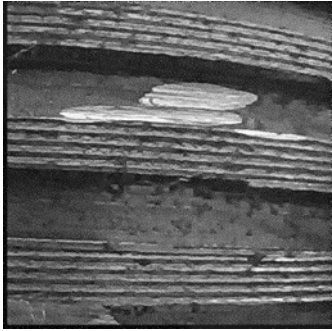

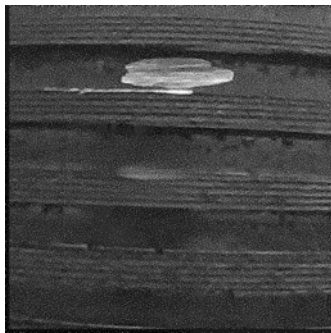

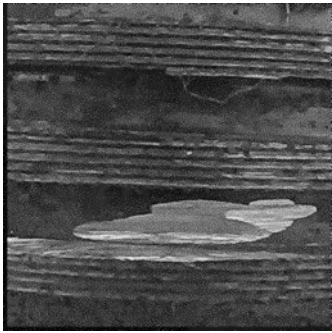

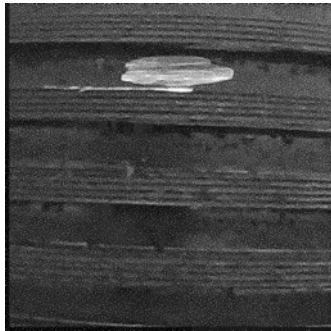

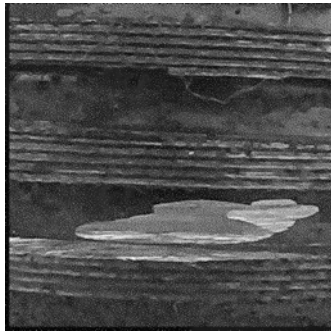

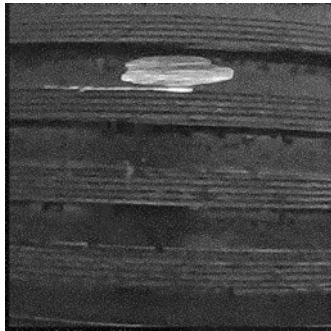

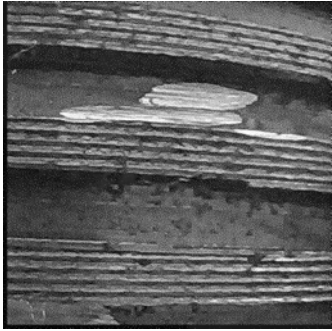

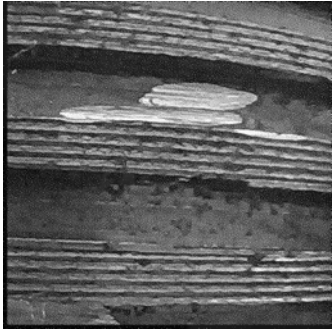

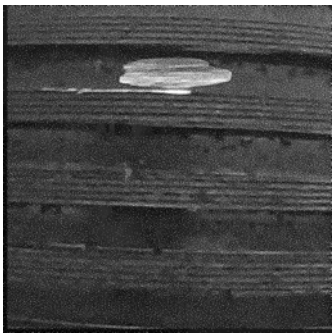

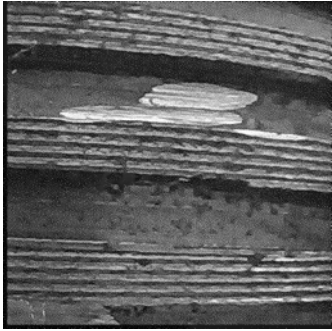

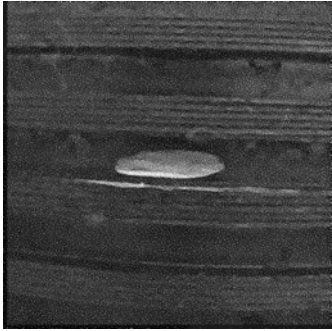

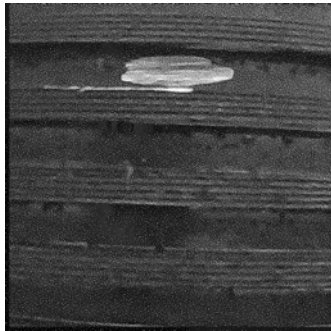

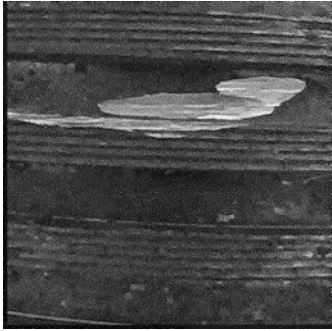

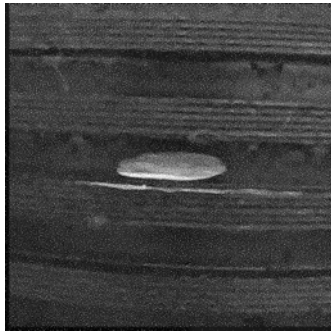

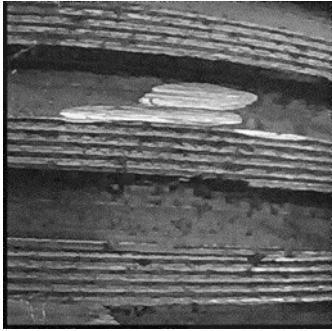

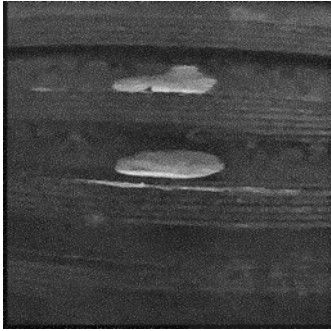

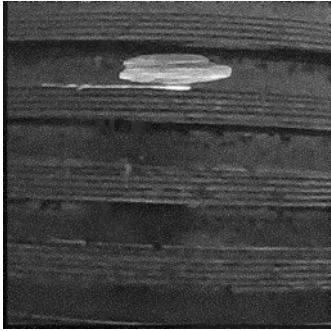

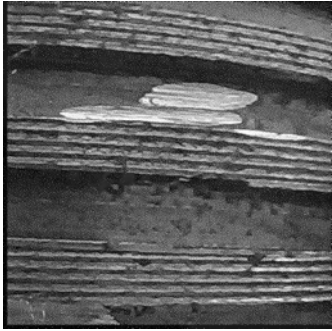

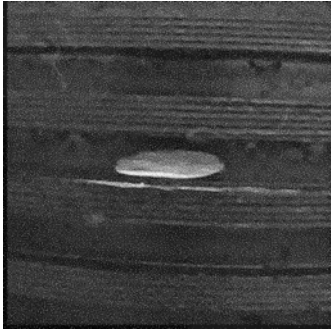

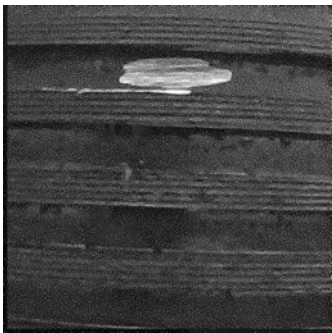

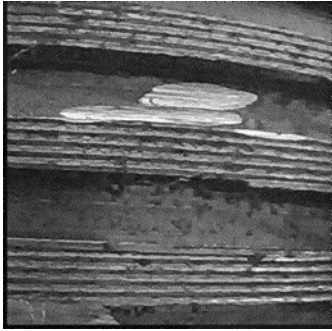

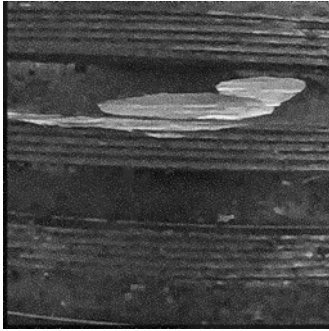

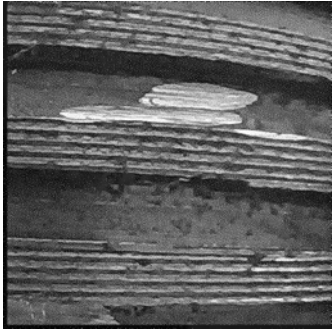

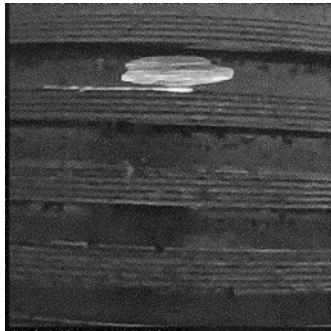

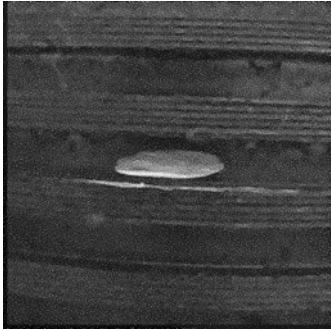

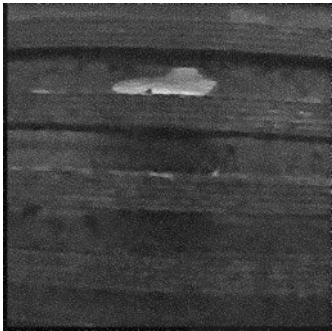

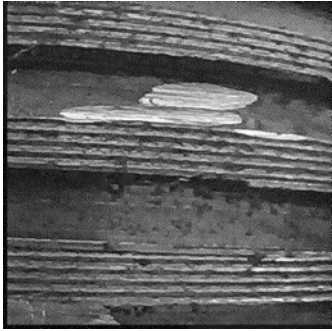

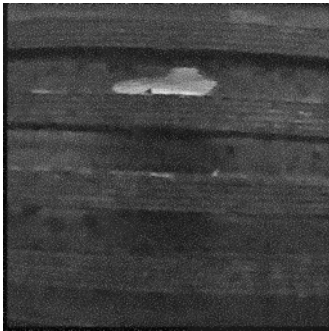

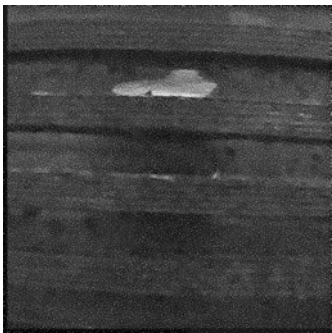

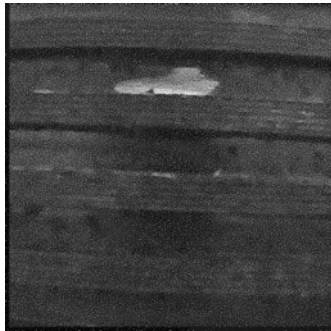

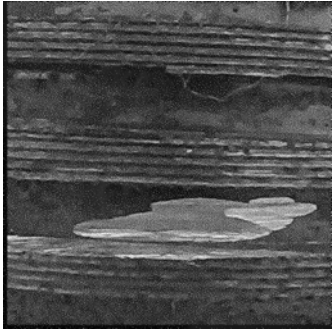

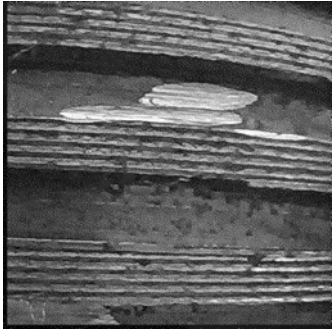

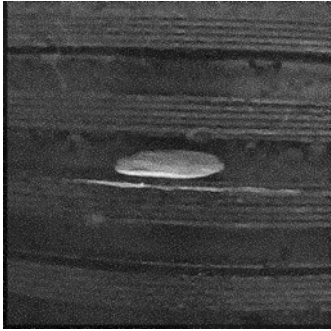

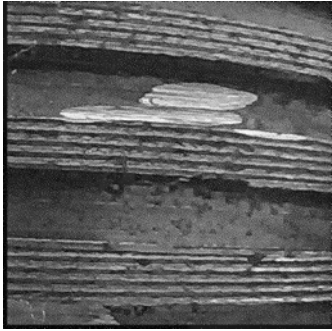

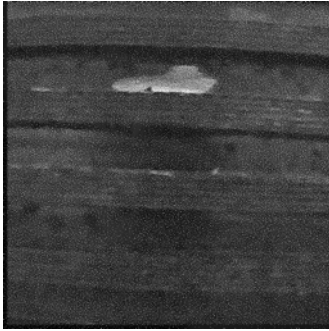

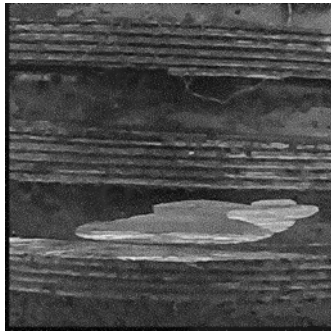

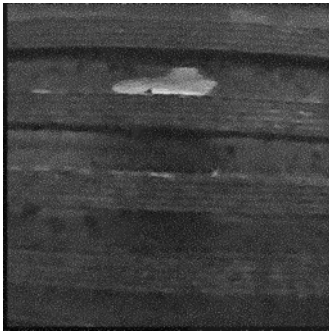

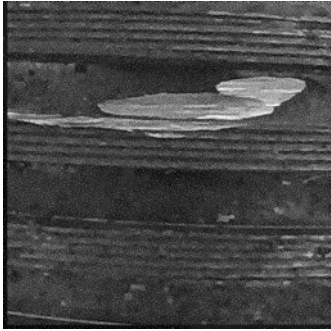

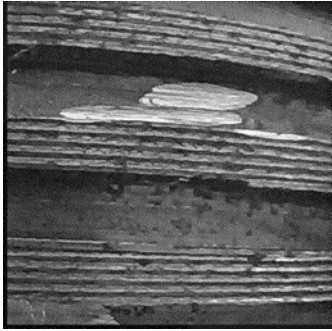

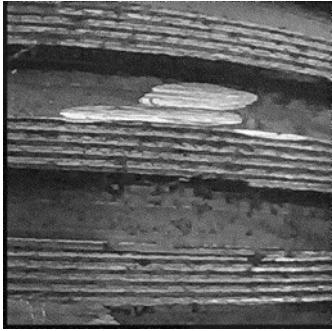

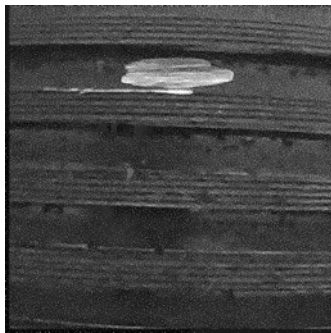

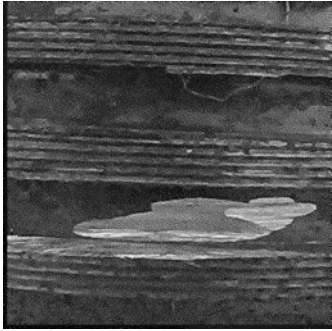

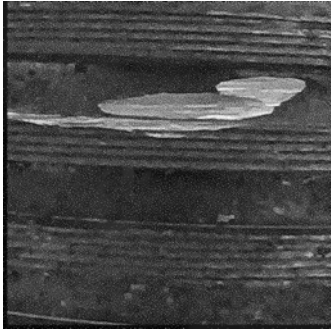

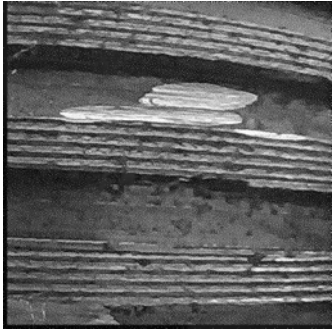

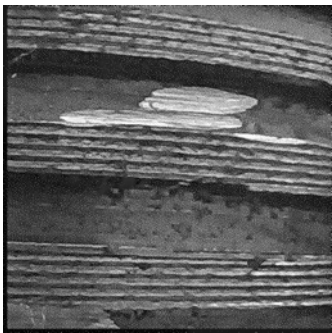

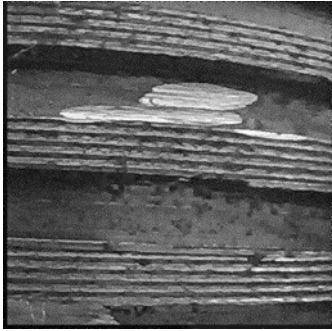

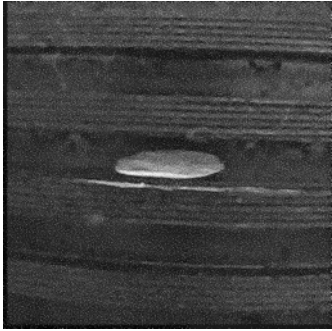

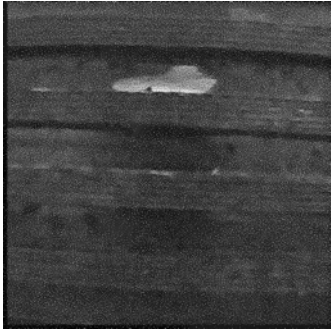

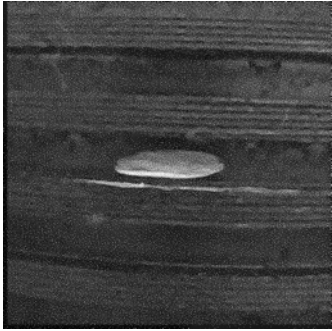

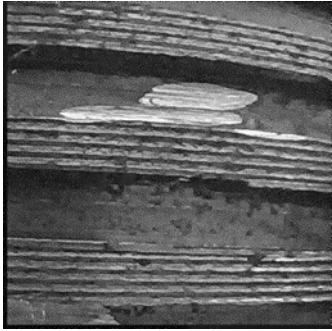

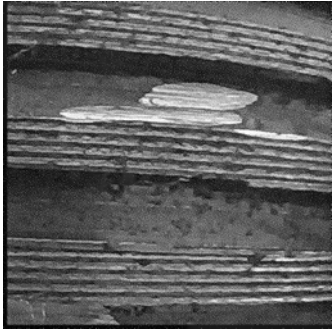

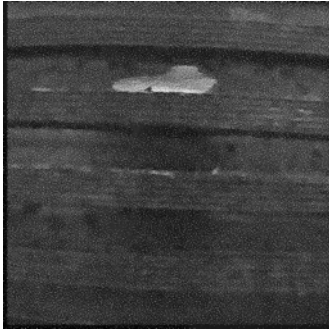

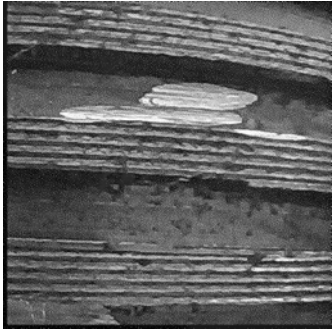

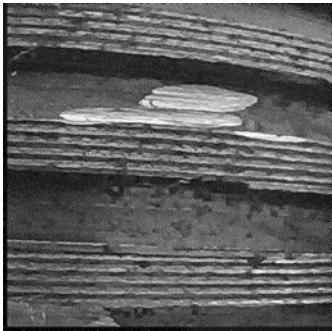

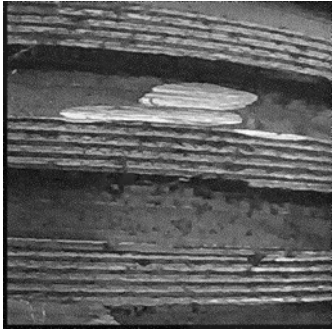

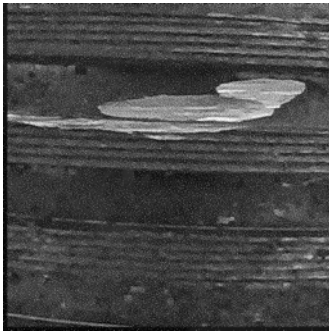

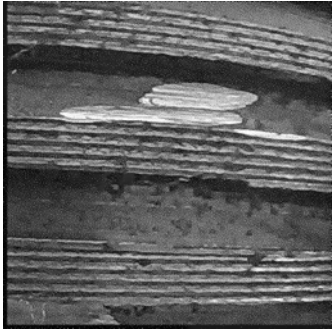

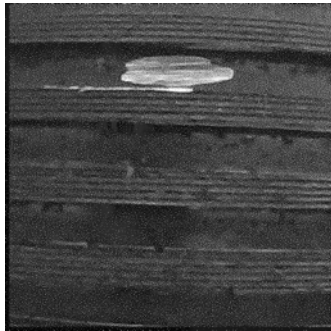

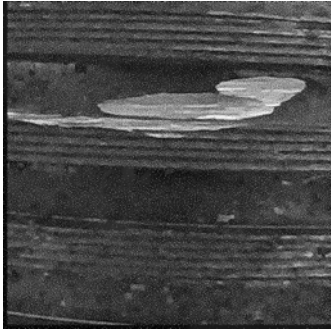

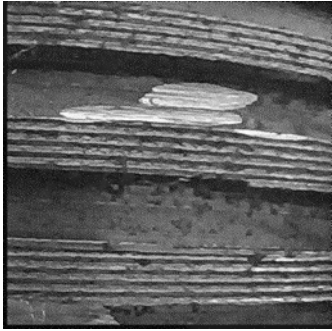

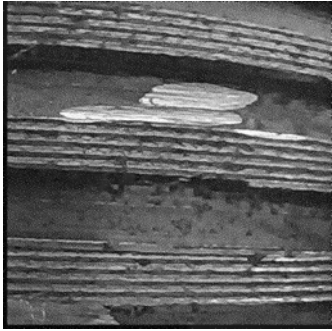

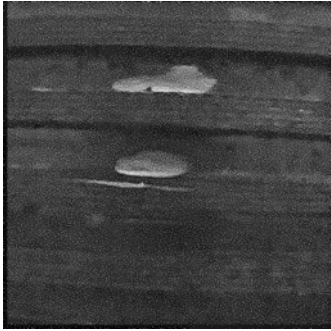

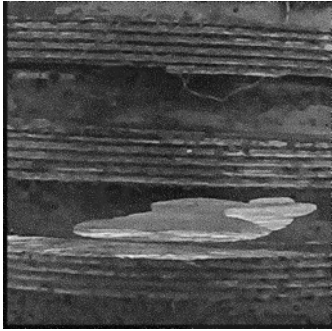

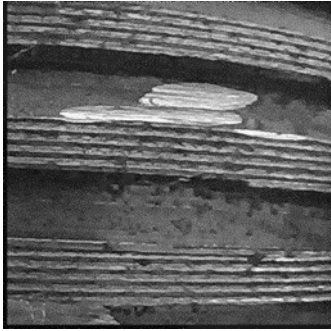

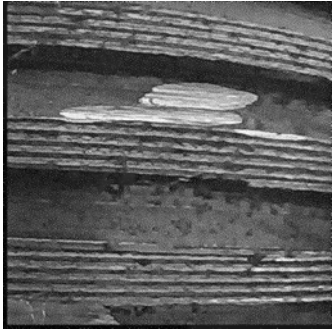

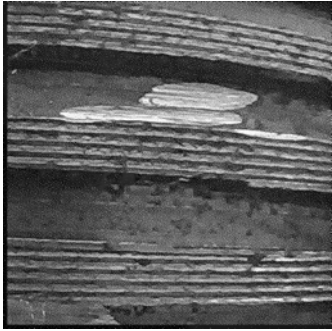

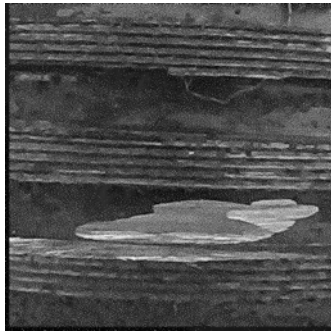

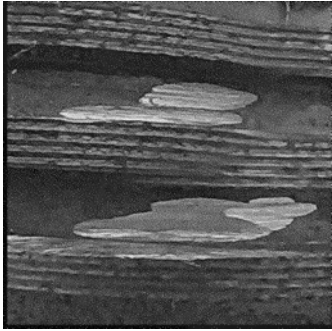

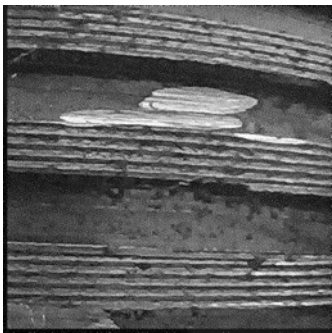

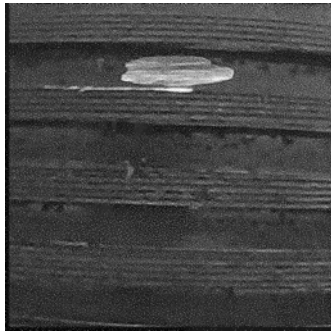

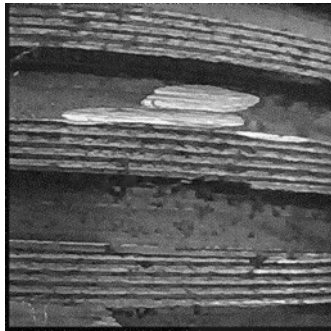

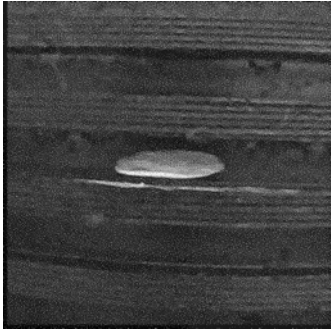

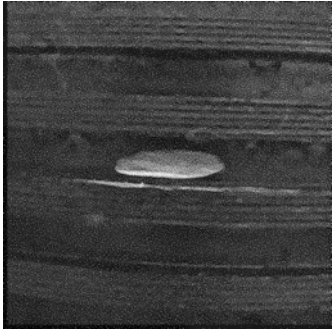

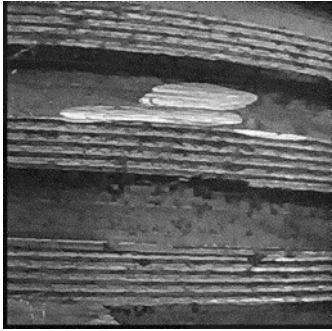

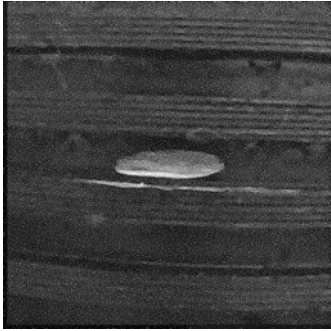

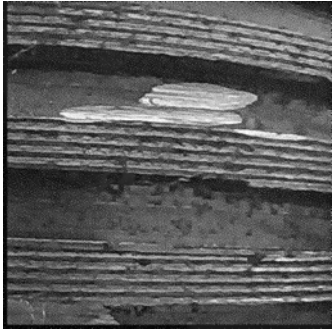

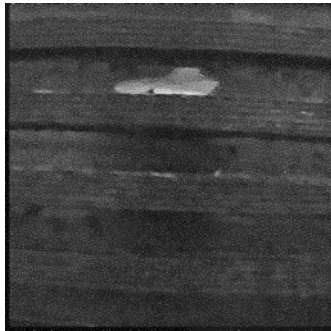

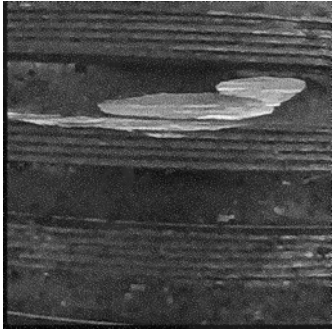

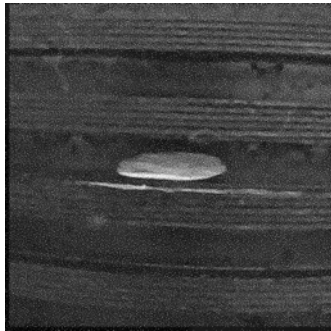

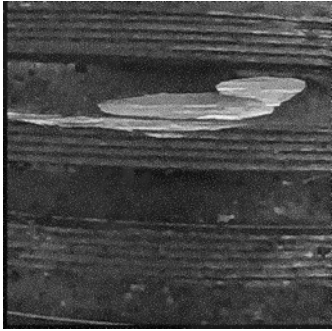

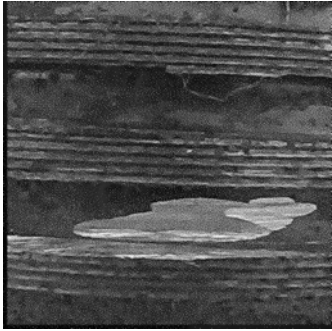

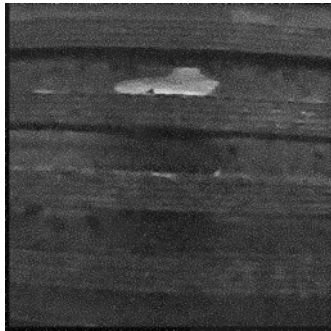

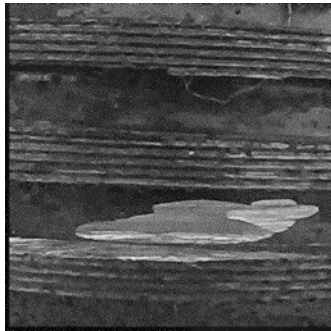

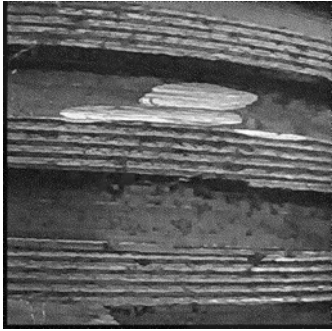

Supplement: S2 Fig — A comprehensive set of thread images containing invisible adversarial perturbations in the transparency layer, used to test the system’s vulnerability. (PDF) [file pone.0338835.s002.pdf]

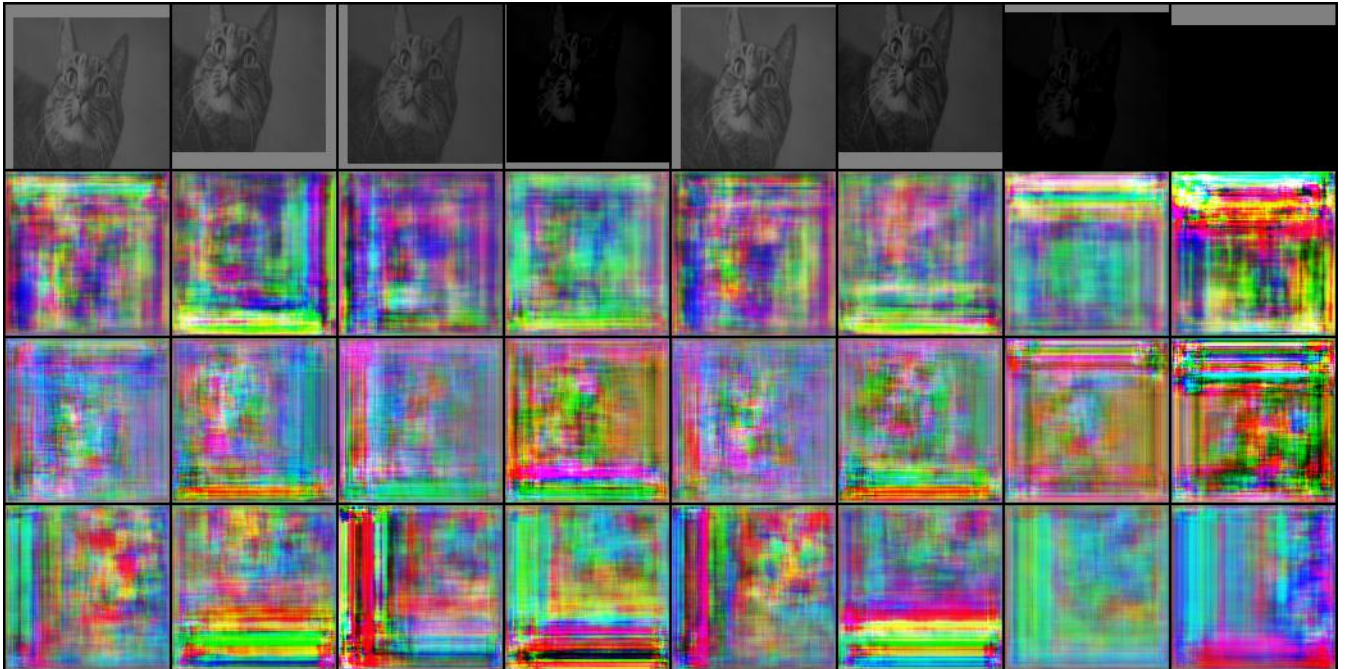

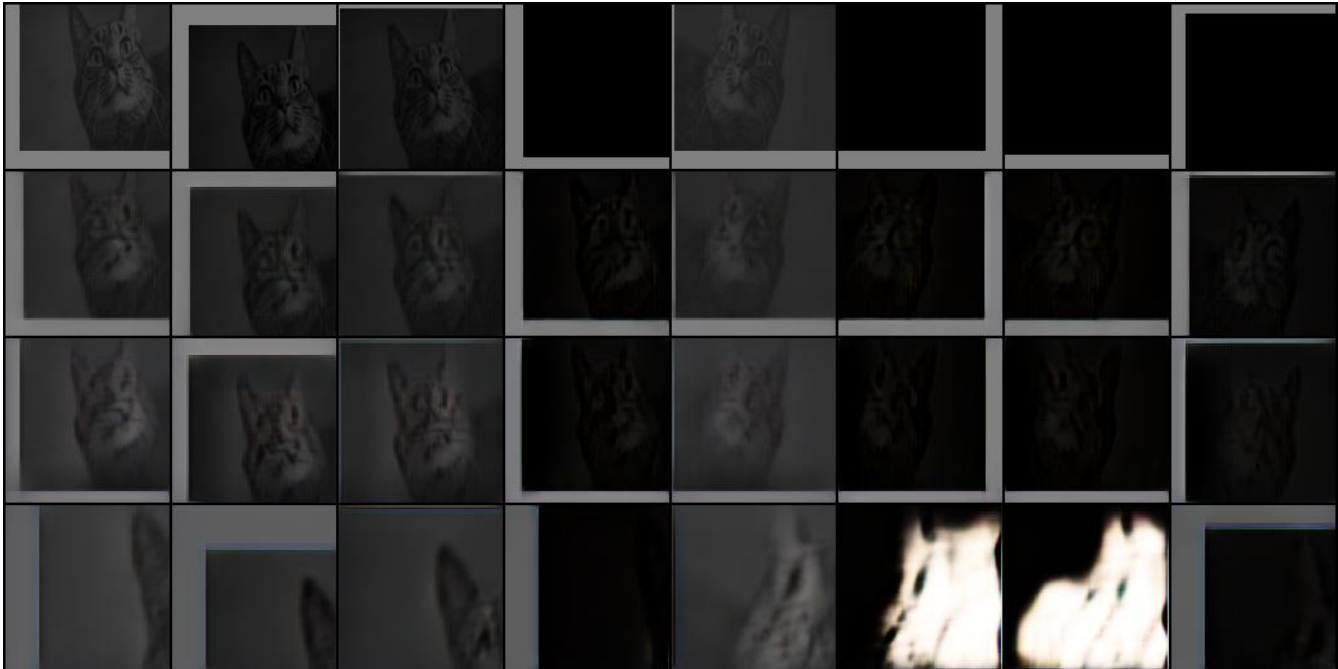

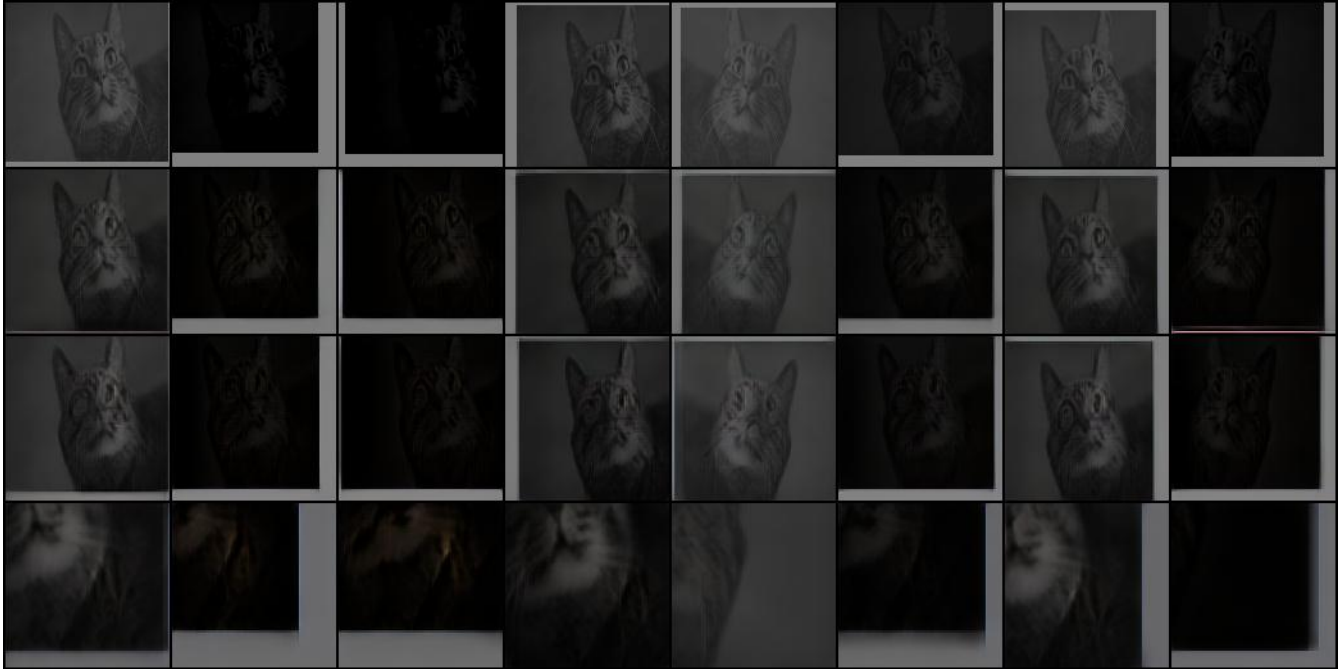

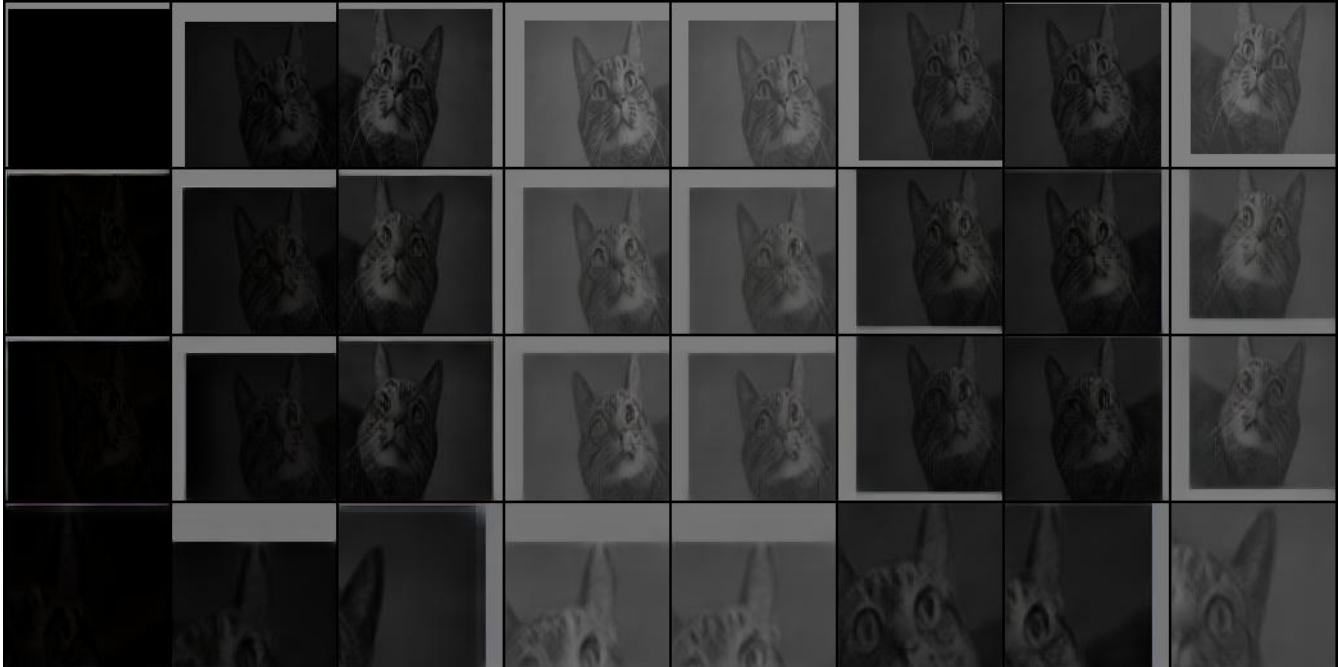

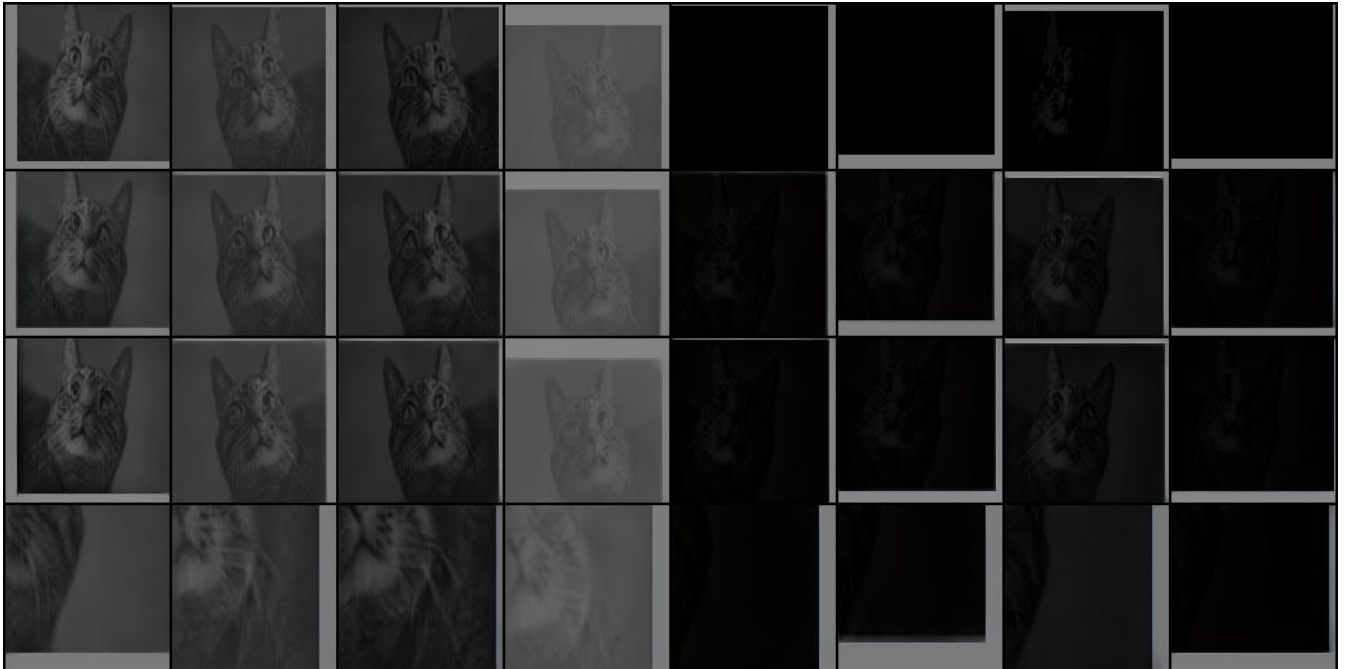

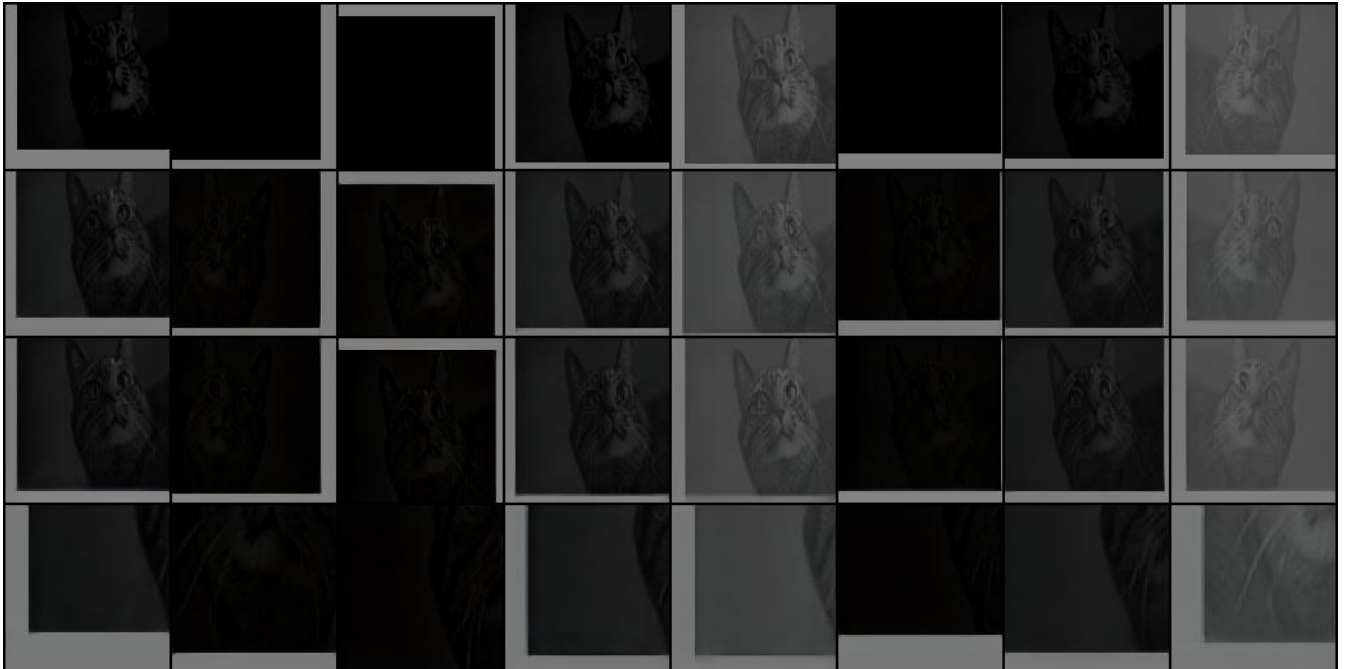

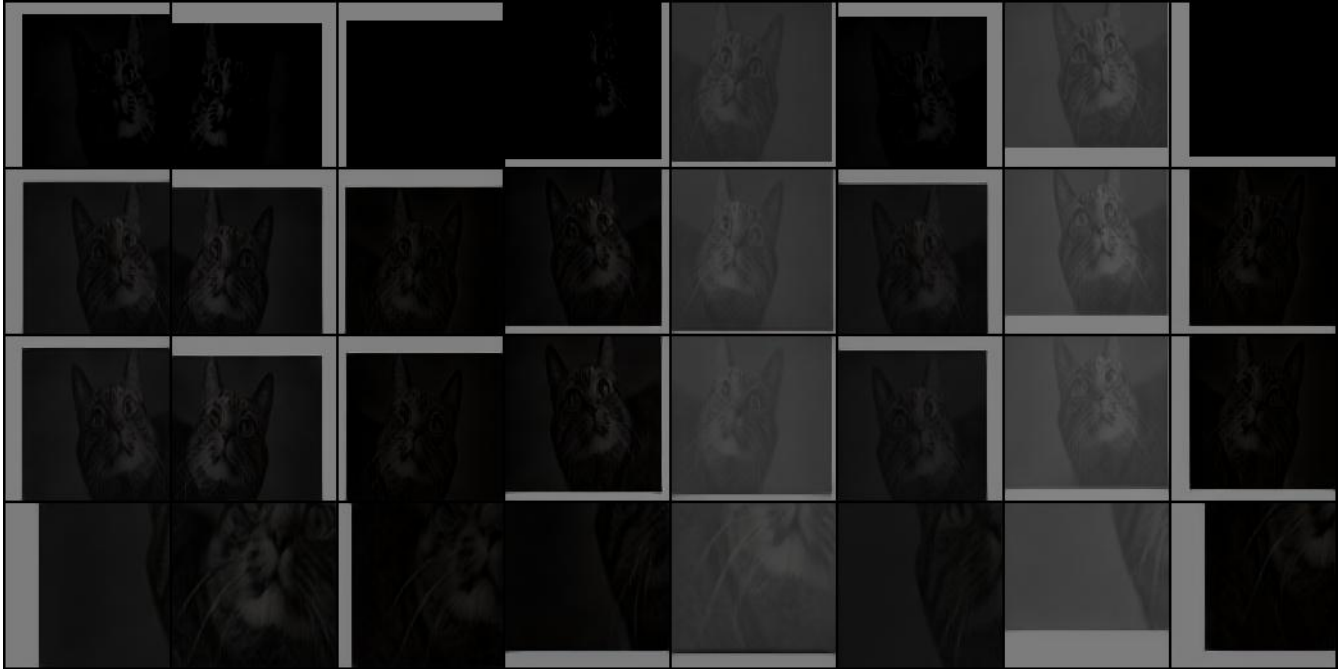

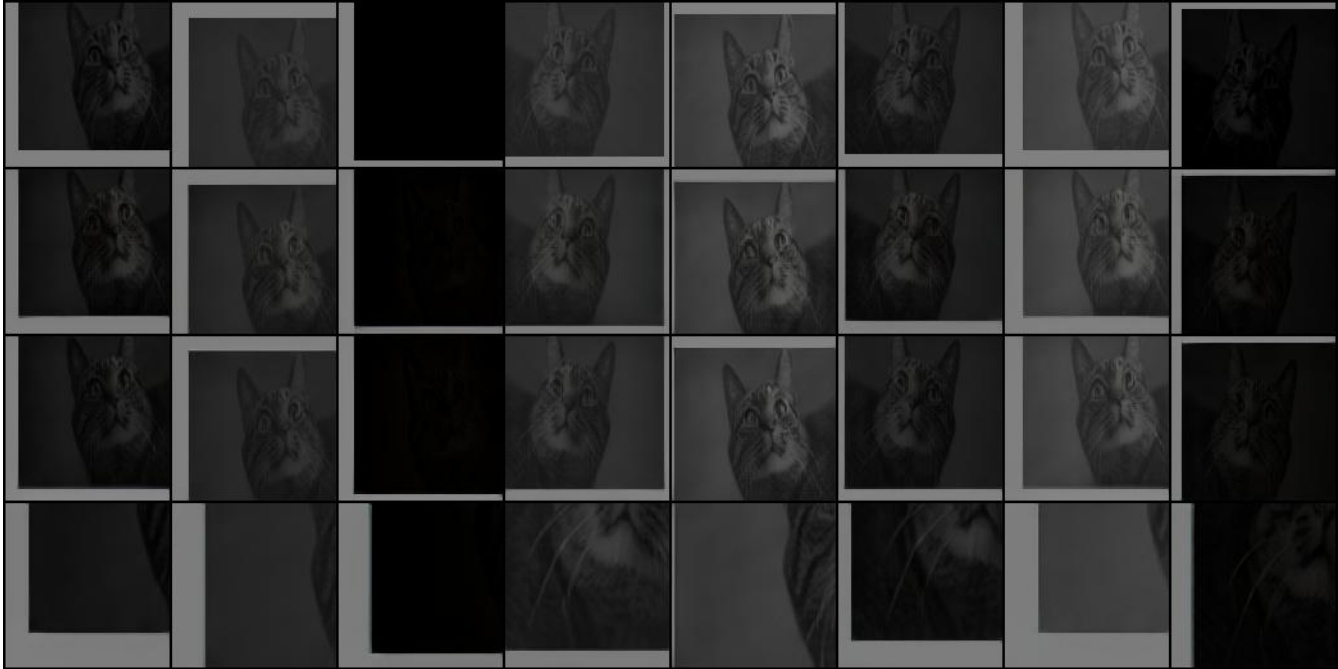

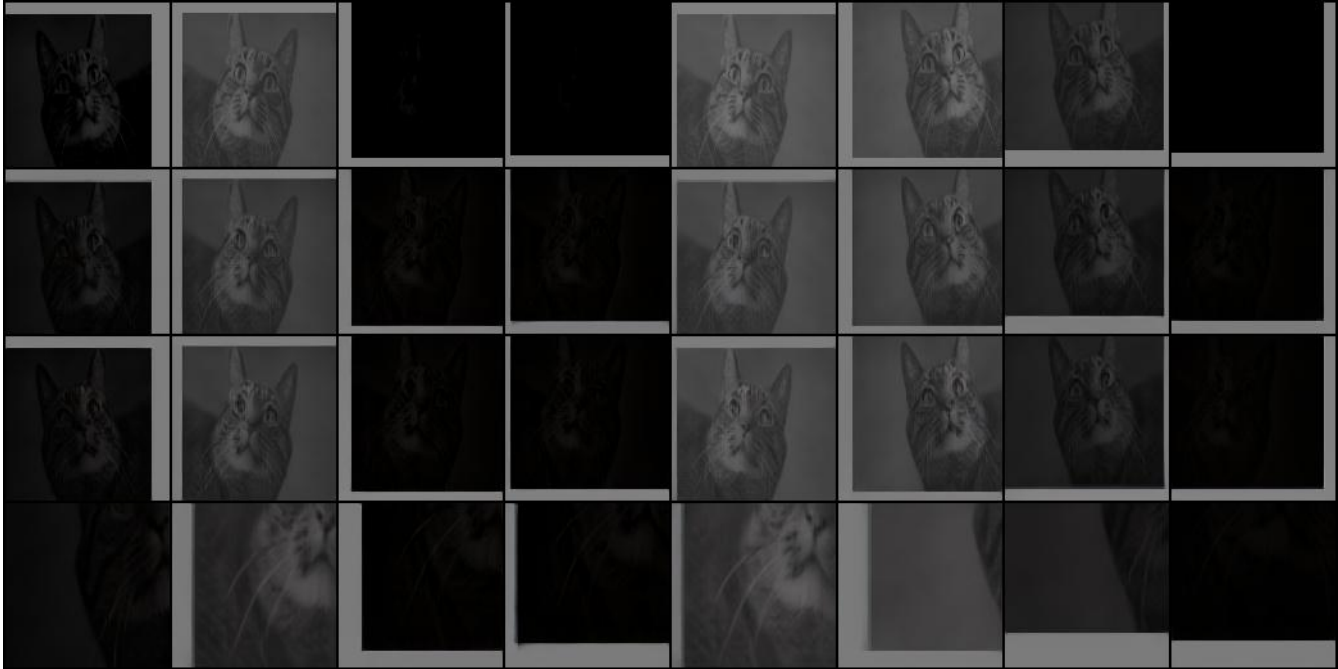

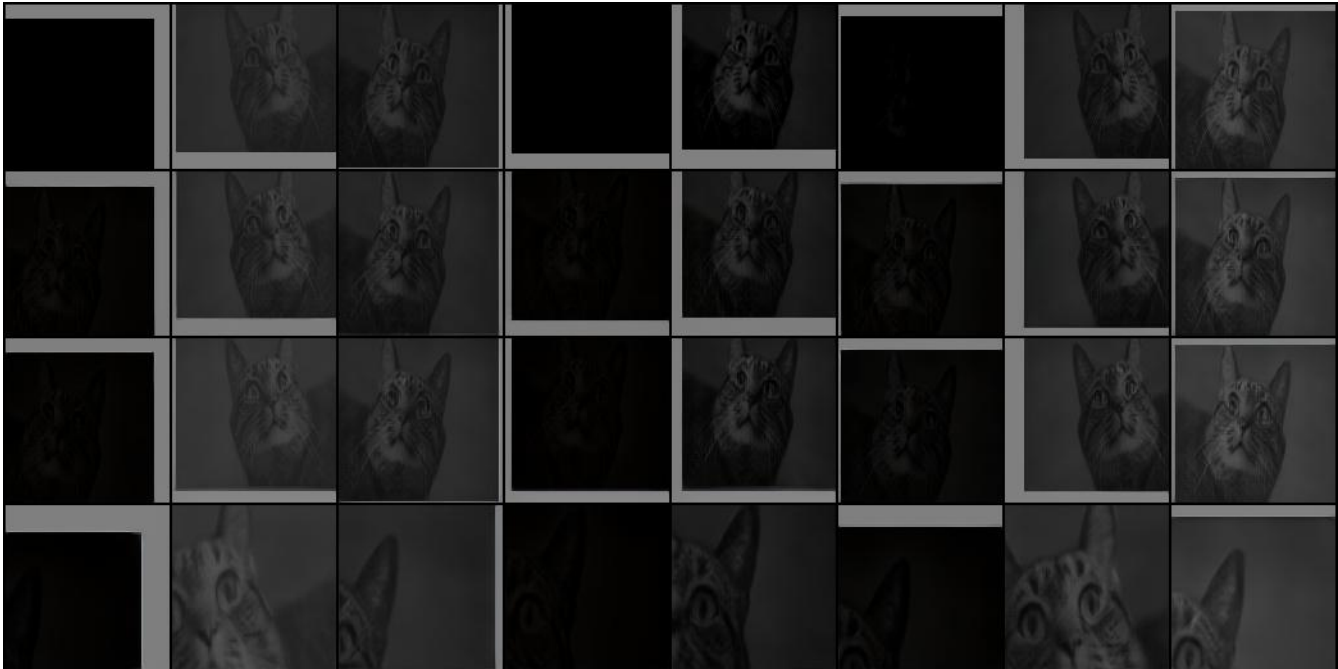

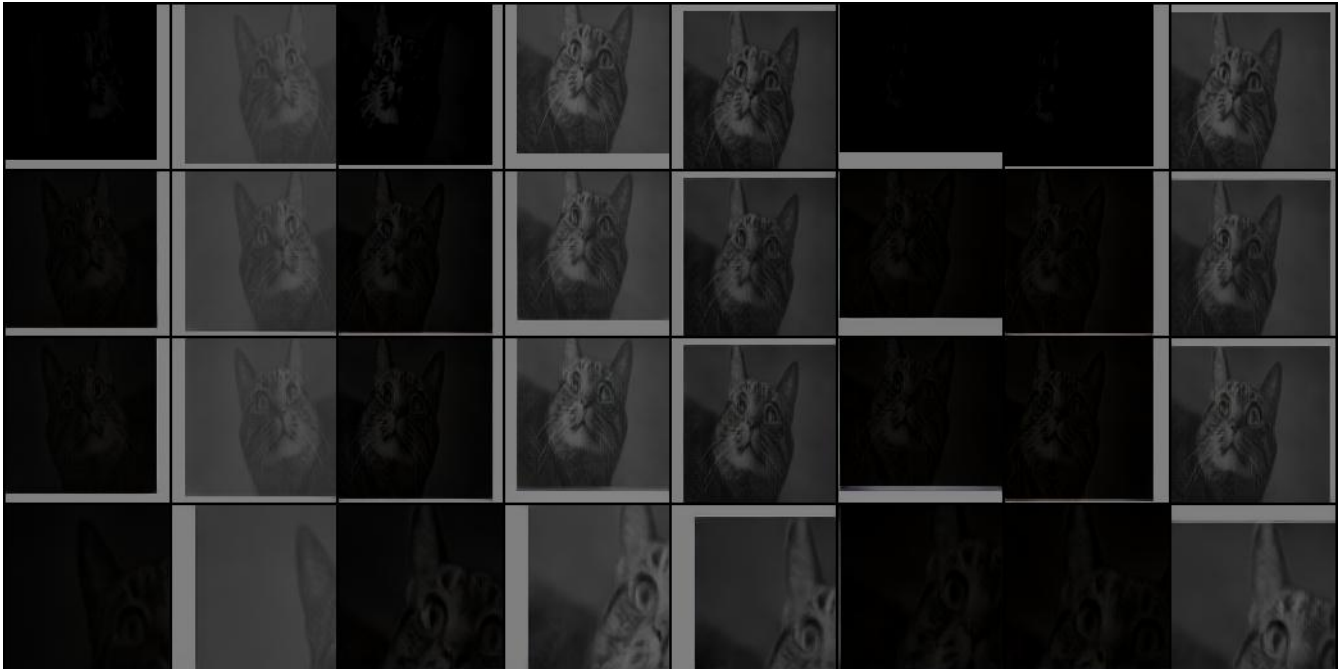

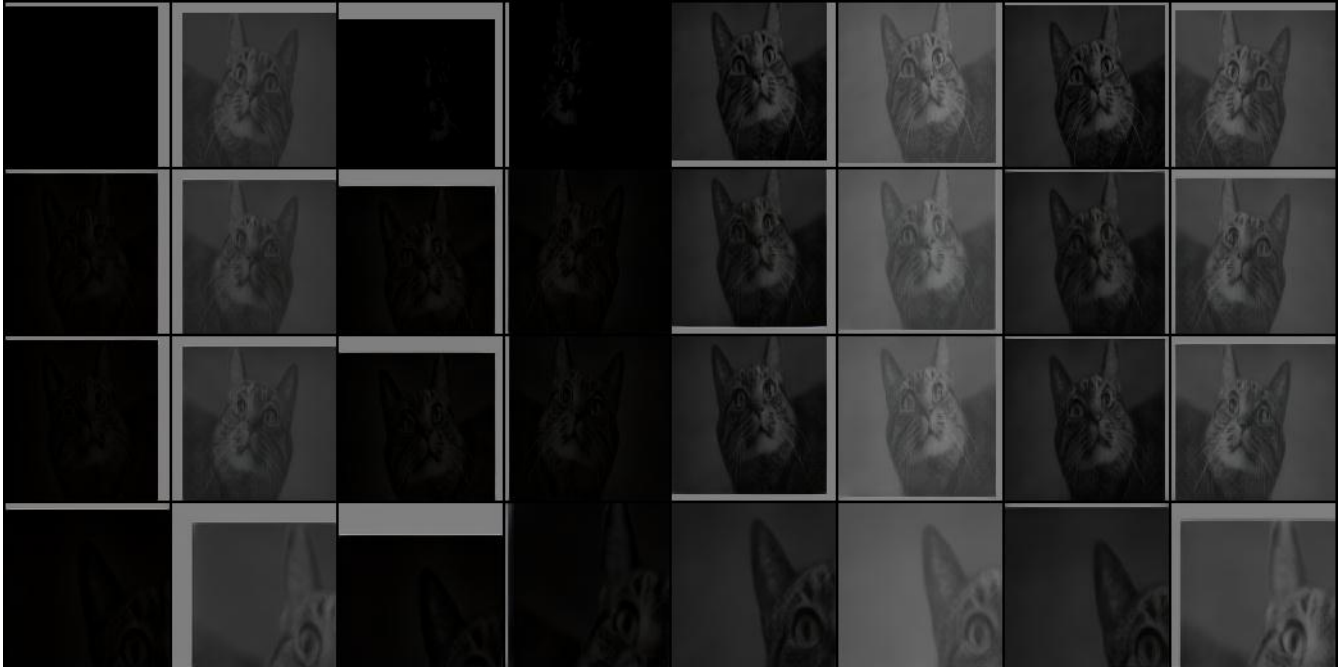

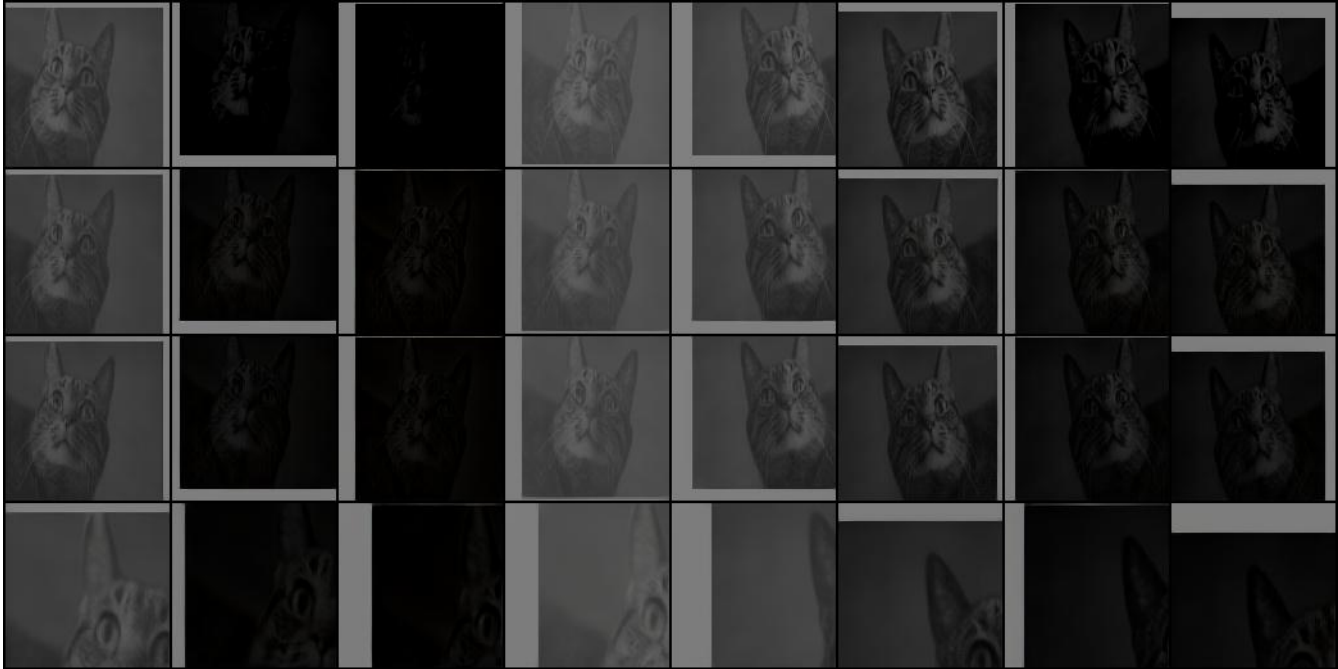

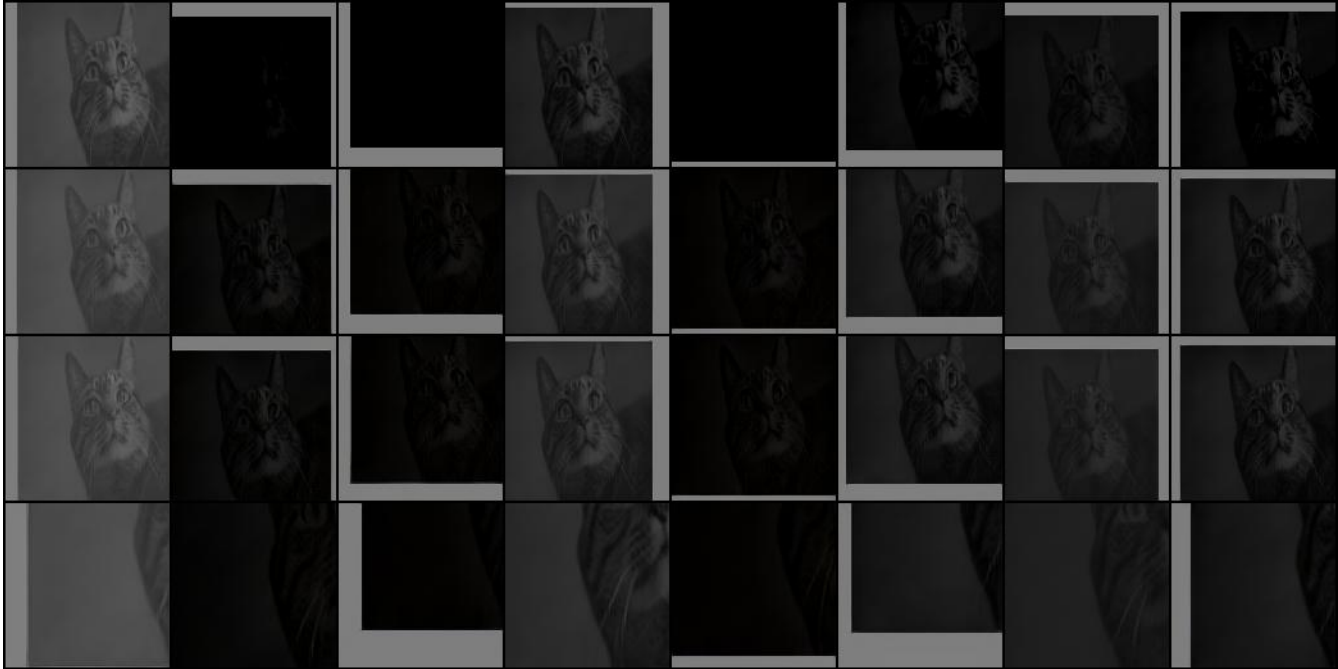

Supplement: S3 Fig — This figure illustrates the generative process under alternative training configurations (e.g., baseline comparison or different noise levels) to demonstrate the consistency of the GAN’s performance. (PDF) [file pone.0338835.s003.pdf]

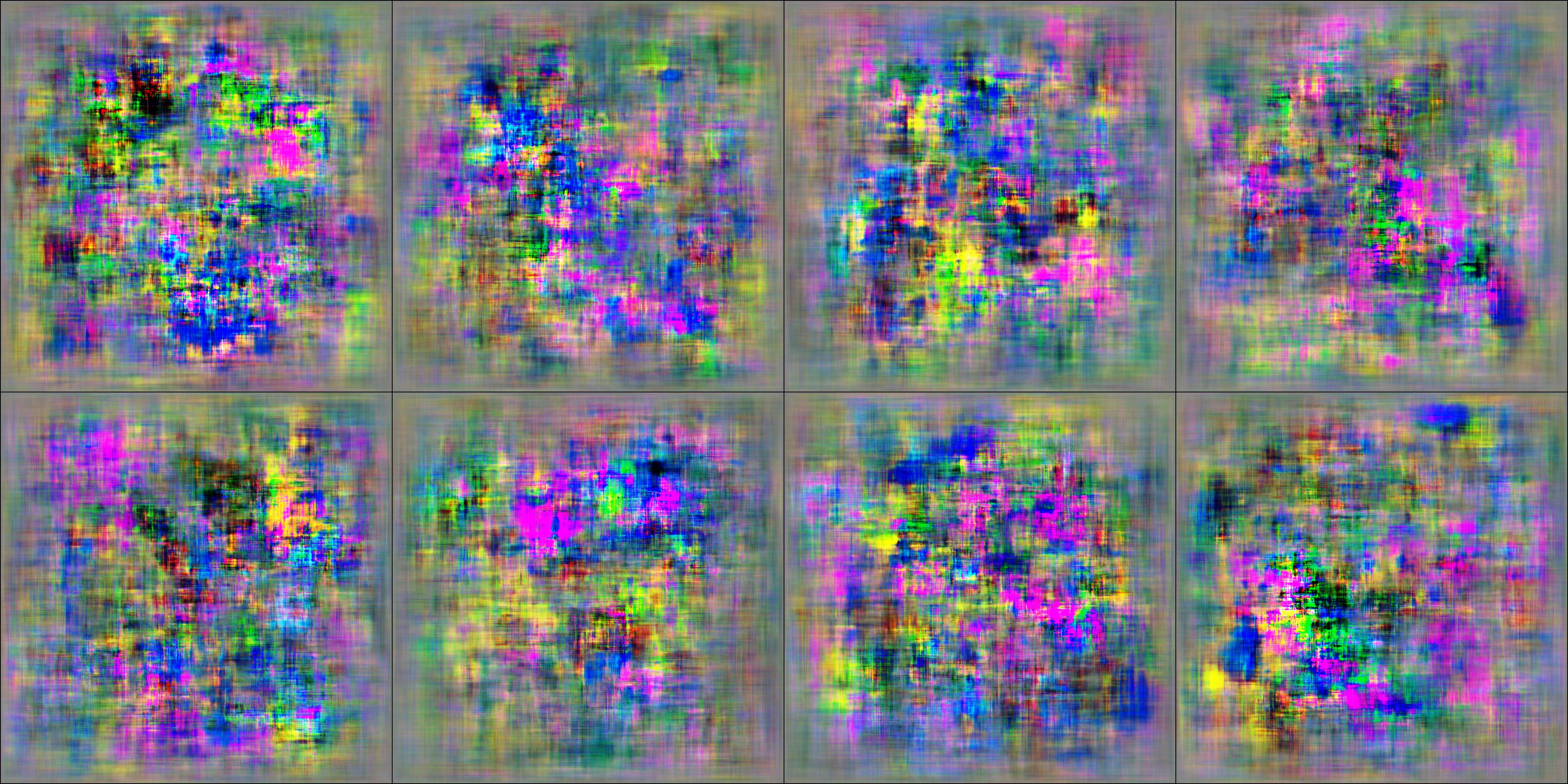

Supplement: S4 File — used in this study, which are essential for reproducing the experimental results and verifying the proposed visual security defense method. (ZIP) [file pone.0338835.s004.zip › image-attacks/0.jpg]

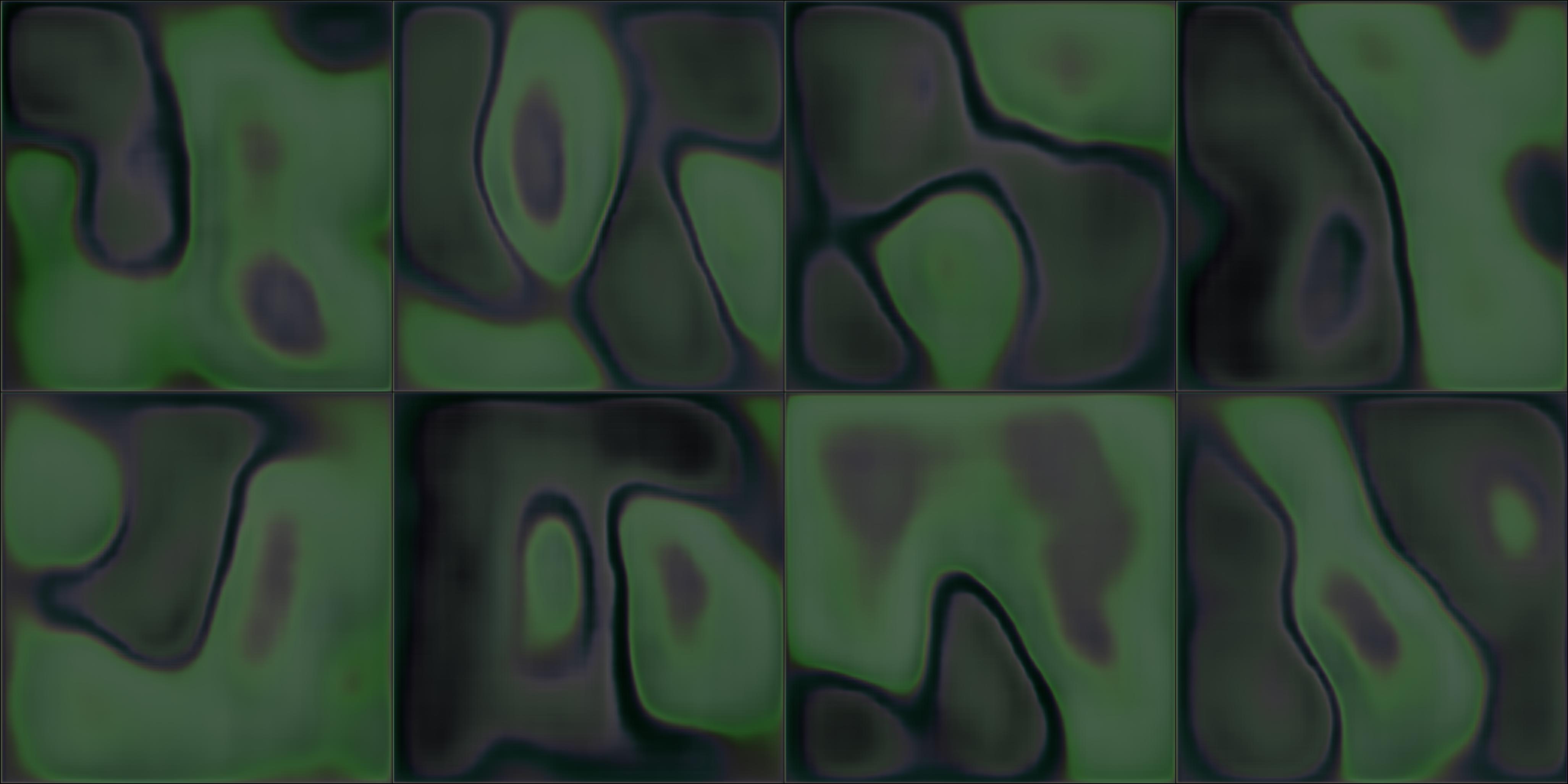

Supplement: S4 File — used in this study, which are essential for reproducing the experimental results and verifying the proposed visual security defense method. (ZIP) [file pone.0338835.s004.zip › image-attacks/1000.jpg]

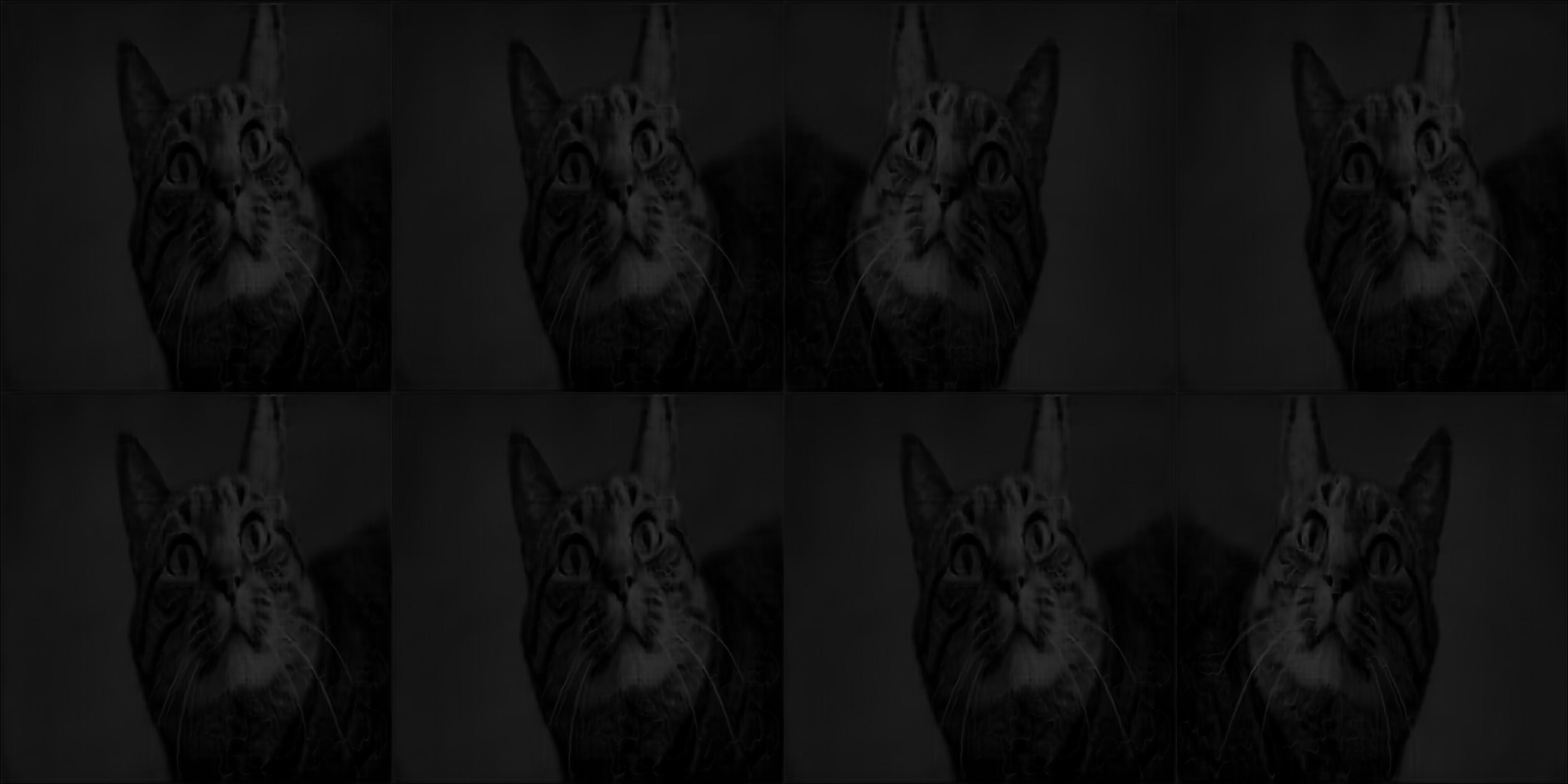

Supplement: S4 File — used in this study, which are essential for reproducing the experimental results and verifying the proposed visual security defense method. (ZIP) [file pone.0338835.s004.zip › image-attacks/10000.jpg]

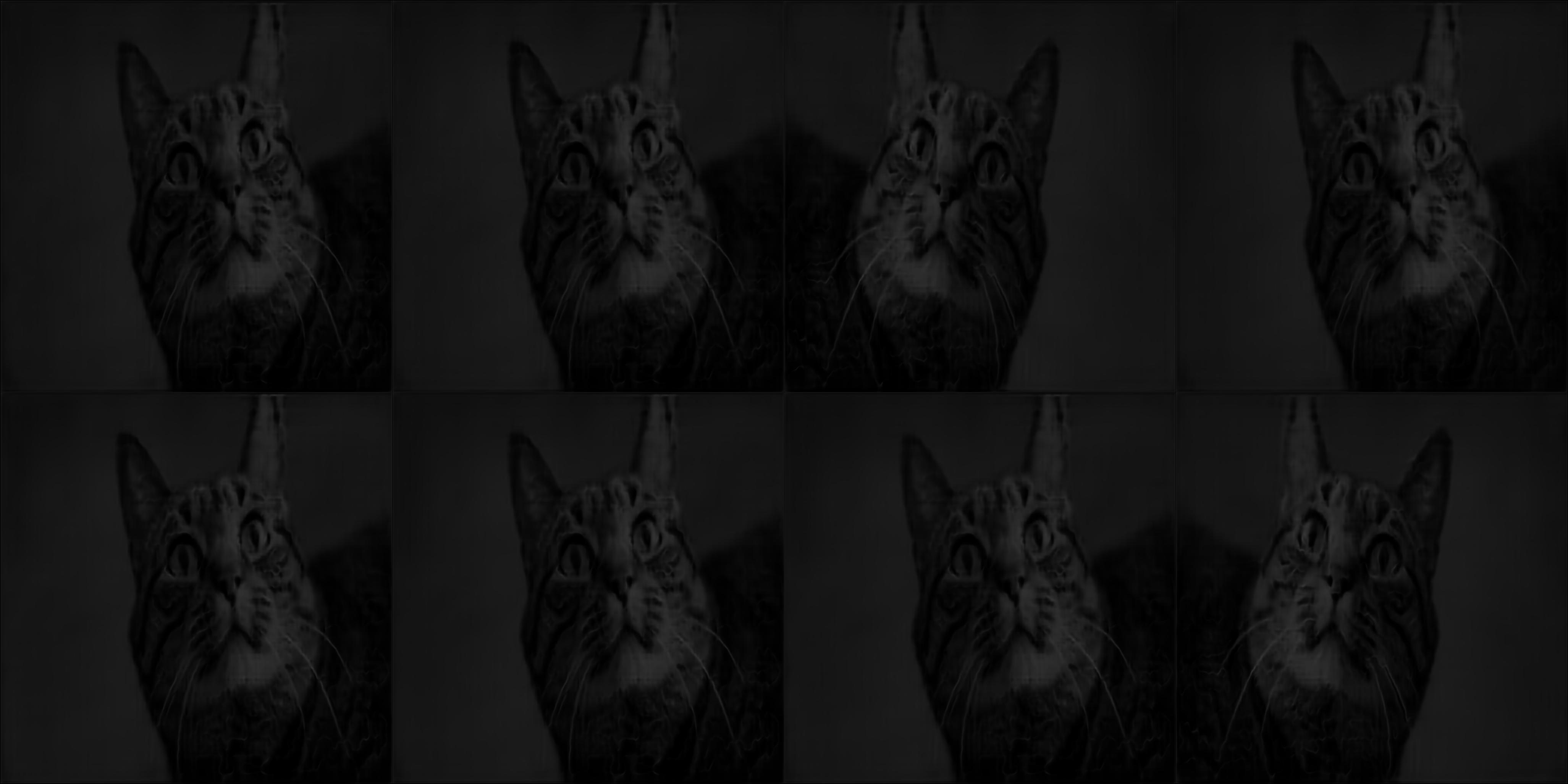

Supplement: S4 File — used in this study, which are essential for reproducing the experimental results and verifying the proposed visual security defense method. (ZIP) [file pone.0338835.s004.zip › image-attacks/11000.jpg]

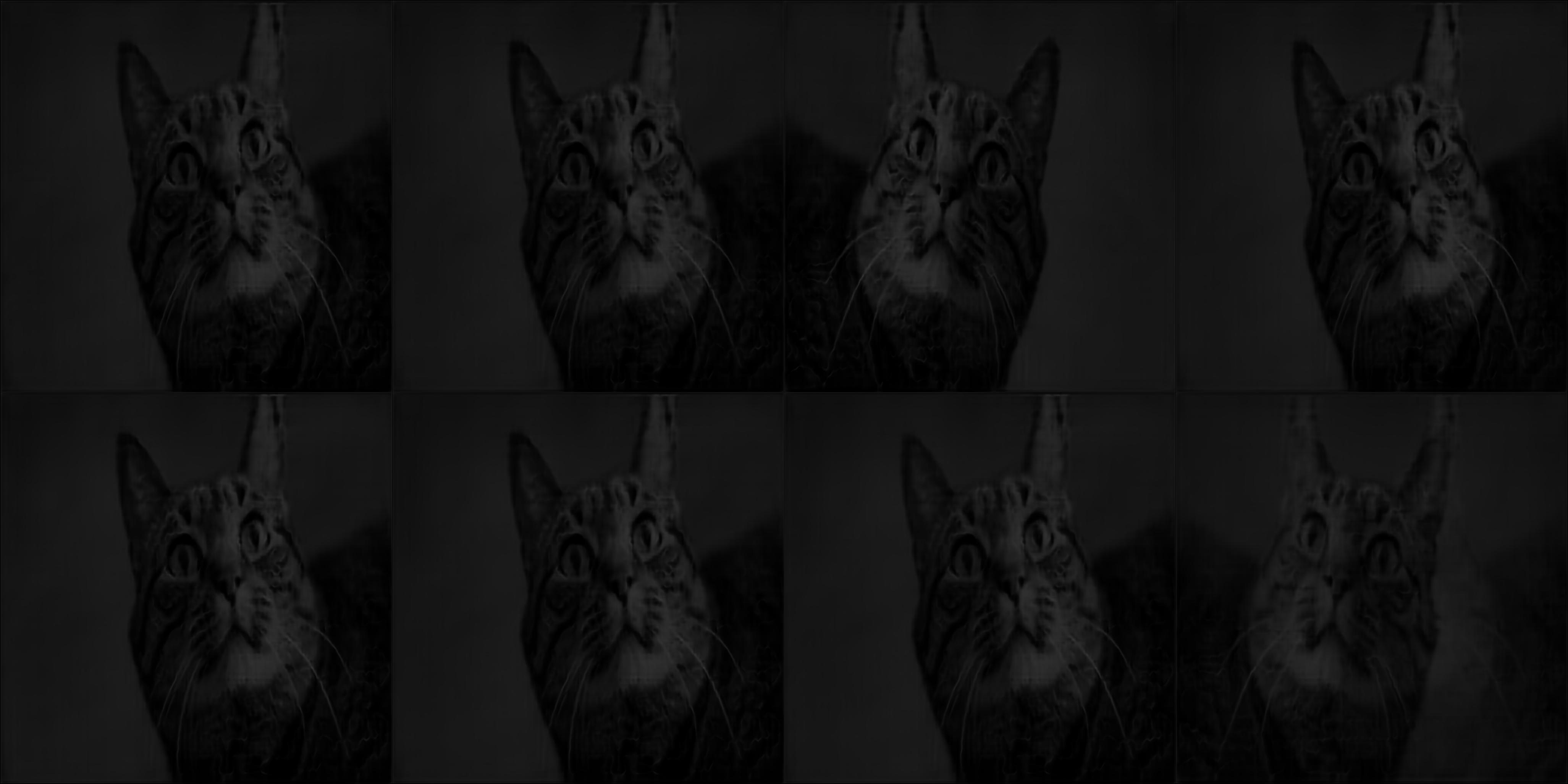

Supplement: S4 File — used in this study, which are essential for reproducing the experimental results and verifying the proposed visual security defense method. (ZIP) [file pone.0338835.s004.zip › image-attacks/12000.jpg]

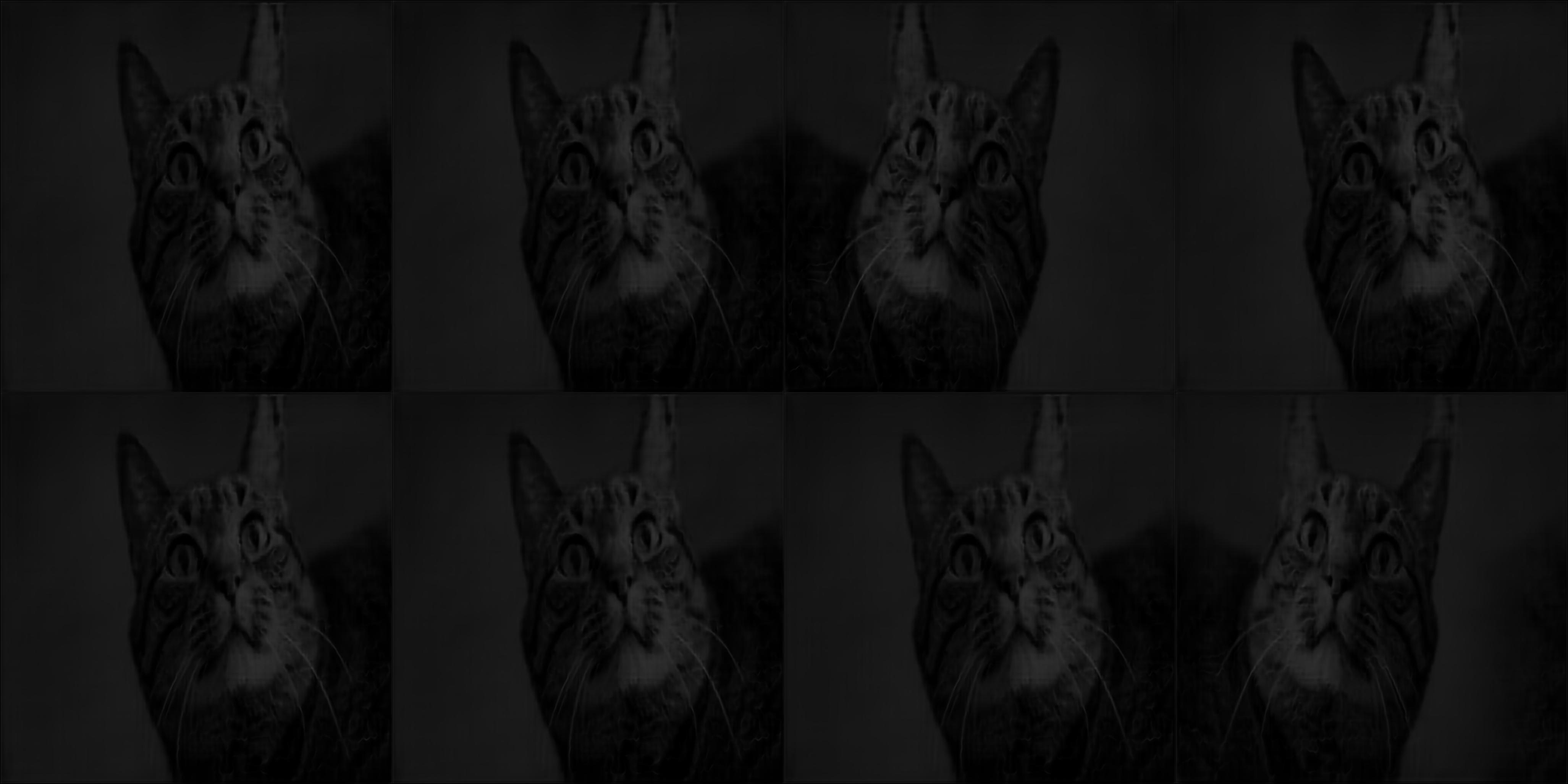

Supplement: S4 File — used in this study, which are essential for reproducing the experimental results and verifying the proposed visual security defense method. (ZIP) [file pone.0338835.s004.zip › image-attacks/13000.jpg]

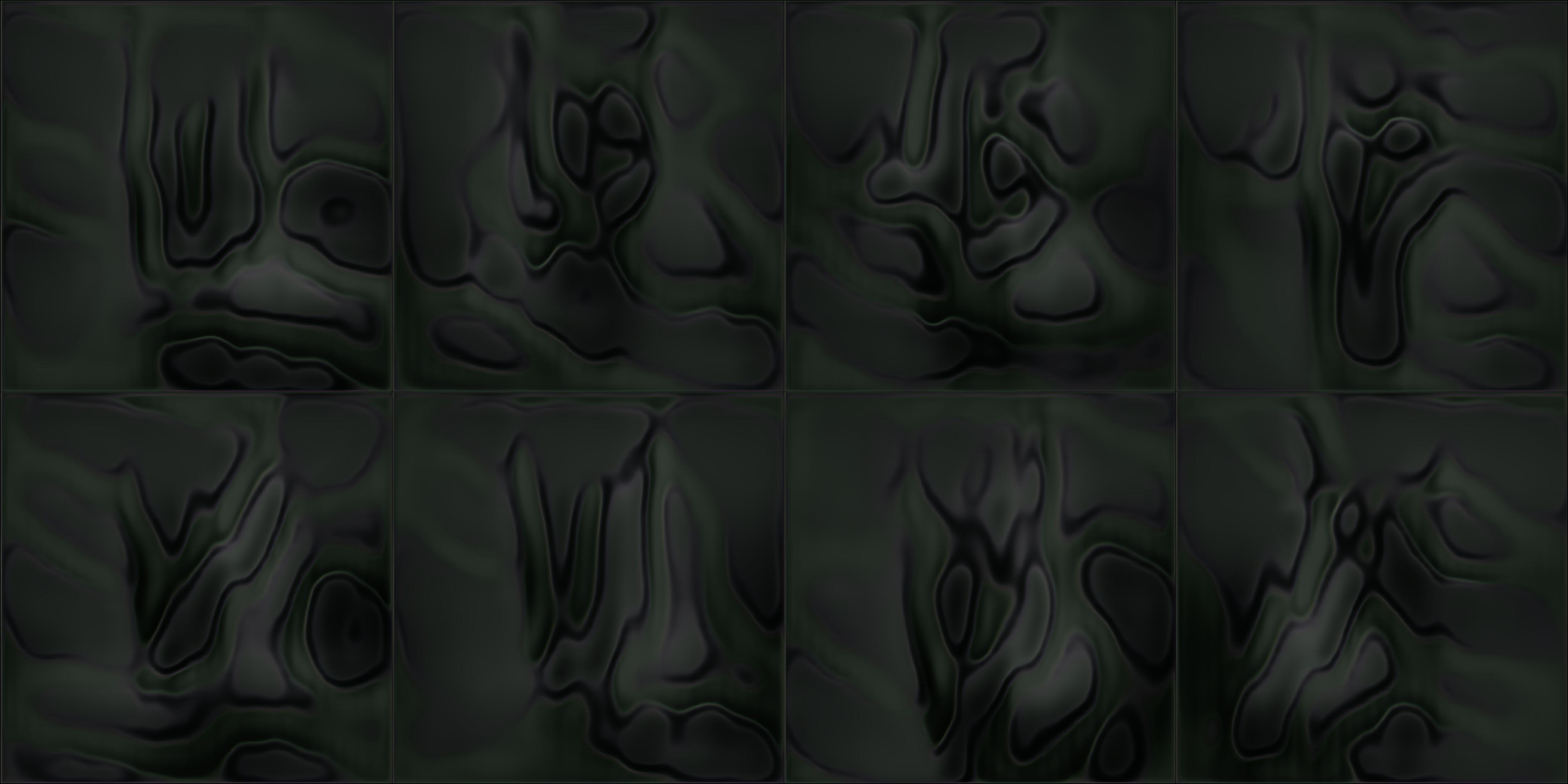

Supplement: S4 File — used in this study, which are essential for reproducing the experimental results and verifying the proposed visual security defense method. (ZIP) [file pone.0338835.s004.zip › image-attacks/2000.jpg]

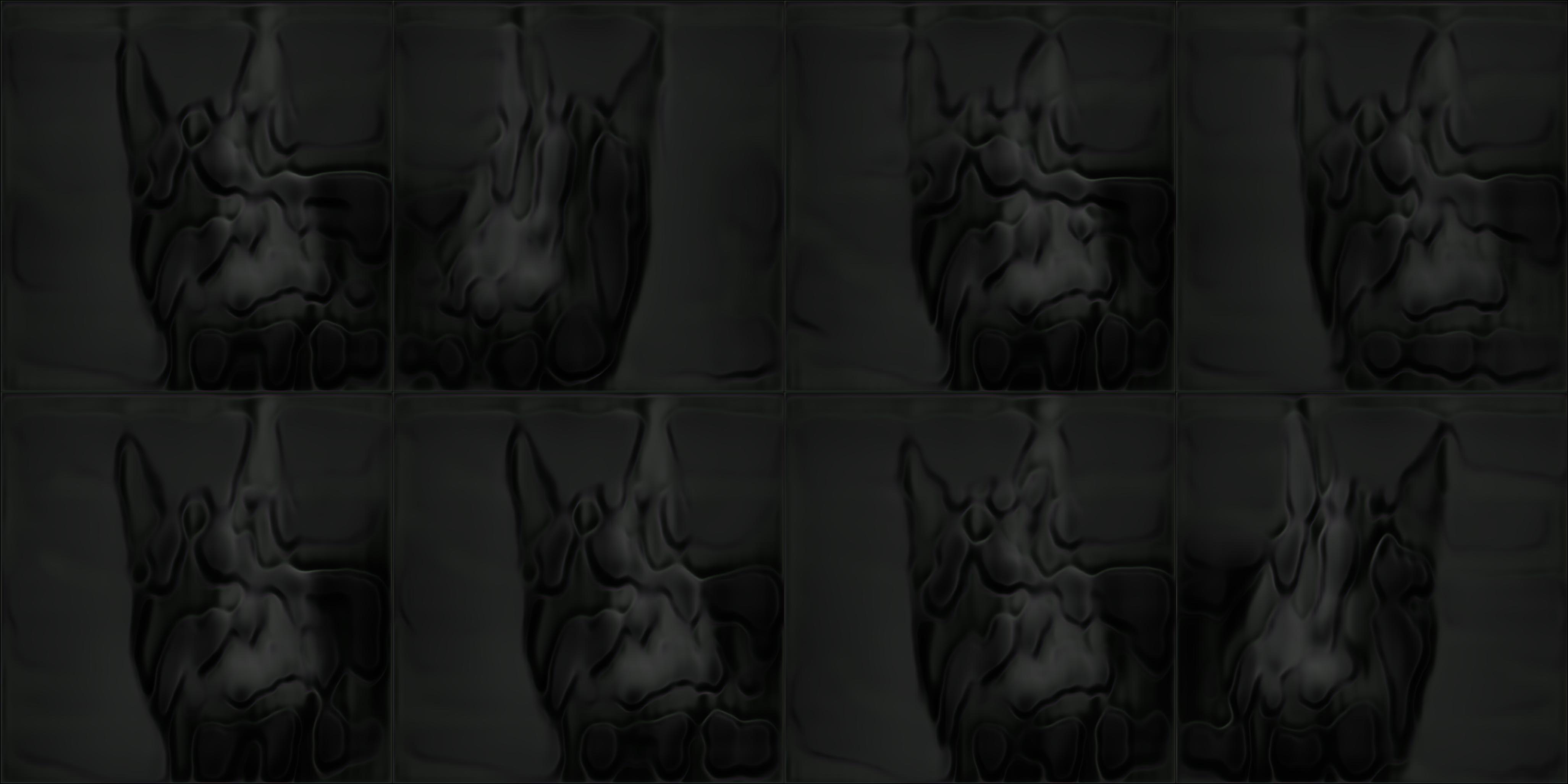

Supplement: S4 File — used in this study, which are essential for reproducing the experimental results and verifying the proposed visual security defense method. (ZIP) [file pone.0338835.s004.zip › image-attacks/3000.jpg]

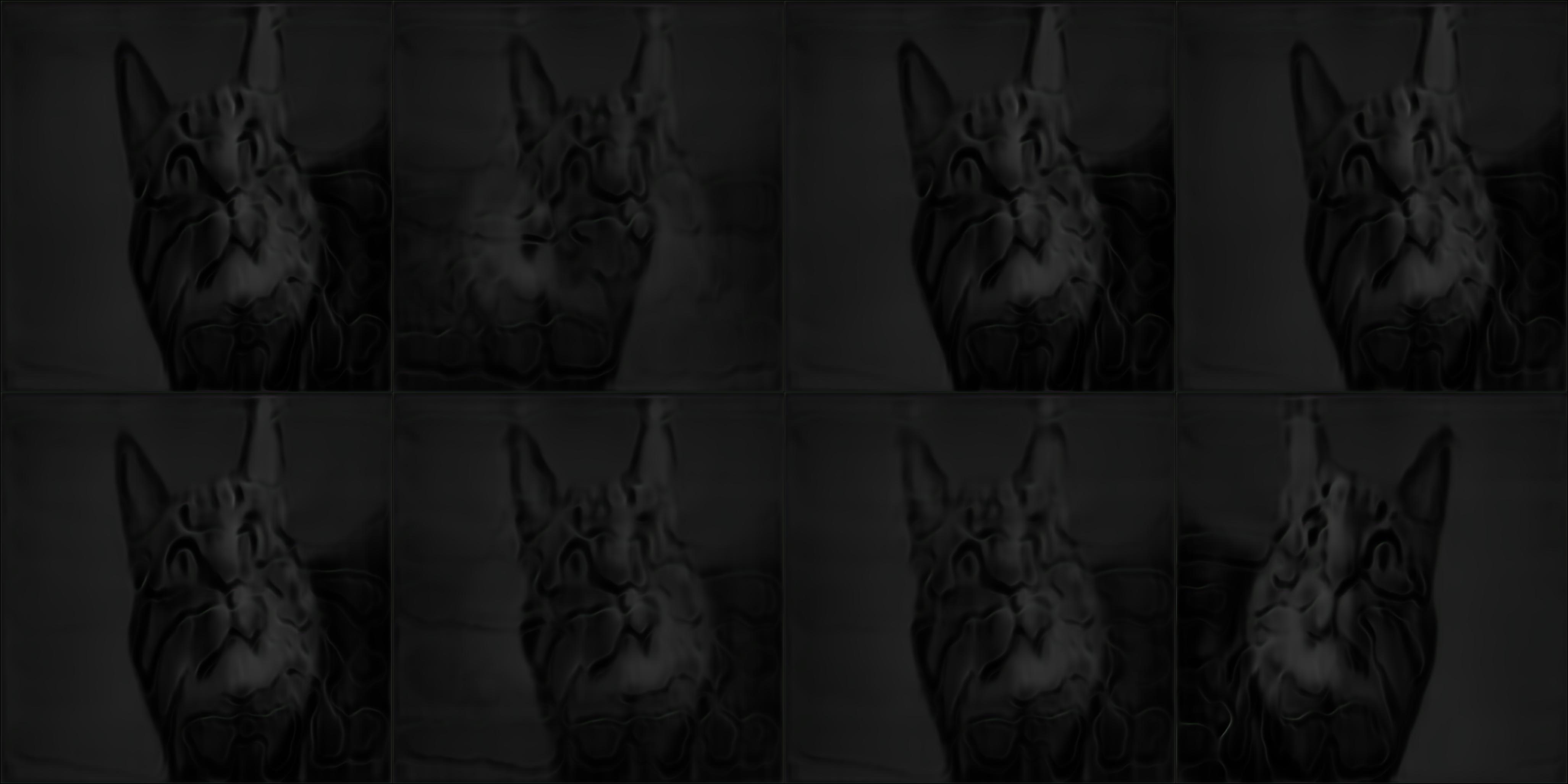

Supplement: S4 File — used in this study, which are essential for reproducing the experimental results and verifying the proposed visual security defense method. (ZIP) [file pone.0338835.s004.zip › image-attacks/4000.jpg]

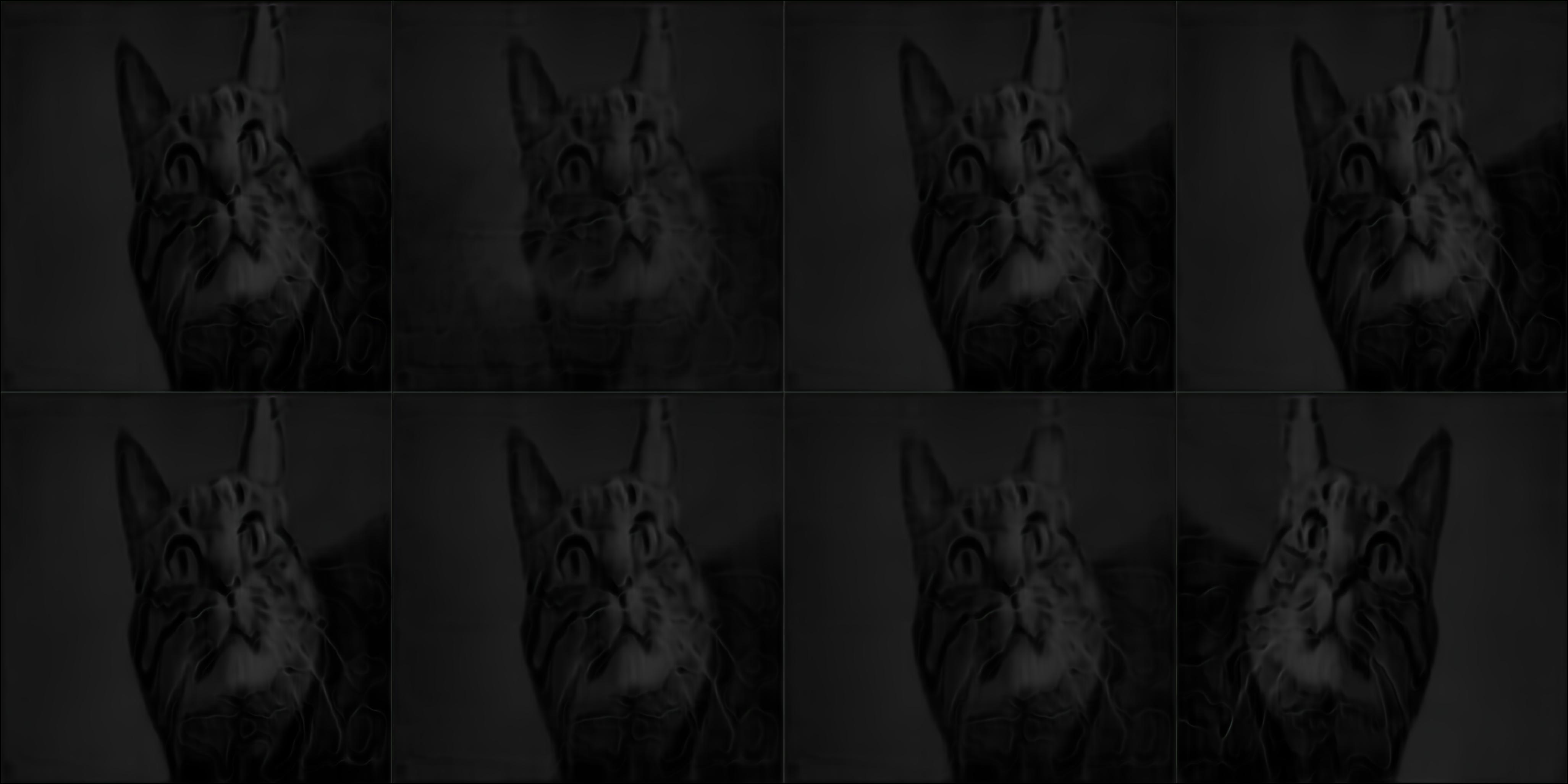

Supplement: S4 File — used in this study, which are essential for reproducing the experimental results and verifying the proposed visual security defense method. (ZIP) [file pone.0338835.s004.zip › image-attacks/5000.jpg]

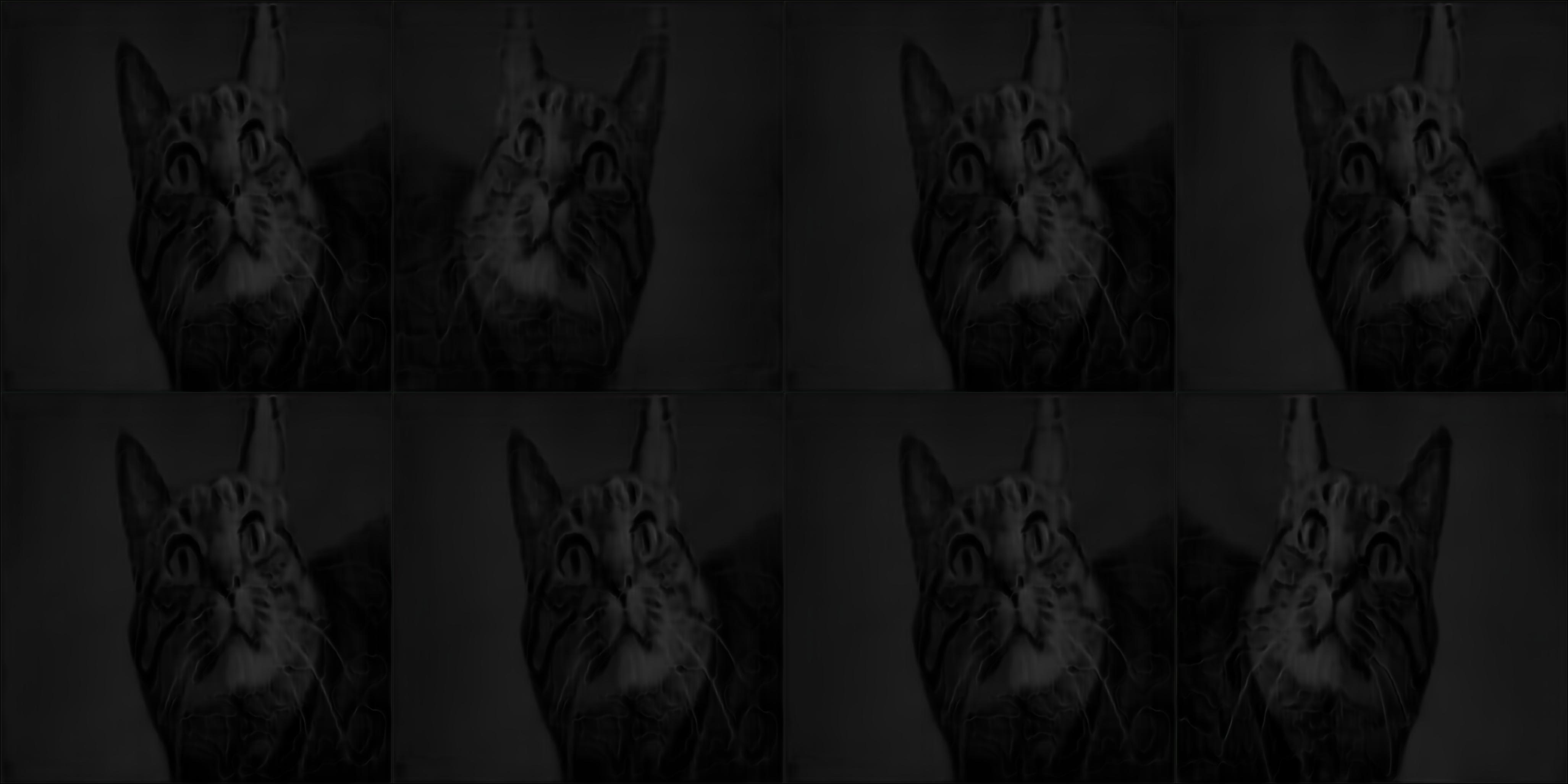

Supplement: S4 File — used in this study, which are essential for reproducing the experimental results and verifying the proposed visual security defense method. (ZIP) [file pone.0338835.s004.zip › image-attacks/6000.jpg]

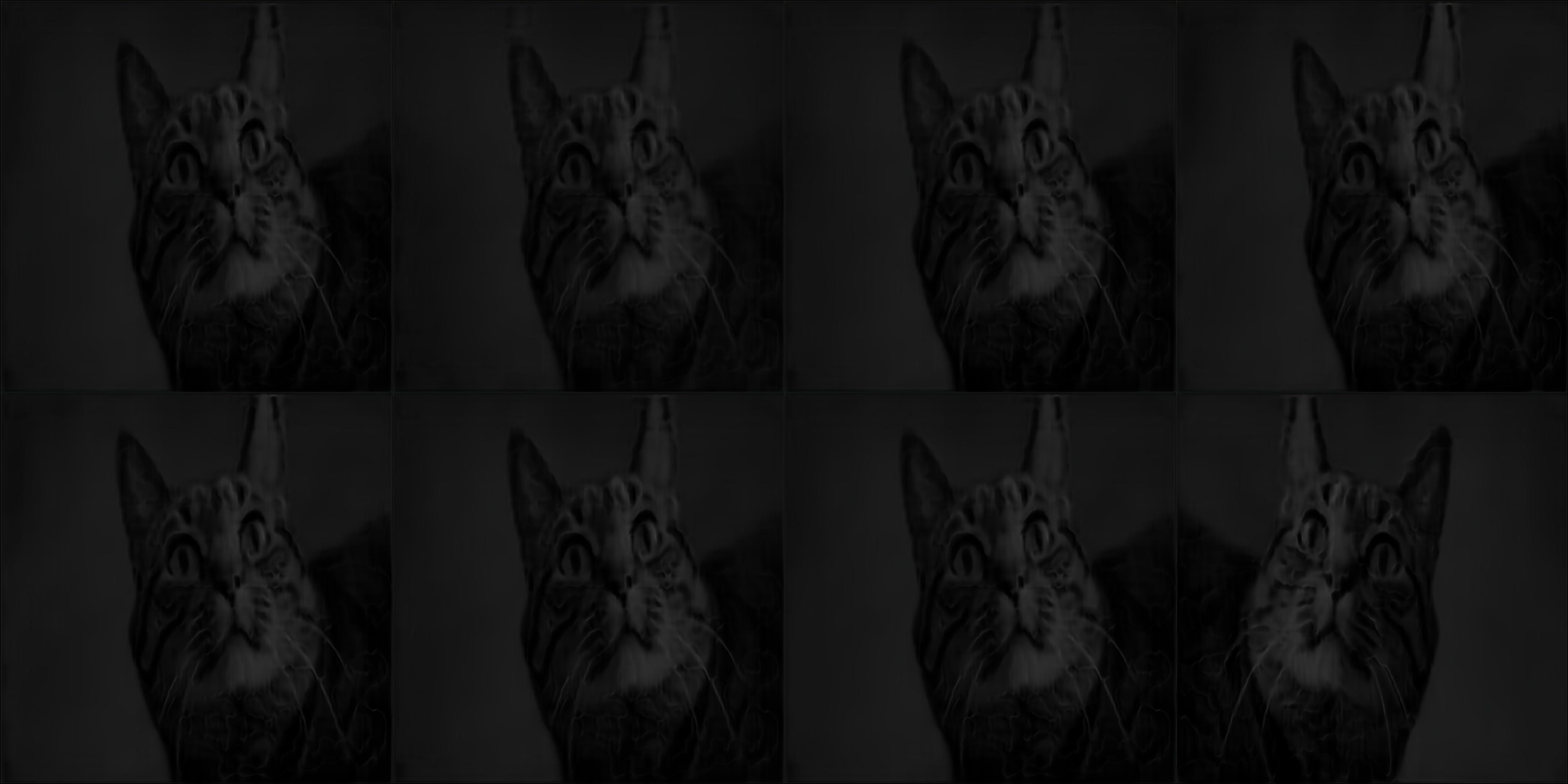

Supplement: S4 File — used in this study, which are essential for reproducing the experimental results and verifying the proposed visual security defense method. (ZIP) [file pone.0338835.s004.zip › image-attacks/7000.jpg]

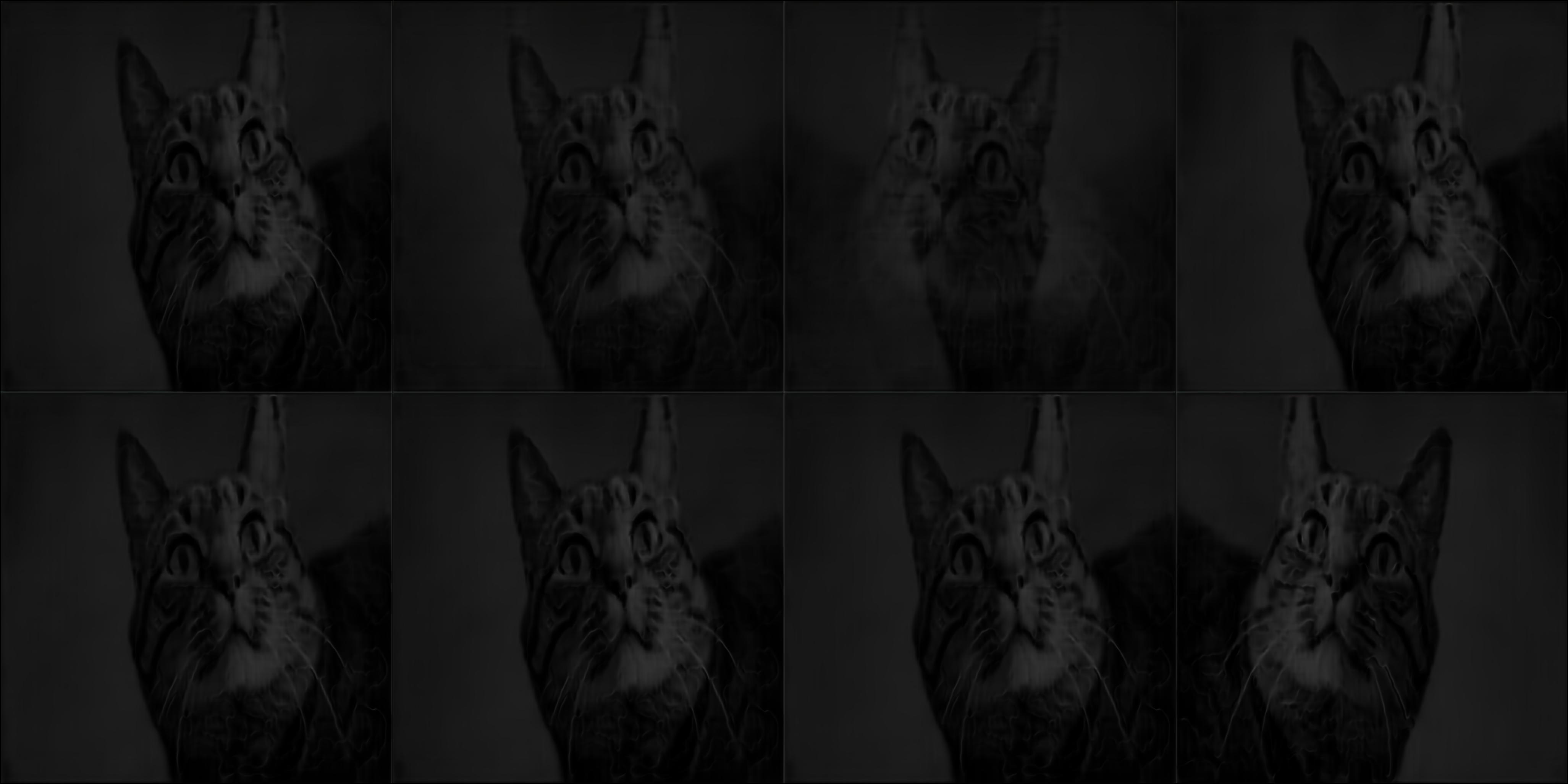

Supplement: S4 File — used in this study, which are essential for reproducing the experimental results and verifying the proposed visual security defense method. (ZIP) [file pone.0338835.s004.zip › image-attacks/8000.jpg]

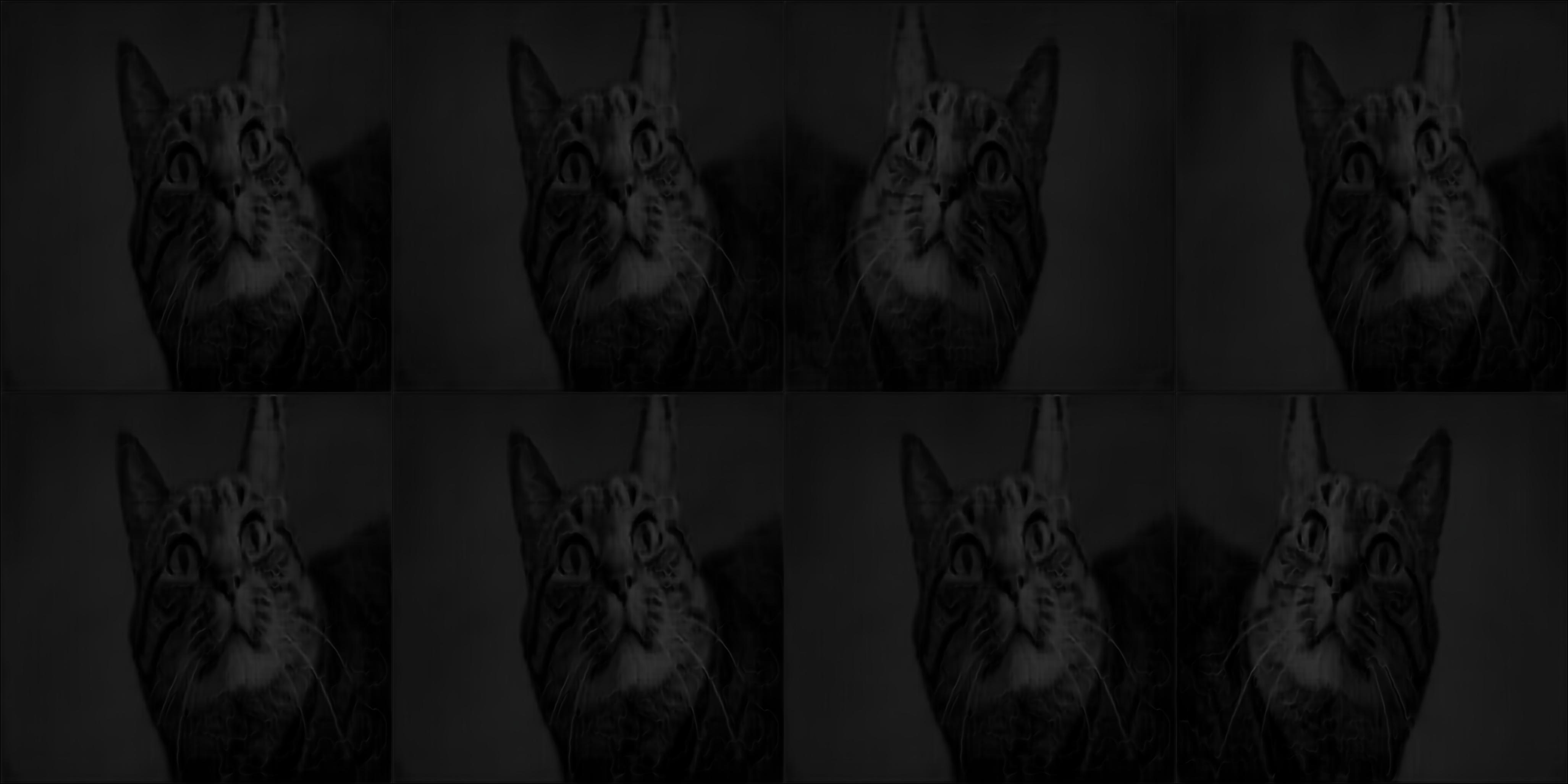

Supplement: S4 File — used in this study, which are essential for reproducing the experimental results and verifying the proposed visual security defense method. (ZIP) [file pone.0338835.s004.zip › image-attacks/9000.jpg]

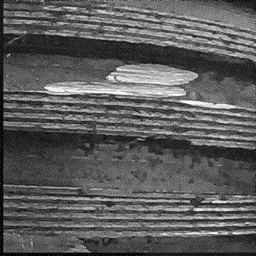

Supplement: S4 File — used in this study, which are essential for reproducing the experimental results and verifying the proposed visual security defense method. (ZIP) [file pone.0338835.s004.zip › image-attacks/attack_10.png]

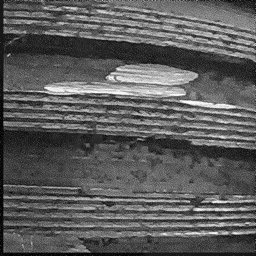

Supplement: S4 File — used in this study, which are essential for reproducing the experimental results and verifying the proposed visual security defense method. (ZIP) [file pone.0338835.s004.zip › image-attacks/attack_100.png]

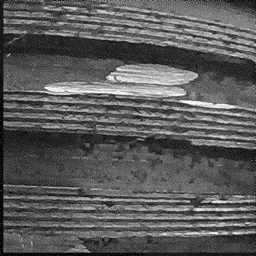

Supplement: S4 File — used in this study, which are essential for reproducing the experimental results and verifying the proposed visual security defense method. (ZIP) [file pone.0338835.s004.zip › image-attacks/attack_11.png]

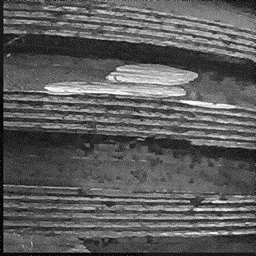

Supplement: S4 File — used in this study, which are essential for reproducing the experimental results and verifying the proposed visual security defense method. (ZIP) [file pone.0338835.s004.zip › image-attacks/attack_12.png]

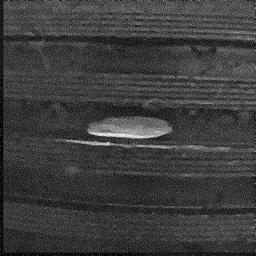

Supplement: S4 File — used in this study, which are essential for reproducing the experimental results and verifying the proposed visual security defense method. (ZIP) [file pone.0338835.s004.zip › image-attacks/attack_13.png]

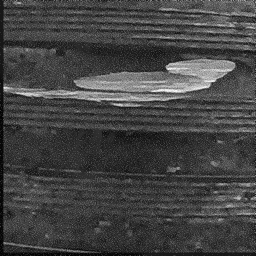

Supplement: S4 File — used in this study, which are essential for reproducing the experimental results and verifying the proposed visual security defense method. (ZIP) [file pone.0338835.s004.zip › image-attacks/attack_14.png]

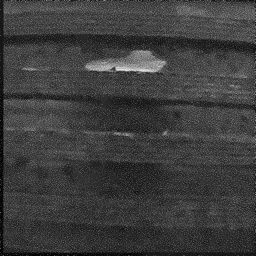

Supplement: S4 File — used in this study, which are essential for reproducing the experimental results and verifying the proposed visual security defense method. (ZIP) [file pone.0338835.s004.zip › image-attacks/attack_15.png]

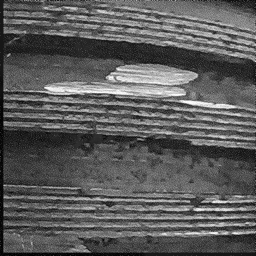

Supplement: S4 File — used in this study, which are essential for reproducing the experimental results and verifying the proposed visual security defense method. (ZIP) [file pone.0338835.s004.zip › image-attacks/attack_16.png]

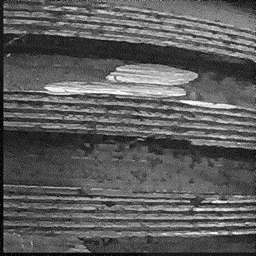

Supplement: S4 File — used in this study, which are essential for reproducing the experimental results and verifying the proposed visual security defense method. (ZIP) [file pone.0338835.s004.zip › image-attacks/attack_17.png]

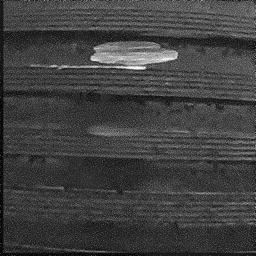

Supplement: S4 File — used in this study, which are essential for reproducing the experimental results and verifying the proposed visual security defense method. (ZIP) [file pone.0338835.s004.zip › image-attacks/attack_18.png]

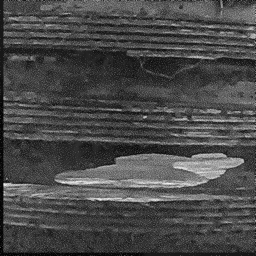

Supplement: S4 File — used in this study, which are essential for reproducing the experimental results and verifying the proposed visual security defense method. (ZIP) [file pone.0338835.s004.zip › image-attacks/attack_19.png]

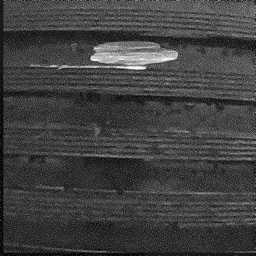

Supplement: S4 File — used in this study, which are essential for reproducing the experimental results and verifying the proposed visual security defense method. (ZIP) [file pone.0338835.s004.zip › image-attacks/attack_20.png]

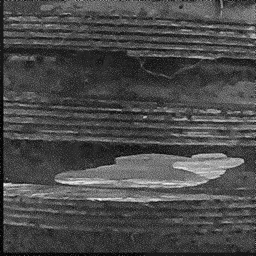

Supplement: S4 File — used in this study, which are essential for reproducing the experimental results and verifying the proposed visual security defense method. (ZIP) [file pone.0338835.s004.zip › image-attacks/attack_21.png]

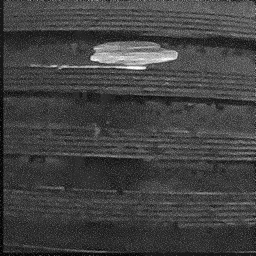

Supplement: S4 File — used in this study, which are essential for reproducing the experimental results and verifying the proposed visual security defense method. (ZIP) [file pone.0338835.s004.zip › image-attacks/attack_22.png]

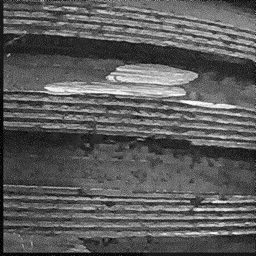

Supplement: S4 File — used in this study, which are essential for reproducing the experimental results and verifying the proposed visual security defense method. (ZIP) [file pone.0338835.s004.zip › image-attacks/attack_23.png]

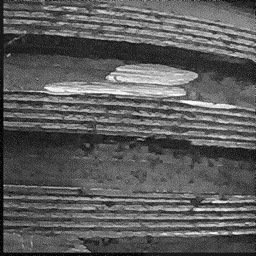

Supplement: S4 File — used in this study, which are essential for reproducing the experimental results and verifying the proposed visual security defense method. (ZIP) [file pone.0338835.s004.zip › image-attacks/attack_24.png]

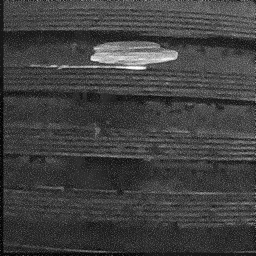

Supplement: S4 File — used in this study, which are essential for reproducing the experimental results and verifying the proposed visual security defense method. (ZIP) [file pone.0338835.s004.zip › image-attacks/attack_25.png]

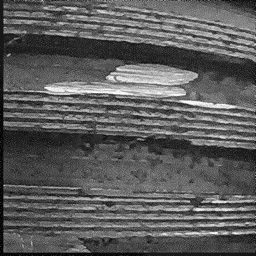

Supplement: S4 File — used in this study, which are essential for reproducing the experimental results and verifying the proposed visual security defense method. (ZIP) [file pone.0338835.s004.zip › image-attacks/attack_26.png]

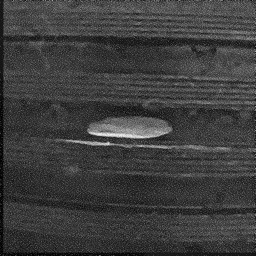

Supplement: S4 File — used in this study, which are essential for reproducing the experimental results and verifying the proposed visual security defense method. (ZIP) [file pone.0338835.s004.zip › image-attacks/attack_27.png]

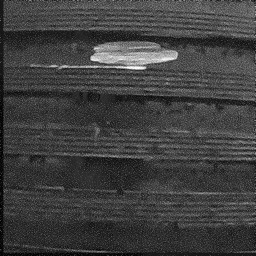

Supplement: S4 File — used in this study, which are essential for reproducing the experimental results and verifying the proposed visual security defense method. (ZIP) [file pone.0338835.s004.zip › image-attacks/attack_28.png]

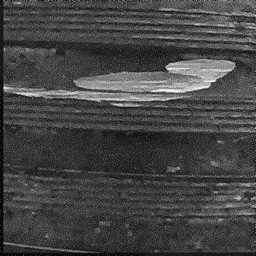

Supplement: S4 File — used in this study, which are essential for reproducing the experimental results and verifying the proposed visual security defense method. (ZIP) [file pone.0338835.s004.zip › image-attacks/attack_29.png]

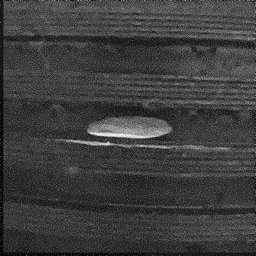

Supplement: S4 File — used in this study, which are essential for reproducing the experimental results and verifying the proposed visual security defense method. (ZIP) [file pone.0338835.s004.zip › image-attacks/attack_3.png]

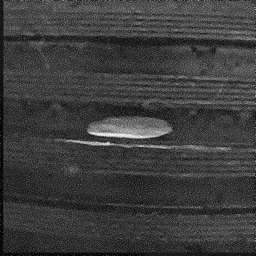

Supplement: S4 File — used in this study, which are essential for reproducing the experimental results and verifying the proposed visual security defense method. (ZIP) [file pone.0338835.s004.zip › image-attacks/attack_30.png]

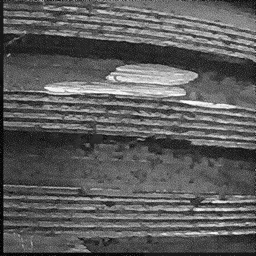

Supplement: S4 File — used in this study, which are essential for reproducing the experimental results and verifying the proposed visual security defense method. (ZIP) [file pone.0338835.s004.zip › image-attacks/attack_31.png]

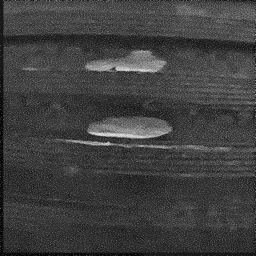

Supplement: S4 File — used in this study, which are essential for reproducing the experimental results and verifying the proposed visual security defense method. (ZIP) [file pone.0338835.s004.zip › image-attacks/attack_32.png]

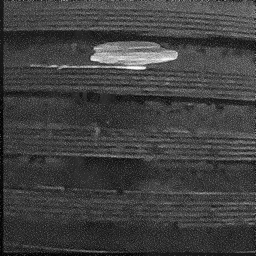

Supplement: S4 File — used in this study, which are essential for reproducing the experimental results and verifying the proposed visual security defense method. (ZIP) [file pone.0338835.s004.zip › image-attacks/attack_33.png]

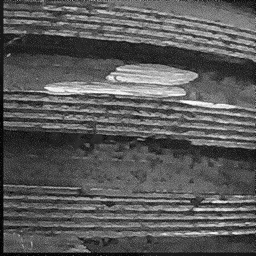

Supplement: S4 File — used in this study, which are essential for reproducing the experimental results and verifying the proposed visual security defense method. (ZIP) [file pone.0338835.s004.zip › image-attacks/attack_34.png]

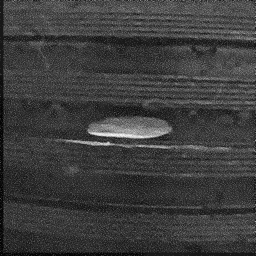

Supplement: S4 File — used in this study, which are essential for reproducing the experimental results and verifying the proposed visual security defense method. (ZIP) [file pone.0338835.s004.zip › image-attacks/attack_35.png]

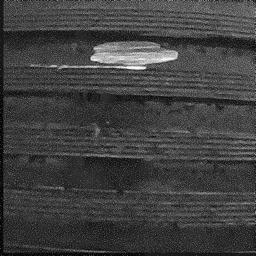

Supplement: S4 File — used in this study, which are essential for reproducing the experimental results and verifying the proposed visual security defense method. (ZIP) [file pone.0338835.s004.zip › image-attacks/attack_36.png]

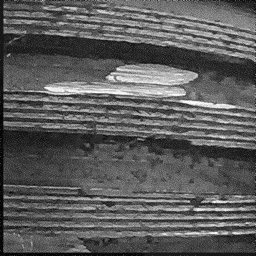

Supplement: S4 File — used in this study, which are essential for reproducing the experimental results and verifying the proposed visual security defense method. (ZIP) [file pone.0338835.s004.zip › image-attacks/attack_37.png]

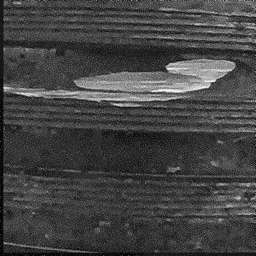

Supplement: S4 File — used in this study, which are essential for reproducing the experimental results and verifying the proposed visual security defense method. (ZIP) [file pone.0338835.s004.zip › image-attacks/attack_38.png]

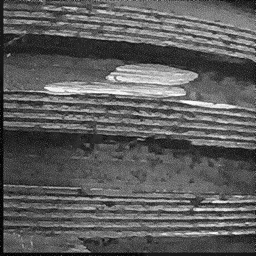

Supplement: S4 File — used in this study, which are essential for reproducing the experimental results and verifying the proposed visual security defense method. (ZIP) [file pone.0338835.s004.zip › image-attacks/attack_39.png]

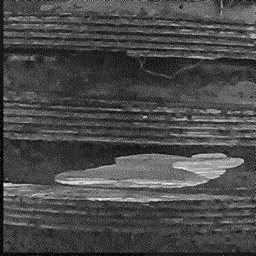

Supplement: S4 File — used in this study, which are essential for reproducing the experimental results and verifying the proposed visual security defense method. (ZIP) [file pone.0338835.s004.zip › image-attacks/attack_4.png]

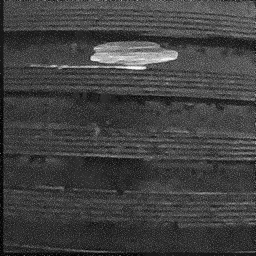

Supplement: S4 File — used in this study, which are essential for reproducing the experimental results and verifying the proposed visual security defense method. (ZIP) [file pone.0338835.s004.zip › image-attacks/attack_40.png]

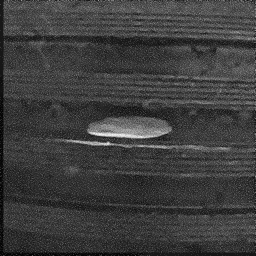

Supplement: S4 File — used in this study, which are essential for reproducing the experimental results and verifying the proposed visual security defense method. (ZIP) [file pone.0338835.s004.zip › image-attacks/attack_41.png]

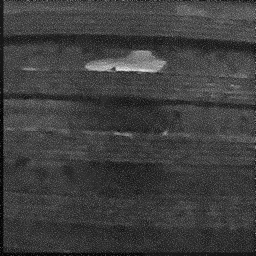

Supplement: S4 File — used in this study, which are essential for reproducing the experimental results and verifying the proposed visual security defense method. (ZIP) [file pone.0338835.s004.zip › image-attacks/attack_42.png]

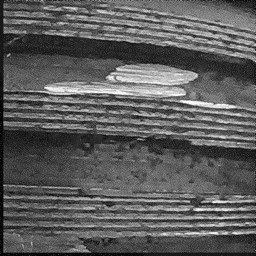

Supplement: S4 File — used in this study, which are essential for reproducing the experimental results and verifying the proposed visual security defense method. (ZIP) [file pone.0338835.s004.zip › image-attacks/attack_43.png]

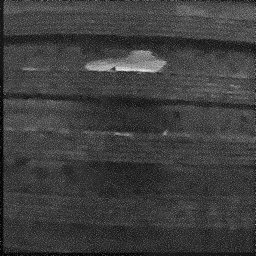

Supplement: S4 File — used in this study, which are essential for reproducing the experimental results and verifying the proposed visual security defense method. (ZIP) [file pone.0338835.s004.zip › image-attacks/attack_44.png]

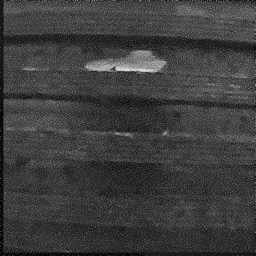

Supplement: S4 File — used in this study, which are essential for reproducing the experimental results and verifying the proposed visual security defense method. (ZIP) [file pone.0338835.s004.zip › image-attacks/attack_45.png]

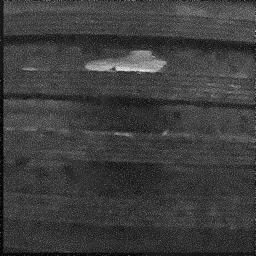

Supplement: S4 File — used in this study, which are essential for reproducing the experimental results and verifying the proposed visual security defense method. (ZIP) [file pone.0338835.s004.zip › image-attacks/attack_46.png]

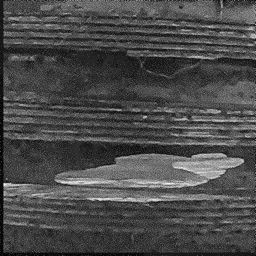

Supplement: S4 File — used in this study, which are essential for reproducing the experimental results and verifying the proposed visual security defense method. (ZIP) [file pone.0338835.s004.zip › image-attacks/attack_47.png]

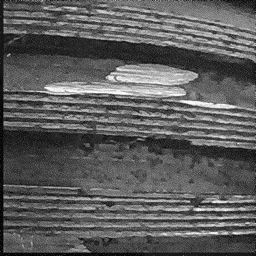

Supplement: S4 File — used in this study, which are essential for reproducing the experimental results and verifying the proposed visual security defense method. (ZIP) [file pone.0338835.s004.zip › image-attacks/attack_48.png]

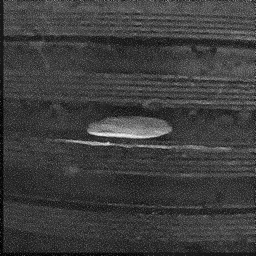

Supplement: S4 File — used in this study, which are essential for reproducing the experimental results and verifying the proposed visual security defense method. (ZIP) [file pone.0338835.s004.zip › image-attacks/attack_49.png]

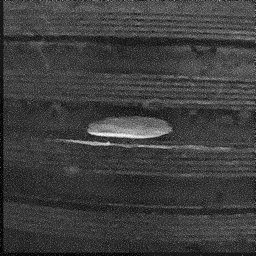

Supplement: S4 File — used in this study, which are essential for reproducing the experimental results and verifying the proposed visual security defense method. (ZIP) [file pone.0338835.s004.zip › image-attacks/attack_5.png]

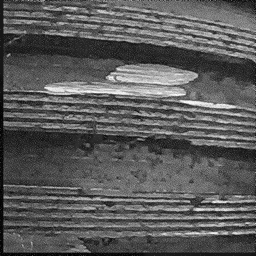

Supplement: S4 File — used in this study, which are essential for reproducing the experimental results and verifying the proposed visual security defense method. (ZIP) [file pone.0338835.s004.zip › image-attacks/attack_50.png]

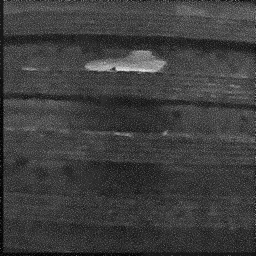

Supplement: S4 File — used in this study, which are essential for reproducing the experimental results and verifying the proposed visual security defense method. (ZIP) [file pone.0338835.s004.zip › image-attacks/attack_51.png]

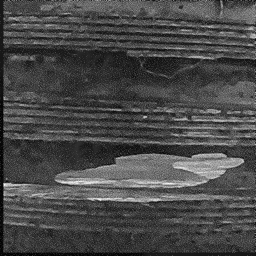

Supplement: S4 File — used in this study, which are essential for reproducing the experimental results and verifying the proposed visual security defense method. (ZIP) [file pone.0338835.s004.zip › image-attacks/attack_52.png]

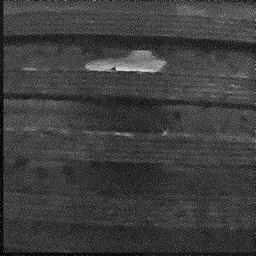

Supplement: S4 File — used in this study, which are essential for reproducing the experimental results and verifying the proposed visual security defense method. (ZIP) [file pone.0338835.s004.zip › image-attacks/attack_53.png]

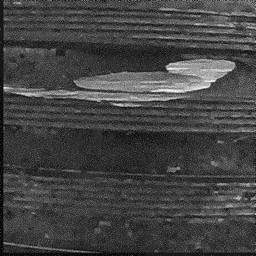

Supplement: S4 File — used in this study, which are essential for reproducing the experimental results and verifying the proposed visual security defense method. (ZIP) [file pone.0338835.s004.zip › image-attacks/attack_54.png]

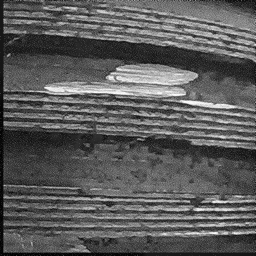

Supplement: S4 File — used in this study, which are essential for reproducing the experimental results and verifying the proposed visual security defense method. (ZIP) [file pone.0338835.s004.zip › image-attacks/attack_55.png]

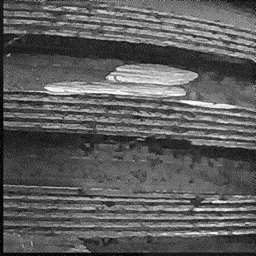

Supplement: S4 File — used in this study, which are essential for reproducing the experimental results and verifying the proposed visual security defense method. (ZIP) [file pone.0338835.s004.zip › image-attacks/attack_56.png]

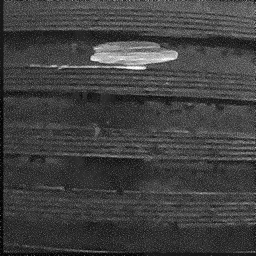

Supplement: S4 File — used in this study, which are essential for reproducing the experimental results and verifying the proposed visual security defense method. (ZIP) [file pone.0338835.s004.zip › image-attacks/attack_57.png]

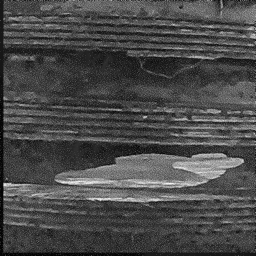

Supplement: S4 File — used in this study, which are essential for reproducing the experimental results and verifying the proposed visual security defense method. (ZIP) [file pone.0338835.s004.zip › image-attacks/attack_58.png]

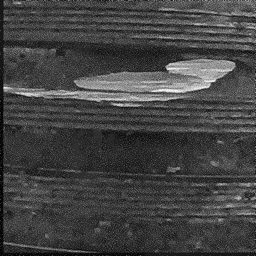

Supplement: S4 File — used in this study, which are essential for reproducing the experimental results and verifying the proposed visual security defense method. (ZIP) [file pone.0338835.s004.zip › image-attacks/attack_59.png]

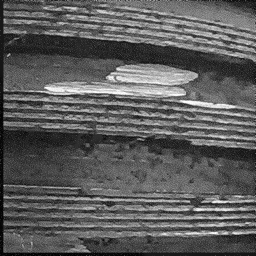

Supplement: S4 File — used in this study, which are essential for reproducing the experimental results and verifying the proposed visual security defense method. (ZIP) [file pone.0338835.s004.zip › image-attacks/attack_6.png]

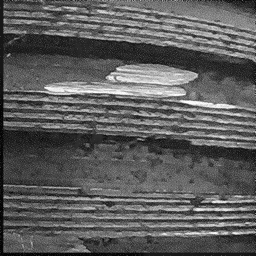

Supplement: S4 File — used in this study, which are essential for reproducing the experimental results and verifying the proposed visual security defense method. (ZIP) [file pone.0338835.s004.zip › image-attacks/attack_60.png]

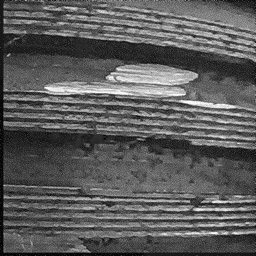

Supplement: S4 File — used in this study, which are essential for reproducing the experimental results and verifying the proposed visual security defense method. (ZIP) [file pone.0338835.s004.zip › image-attacks/attack_61.png]

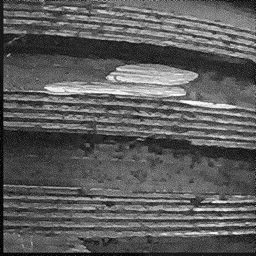

Supplement: S4 File — used in this study, which are essential for reproducing the experimental results and verifying the proposed visual security defense method. (ZIP) [file pone.0338835.s004.zip › image-attacks/attack_62.png]

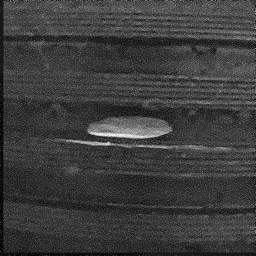

Supplement: S4 File — used in this study, which are essential for reproducing the experimental results and verifying the proposed visual security defense method. (ZIP) [file pone.0338835.s004.zip › image-attacks/attack_63.png]

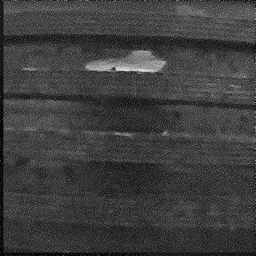

Supplement: S4 File — used in this study, which are essential for reproducing the experimental results and verifying the proposed visual security defense method. (ZIP) [file pone.0338835.s004.zip › image-attacks/attack_64.png]

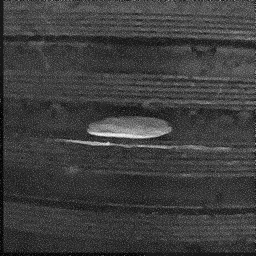

Supplement: S4 File — used in this study, which are essential for reproducing the experimental results and verifying the proposed visual security defense method. (ZIP) [file pone.0338835.s004.zip › image-attacks/attack_65.png]

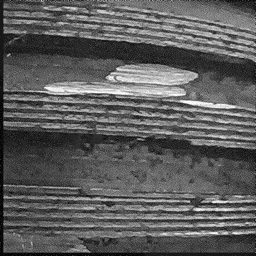

Supplement: S4 File — used in this study, which are essential for reproducing the experimental results and verifying the proposed visual security defense method. (ZIP) [file pone.0338835.s004.zip › image-attacks/attack_66.png]

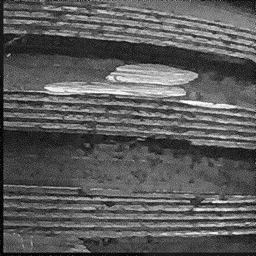

Supplement: S4 File — used in this study, which are essential for reproducing the experimental results and verifying the proposed visual security defense method. (ZIP) [file pone.0338835.s004.zip › image-attacks/attack_67.png]

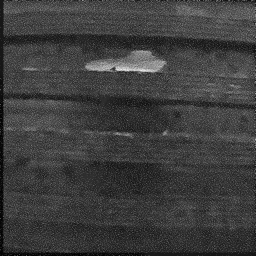

Supplement: S4 File — used in this study, which are essential for reproducing the experimental results and verifying the proposed visual security defense method. (ZIP) [file pone.0338835.s004.zip › image-attacks/attack_68.png]

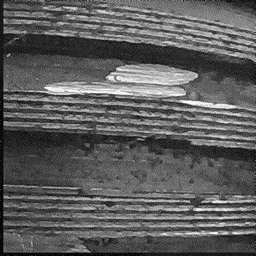

Supplement: S4 File — used in this study, which are essential for reproducing the experimental results and verifying the proposed visual security defense method. (ZIP) [file pone.0338835.s004.zip › image-attacks/attack_69.png]

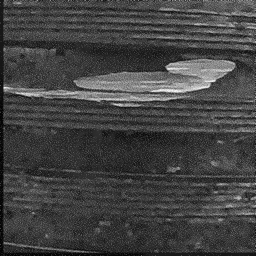

Supplement: S4 File — used in this study, which are essential for reproducing the experimental results and verifying the proposed visual security defense method. (ZIP) [file pone.0338835.s004.zip › image-attacks/attack_7.png]

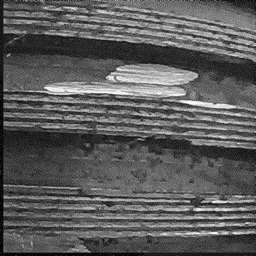

Supplement: S4 File — used in this study, which are essential for reproducing the experimental results and verifying the proposed visual security defense method. (ZIP) [file pone.0338835.s004.zip › image-attacks/attack_70.png]

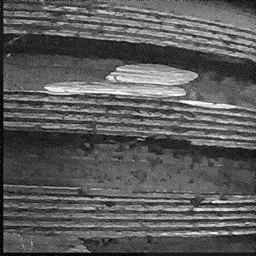

Supplement: S4 File — used in this study, which are essential for reproducing the experimental results and verifying the proposed visual security defense method. (ZIP) [file pone.0338835.s004.zip › image-attacks/attack_71.png]

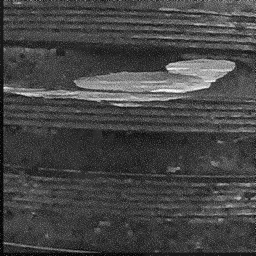

Supplement: S4 File — used in this study, which are essential for reproducing the experimental results and verifying the proposed visual security defense method. (ZIP) [file pone.0338835.s004.zip › image-attacks/attack_72.png]

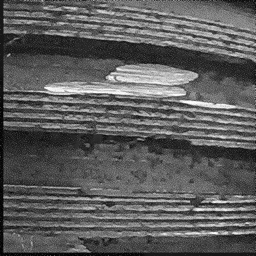

Supplement: S4 File — used in this study, which are essential for reproducing the experimental results and verifying the proposed visual security defense method. (ZIP) [file pone.0338835.s004.zip › image-attacks/attack_73.png]

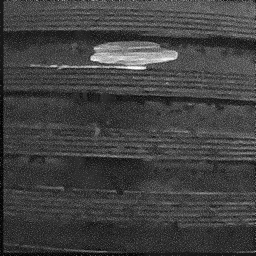

Supplement: S4 File — used in this study, which are essential for reproducing the experimental results and verifying the proposed visual security defense method. (ZIP) [file pone.0338835.s004.zip › image-attacks/attack_74.png]

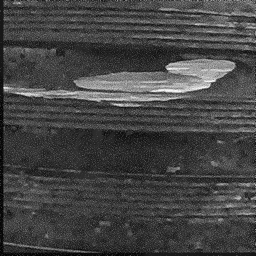

Supplement: S4 File — used in this study, which are essential for reproducing the experimental results and verifying the proposed visual security defense method. (ZIP) [file pone.0338835.s004.zip › image-attacks/attack_75.png]

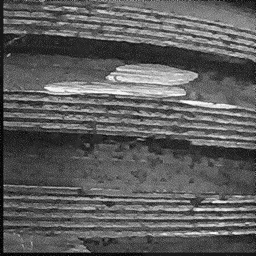

Supplement: S4 File — used in this study, which are essential for reproducing the experimental results and verifying the proposed visual security defense method. (ZIP) [file pone.0338835.s004.zip › image-attacks/attack_76.png]

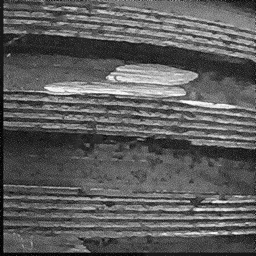

Supplement: S4 File — used in this study, which are essential for reproducing the experimental results and verifying the proposed visual security defense method. (ZIP) [file pone.0338835.s004.zip › image-attacks/attack_77.png]

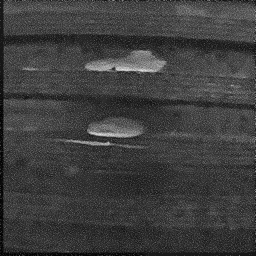

Supplement: S4 File — used in this study, which are essential for reproducing the experimental results and verifying the proposed visual security defense method. (ZIP) [file pone.0338835.s004.zip › image-attacks/attack_78.png]

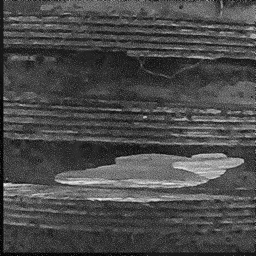

Supplement: S4 File — used in this study, which are essential for reproducing the experimental results and verifying the proposed visual security defense method. (ZIP) [file pone.0338835.s004.zip › image-attacks/attack_79.png]

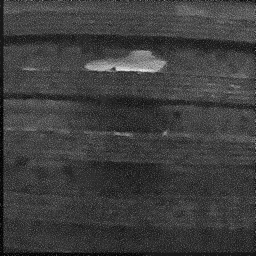

Supplement: S4 File — used in this study, which are essential for reproducing the experimental results and verifying the proposed visual security defense method. (ZIP) [file pone.0338835.s004.zip › image-attacks/attack_8.png]

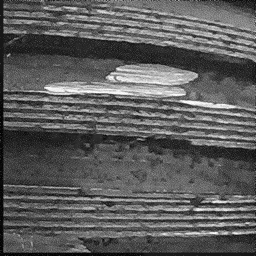

Supplement: S4 File — used in this study, which are essential for reproducing the experimental results and verifying the proposed visual security defense method. (ZIP) [file pone.0338835.s004.zip › image-attacks/attack_80.png]

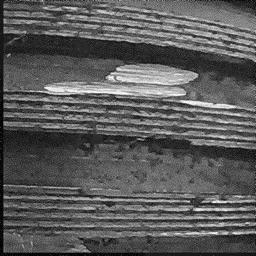

Supplement: S4 File — used in this study, which are essential for reproducing the experimental results and verifying the proposed visual security defense method. (ZIP) [file pone.0338835.s004.zip › image-attacks/attack_81.png]

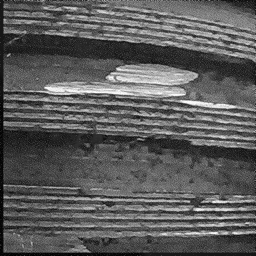

Supplement: S4 File — used in this study, which are essential for reproducing the experimental results and verifying the proposed visual security defense method. (ZIP) [file pone.0338835.s004.zip › image-attacks/attack_82.png]

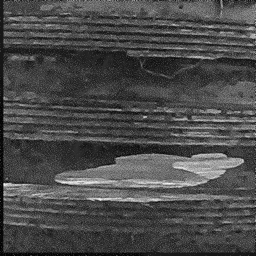

Supplement: S4 File — used in this study, which are essential for reproducing the experimental results and verifying the proposed visual security defense method. (ZIP) [file pone.0338835.s004.zip › image-attacks/attack_83.png]

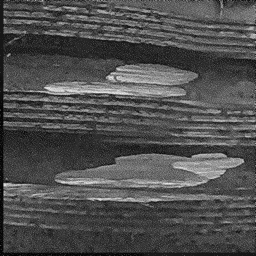

Supplement: S4 File — used in this study, which are essential for reproducing the experimental results and verifying the proposed visual security defense method. (ZIP) [file pone.0338835.s004.zip › image-attacks/attack_84.png]

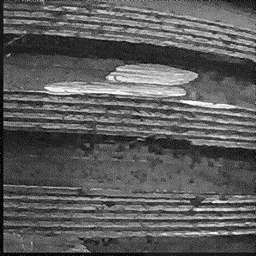

Supplement: S4 File — used in this study, which are essential for reproducing the experimental results and verifying the proposed visual security defense method. (ZIP) [file pone.0338835.s004.zip › image-attacks/attack_85.png]
