# Supplementary figures and images for: Visual security defense for industrial inspection based on computer vision (part 2 of 2)
Source: PLoS One. 2026 Feb 4;21(2):e0338835. doi: 10.1371/journal.pone.0338835 (PMC12872028; doi:10.1371/journal.pone.0338835)

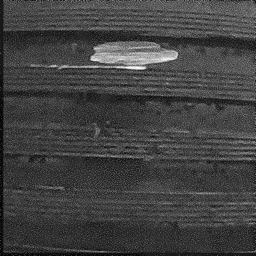

Supplement: S4 File — used in this study, which are essential for reproducing the experimental results and verifying the proposed visual security defense method. (ZIP) [file pone.0338835.s004.zip › image-attacks/attack_86.png]

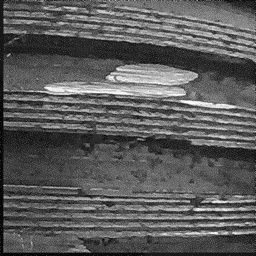

Supplement: S4 File — used in this study, which are essential for reproducing the experimental results and verifying the proposed visual security defense method. (ZIP) [file pone.0338835.s004.zip › image-attacks/attack_87.png]

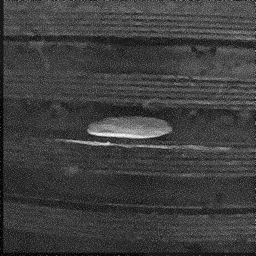

Supplement: S4 File — used in this study, which are essential for reproducing the experimental results and verifying the proposed visual security defense method. (ZIP) [file pone.0338835.s004.zip › image-attacks/attack_88.png]

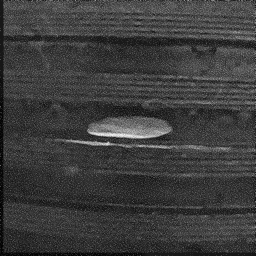

Supplement: S4 File — used in this study, which are essential for reproducing the experimental results and verifying the proposed visual security defense method. (ZIP) [file pone.0338835.s004.zip › image-attacks/attack_89.png]

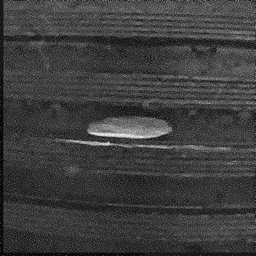

Supplement: S4 File — used in this study, which are essential for reproducing the experimental results and verifying the proposed visual security defense method. (ZIP) [file pone.0338835.s004.zip › image-attacks/attack_9.png]

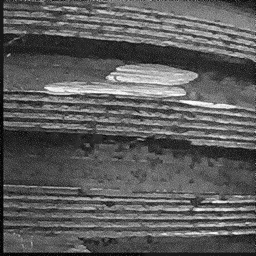

Supplement: S4 File — used in this study, which are essential for reproducing the experimental results and verifying the proposed visual security defense method. (ZIP) [file pone.0338835.s004.zip › image-attacks/attack_90.png]

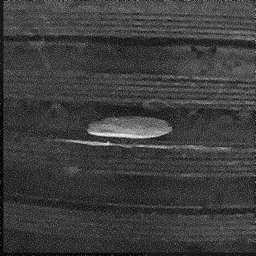

Supplement: S4 File — used in this study, which are essential for reproducing the experimental results and verifying the proposed visual security defense method. (ZIP) [file pone.0338835.s004.zip › image-attacks/attack_91.png]

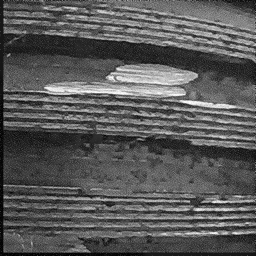

Supplement: S4 File — used in this study, which are essential for reproducing the experimental results and verifying the proposed visual security defense method. (ZIP) [file pone.0338835.s004.zip › image-attacks/attack_92.png]

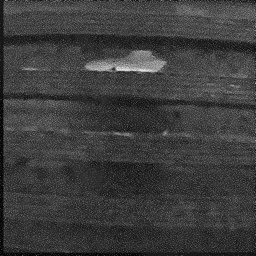

Supplement: S4 File — used in this study, which are essential for reproducing the experimental results and verifying the proposed visual security defense method. (ZIP) [file pone.0338835.s004.zip › image-attacks/attack_93.png]

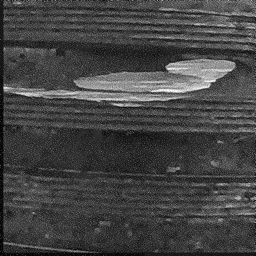

Supplement: S4 File — used in this study, which are essential for reproducing the experimental results and verifying the proposed visual security defense method. (ZIP) [file pone.0338835.s004.zip › image-attacks/attack_94.png]

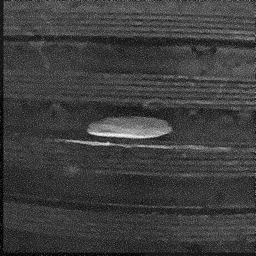

Supplement: S4 File — used in this study, which are essential for reproducing the experimental results and verifying the proposed visual security defense method. (ZIP) [file pone.0338835.s004.zip › image-attacks/attack_95.png]

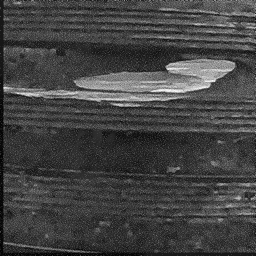

Supplement: S4 File — used in this study, which are essential for reproducing the experimental results and verifying the proposed visual security defense method. (ZIP) [file pone.0338835.s004.zip › image-attacks/attack_96.png]

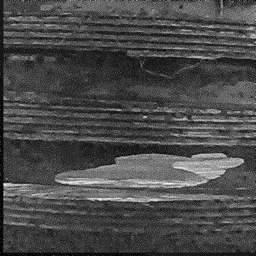

Supplement: S4 File — used in this study, which are essential for reproducing the experimental results and verifying the proposed visual security defense method. (ZIP) [file pone.0338835.s004.zip › image-attacks/attack_97.png]

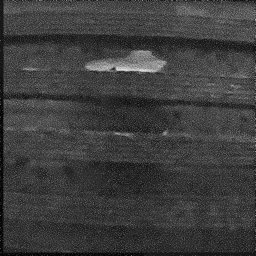

Supplement: S4 File — used in this study, which are essential for reproducing the experimental results and verifying the proposed visual security defense method. (ZIP) [file pone.0338835.s004.zip › image-attacks/attack_98.png]

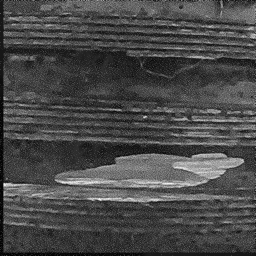

Supplement: S4 File — used in this study, which are essential for reproducing the experimental results and verifying the proposed visual security defense method. (ZIP) [file pone.0338835.s004.zip › image-attacks/attack_99.png]

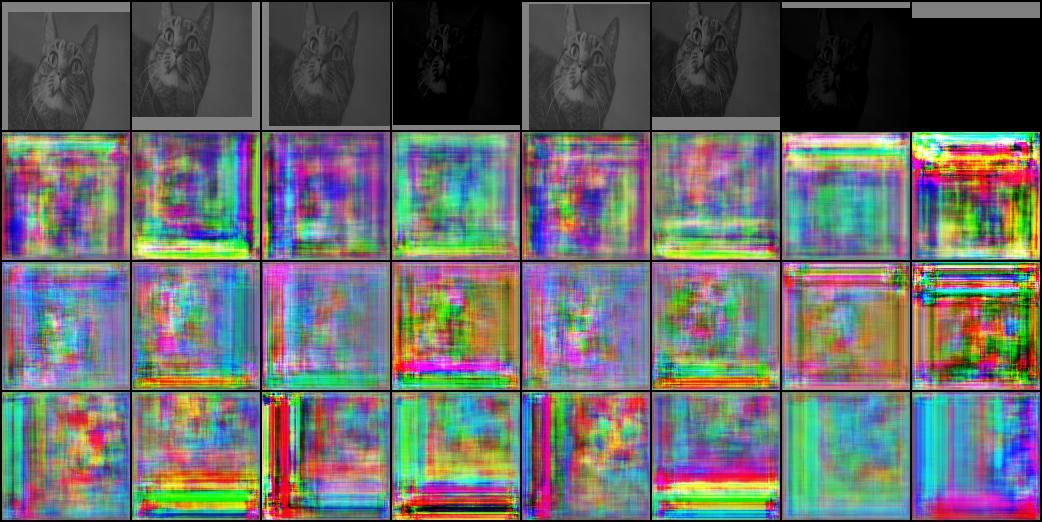

Supplement: S4 File — used in this study, which are essential for reproducing the experimental results and verifying the proposed visual security defense method. (ZIP) [file pone.0338835.s004.zip › image-attacks/rec_0.jpg]

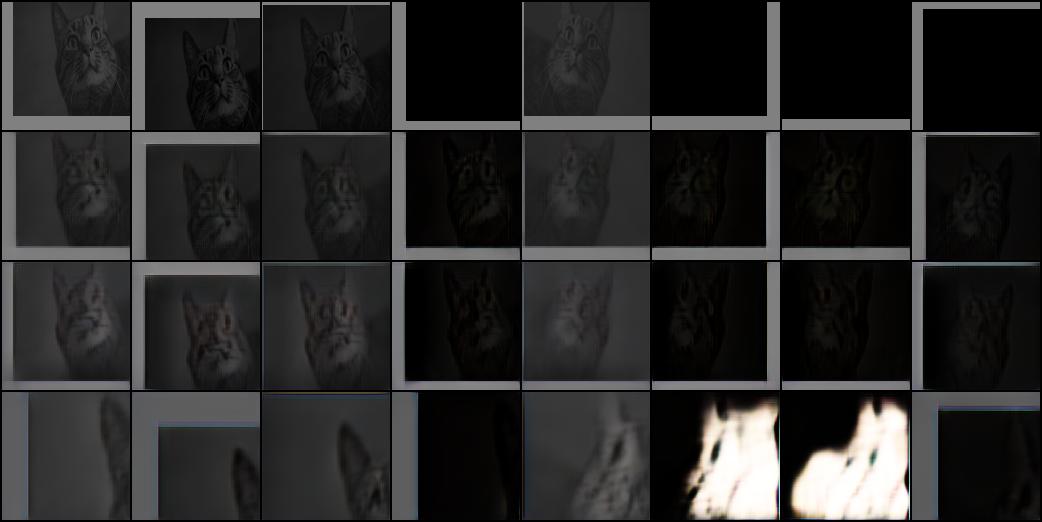

Supplement: S4 File — used in this study, which are essential for reproducing the experimental results and verifying the proposed visual security defense method. (ZIP) [file pone.0338835.s004.zip › image-attacks/rec_1000.jpg]

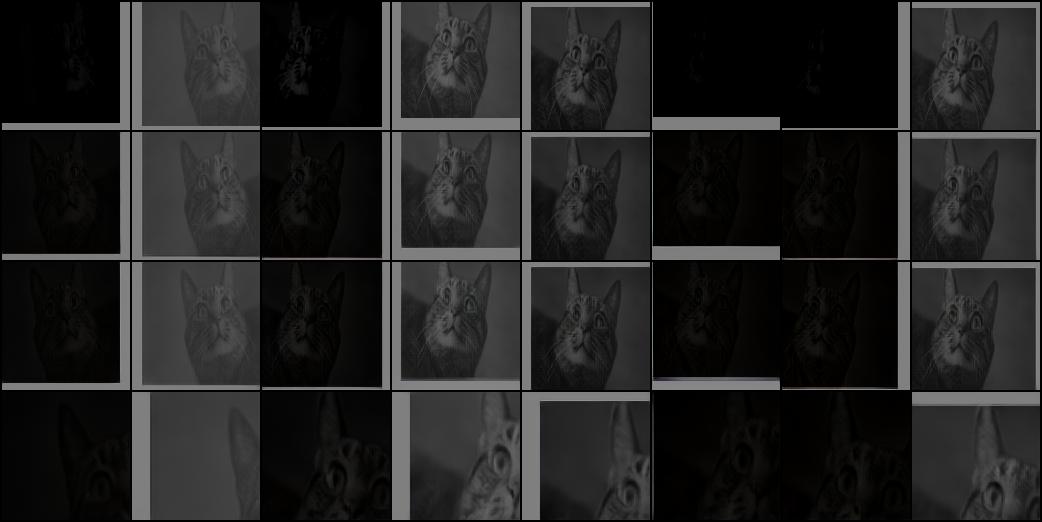

Supplement: S4 File — used in this study, which are essential for reproducing the experimental results and verifying the proposed visual security defense method. (ZIP) [file pone.0338835.s004.zip › image-attacks/rec_10000.jpg]

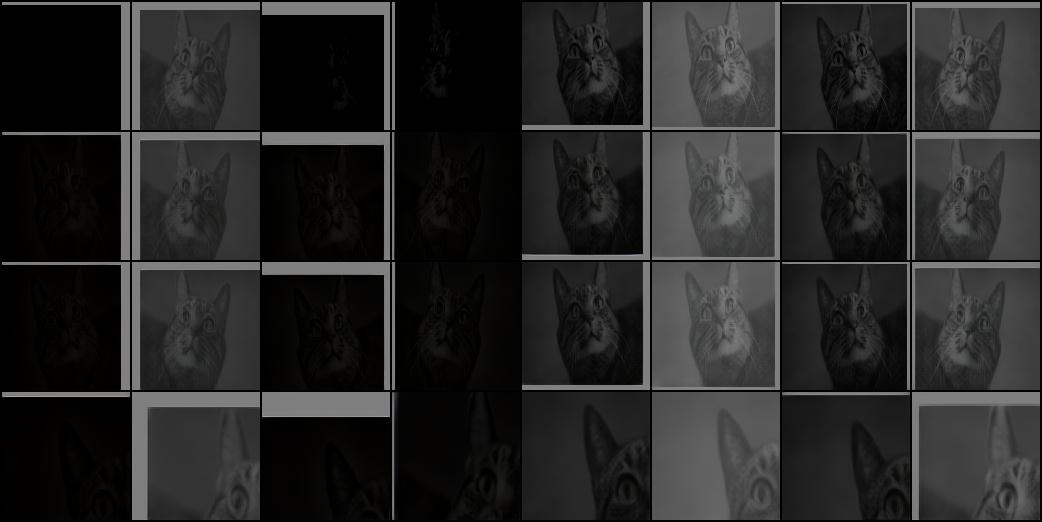

Supplement: S4 File — used in this study, which are essential for reproducing the experimental results and verifying the proposed visual security defense method. (ZIP) [file pone.0338835.s004.zip › image-attacks/rec_11000.jpg]

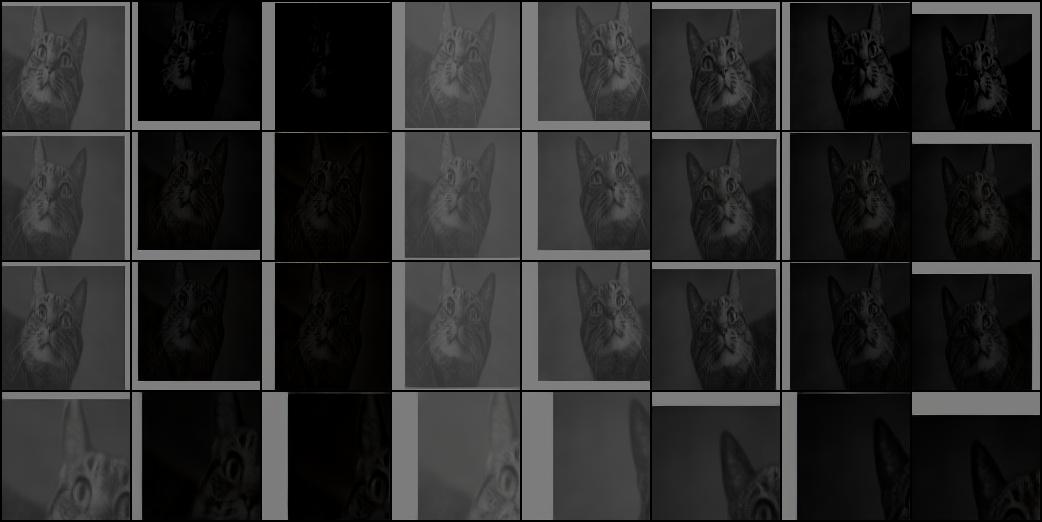

Supplement: S4 File — used in this study, which are essential for reproducing the experimental results and verifying the proposed visual security defense method. (ZIP) [file pone.0338835.s004.zip › image-attacks/rec_12000.jpg]

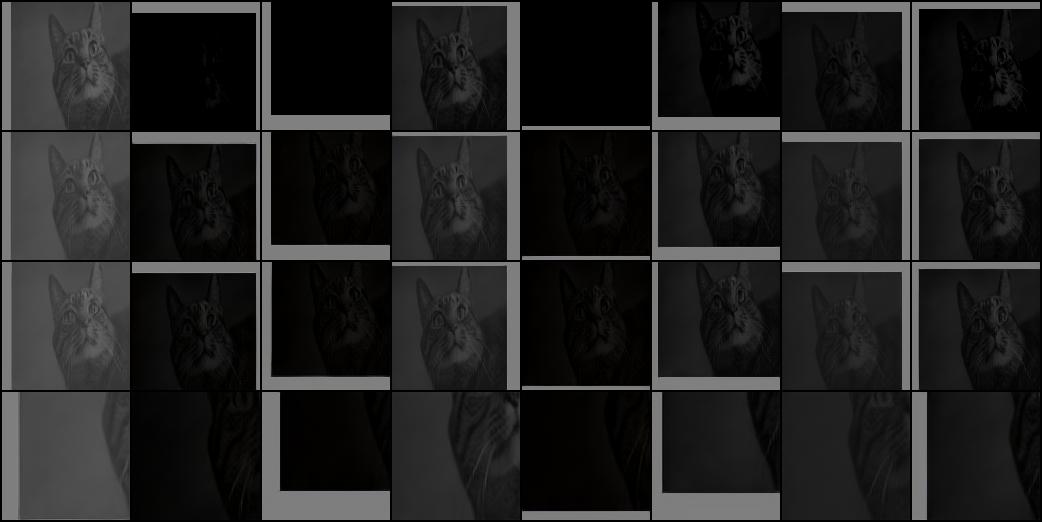

Supplement: S4 File — used in this study, which are essential for reproducing the experimental results and verifying the proposed visual security defense method. (ZIP) [file pone.0338835.s004.zip › image-attacks/rec_13000.jpg]

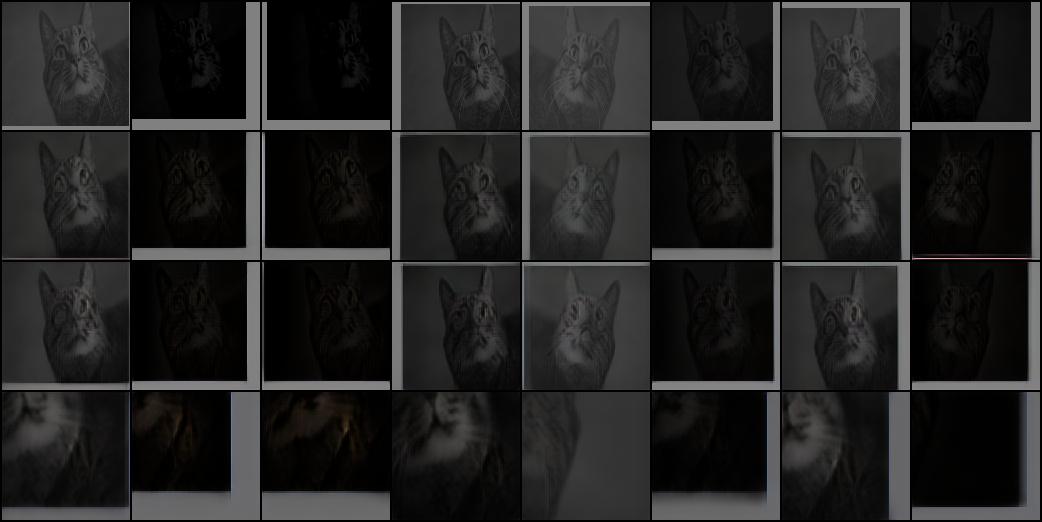

Supplement: S4 File — used in this study, which are essential for reproducing the experimental results and verifying the proposed visual security defense method. (ZIP) [file pone.0338835.s004.zip › image-attacks/rec_2000.jpg]

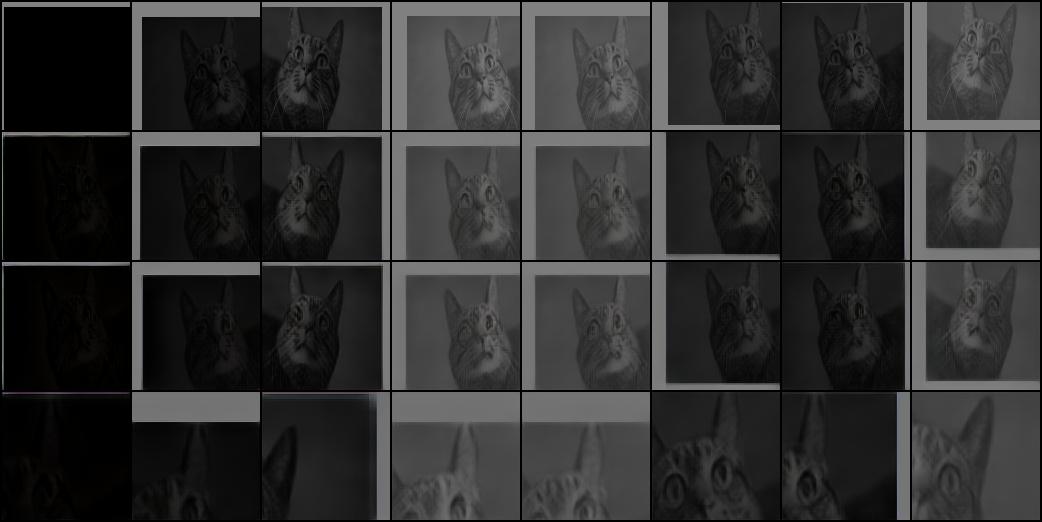

Supplement: S4 File — used in this study, which are essential for reproducing the experimental results and verifying the proposed visual security defense method. (ZIP) [file pone.0338835.s004.zip › image-attacks/rec_3000.jpg]

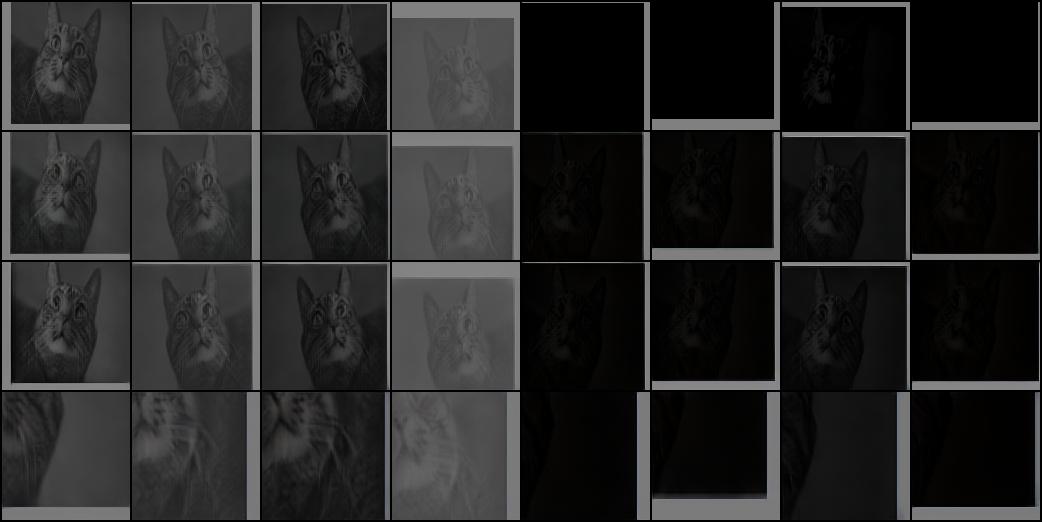

Supplement: S4 File — used in this study, which are essential for reproducing the experimental results and verifying the proposed visual security defense method. (ZIP) [file pone.0338835.s004.zip › image-attacks/rec_4000.jpg]

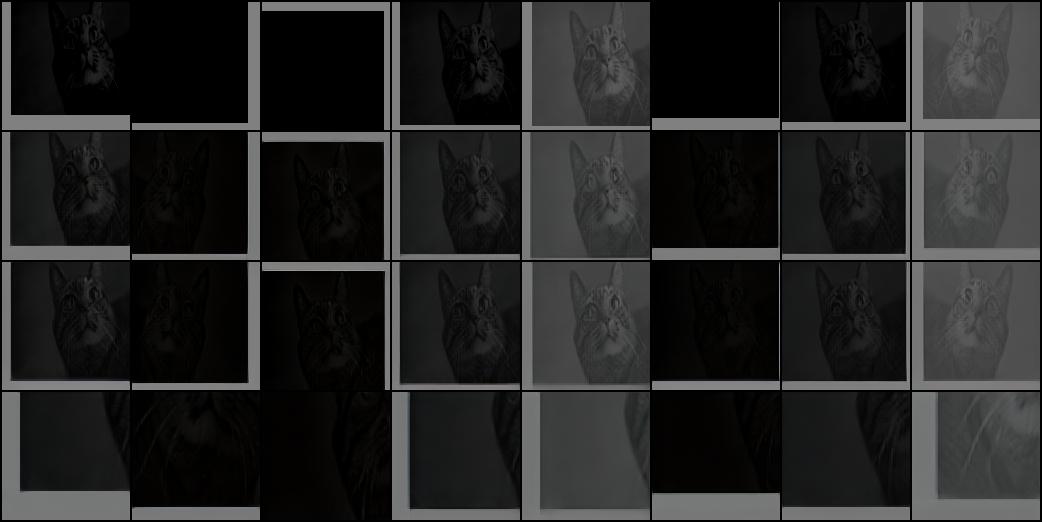

Supplement: S4 File — used in this study, which are essential for reproducing the experimental results and verifying the proposed visual security defense method. (ZIP) [file pone.0338835.s004.zip › image-attacks/rec_5000.jpg]

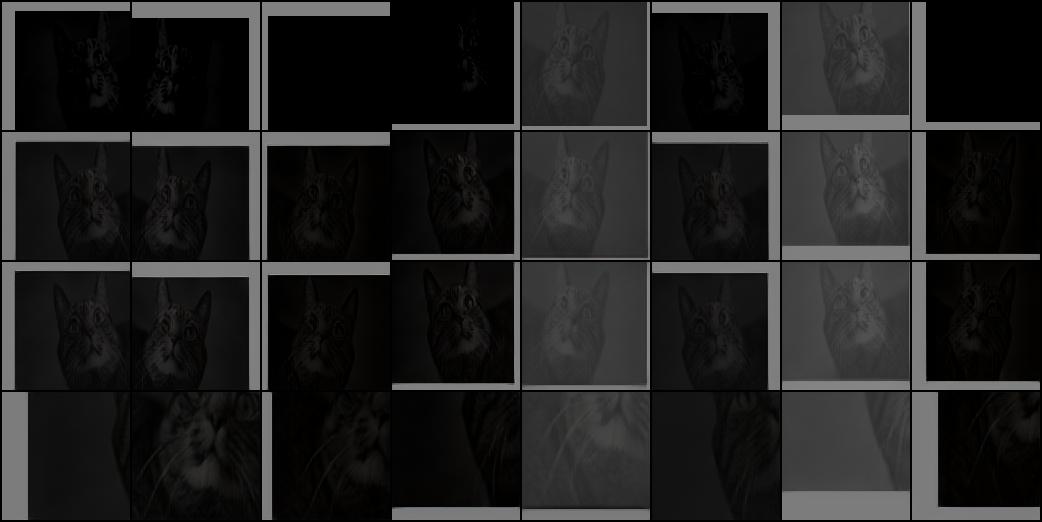

Supplement: S4 File — used in this study, which are essential for reproducing the experimental results and verifying the proposed visual security defense method. (ZIP) [file pone.0338835.s004.zip › image-attacks/rec_6000.jpg]

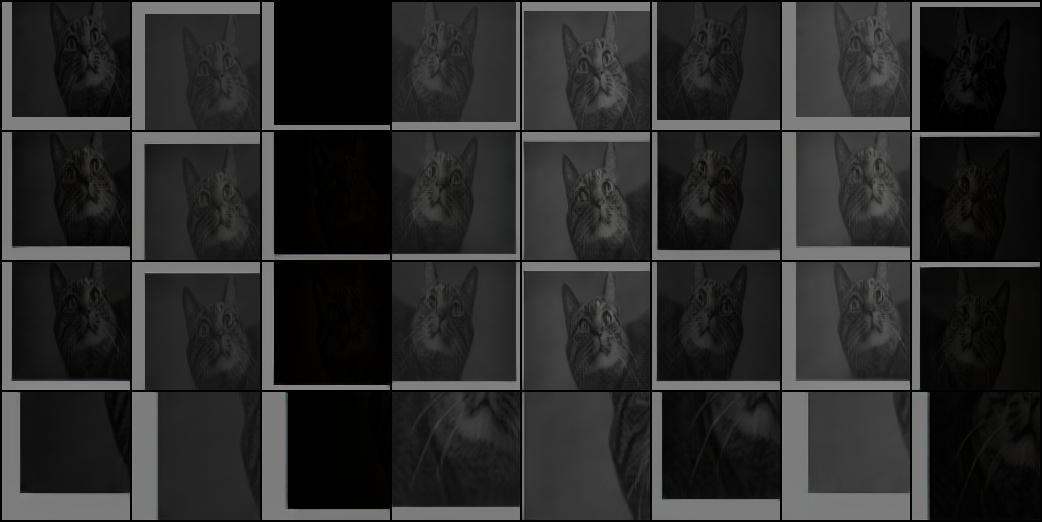

Supplement: S4 File — used in this study, which are essential for reproducing the experimental results and verifying the proposed visual security defense method. (ZIP) [file pone.0338835.s004.zip › image-attacks/rec_7000.jpg]

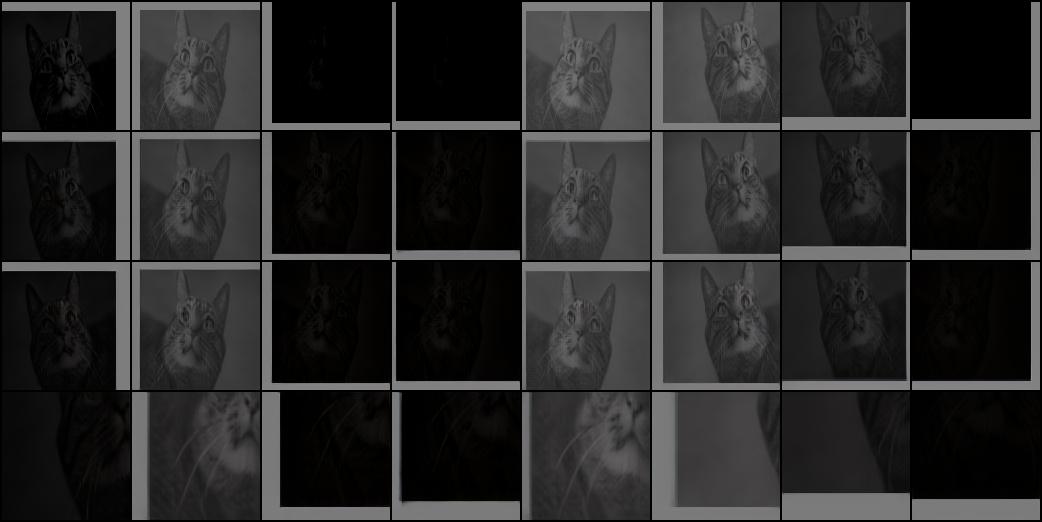

Supplement: S4 File — used in this study, which are essential for reproducing the experimental results and verifying the proposed visual security defense method. (ZIP) [file pone.0338835.s004.zip › image-attacks/rec_8000.jpg]

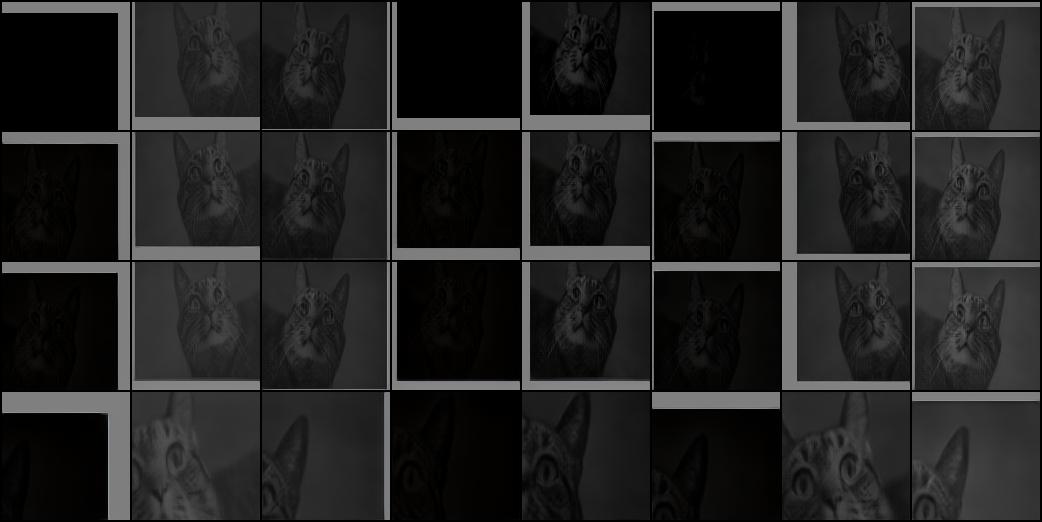

Supplement: S4 File — used in this study, which are essential for reproducing the experimental results and verifying the proposed visual security defense method. (ZIP) [file pone.0338835.s004.zip › image-attacks/rec_9000.jpg]
